# An improved FOX optimization algorithm using adaptive exploration and exploitation for global optimization

**DOI:** 10.1371/journal.pone.0331965

**Published:** 2025-09-18

**Authors:** Mahmood A. Jumaah, Yossra H. Ali, Tarik A. Rashid

**Affiliations:** 1 College of Computer Science, University of Technology, Baghdad, Iraq; 2 Department of Computer Science and Engineering, AIIC, University of Kurdistan Hewlêr, Erbil, Iraq; Hanshan Normal University, CHINA

## Abstract

Optimization algorithms are essential for solving many real-world problems. However, challenges such as getting trapped in local minima and effectively balancing exploration and exploitation often limit their performance. This paper introduces an improved variation of the FOX optimization algorithm (FOX), termed Improved FOX (IFOX), incorporating a new adaptive method using a dynamically scaled step-size parameter to balance exploration and exploitation based on the current solution’s fitness value. The proposed IFOX also reduces the number of hyperparameters by removing four parameters (C1, C2, a, Mint) and refines the primary equations of FOX. To evaluate its performance, IFOX was tested on 20 classical benchmark functions, 61 benchmark test functions from the congress on evolutionary computation (CEC), and ten real-world problems. The experimental results showed that IFOX achieved a 40% improvement in overall performance metrics over the original FOX. Additionally, it achieved 880 wins, 228 ties, and 348 losses against 16 optimization algorithms across all involved functions and problems. Furthermore, non-parametric statistical tests, including the Friedman and Wilcoxon signed-rank tests, confirmed its competitiveness against recent and state-of-the-art optimization algorithms, such as LSHADE and NRO, with an average rank of 5.92 among 17 algorithms. These findings highlight the significant potential of IFOX for solving diverse optimization problems, establishing it as a competitive and effective optimization algorithm.

## Introduction

Researchers in computer science define optimization as a systematic method to improve systems for problem-solving with maximum performance. It involves identifying the best solutions for a specific problem [1]. However, most real-world problems (RWPs) can be optimized, and with the rapid advancement of computational techniques and increasing computing power, many sectors have experienced significant growth compared to the past [2,3]. For instance, medicine, engineering, agriculture, commerce, finance, and entertainment [4,5]. An optimizer is an algorithm designed to solve optimization problems by iteratively searching for the best solution to minimize or maximize an objective function [6,7]. However, optimizers differ in their problem-solving approach, execution speed, complexity, balance between exploration and exploitation, underlying inspiration, and solution diversity [8,9]. Improving an optimization algorithm’s performance without increasing resource usage means solving problems efficiently with the same processing time and memory consumption to reach the optimal solution, which remains a significant challenge.

This paper concerns the FOX optimization algorithm (FOX), a recent method developed in 2022 by Mohammed and Rashid [10]. Although FOX has outperformed several optimization algorithms, its broader significance remains limited due to certain limitations. In particular, it has not been evaluated against state-of-the-art (SOTA) optimizers or tested on a sufficiently wide range of benchmark functions. Additionally, it utilizes a static approach to balance exploration and exploitation by fixing a ratio of 0.5% for each phase. This does not match the nature of many optimization problems, as some issues need more exploration, and others need more exploitation. For instance, the search space has many local optima in a Rastrigin complex benchmark test function [11]. Thus, FOX may either converge prematurely or waste resources on unnecessary exploration, as described in the Limitations in FOX optimization algorithm. Additionally, the mathematical equations used in FOX for both the exploration and exploitation phases were acknowledged by its authors as improvable, particularly in terms of jump modeling and prey distance estimation. These limitations reduce its adaptability under challenging problems and thus reduce its overall performance. Therefore, the primary objective of this study is to enhance the performance of FOX by addressing its limitations and proposing an improved variation, improved FOX (IFOX). We aim to decrease the number of equations and parameters and suggest a completely adaptive method to balance exploration and exploitation. The main contributions of this paper are:

1. Replacing constrained equations in FOX with more effective alternatives and reducing the number of hyperparameters.
2. Proposing a new fitness-based adaptive method for better exploration and exploitation balance.
3. The proposed IFOX outperformed the original FOX and most of the compared algorithms across various benchmark functions and engineering design problems.

The IFOX was evaluated using a wide range of 81 benchmark test functions to ensure rigorous performance evaluations. These included 20 classical functions (ten unimodal and ten multimodal) and 61 congress on evolutionary computation (CEC)-based functions from CEC 2017, CEC 2019, CEC 2021, and CEC 2022, which are widely used in optimization algorithms assessments. Additionally, ten RWPs were employed to demonstrate the applicability of IFOX in real-world, as explained in Real-world problems. However, the experimental results show that the proposed IFOX outperforms the basic FOX while achieving performance comparable to various recent and SOTA optimization algorithms.

The rest of this paper is organized as follows: The Literature review provides a literature review of existing improved optimization algorithms and their applications, in addition to our previous optimization algorithms. The Methodology describes the methods used in this paper to develop the IFOX. The Results lists the results obtained from the experiments. The Discussion discusses, analyzes, and compares the findings. Finally, The Conclusion concludes and summarizes the key contributions and limitations and suggests future work directions.

## Literature review

Optimization algorithms are advancing rapidly, making significant progress in many industries and fields, especially in engineering and computer science [12]. New algorithms are frequently proposed and subsequently improved by researchers to enhance their global problem-solving capabilities or to adapt them to specific types of problems [13–15]. Given the continuous emergence of new challenges, the development and enhancement of optimization algorithms remain a highly relevant and hot-topic area of research [16,17]. The following paragraphs present optimization algorithms categorized by their inspiration source, followed by recent and SOTA algorithms.

Optimization algorithms can be classified into many categories: swarm-based algorithms, evolutionary algorithms, physics or chemistry-based algorithms, or in hybrid form. However, algorithms that take inspiration from the collective actions of species like ants, fish, bees, wasps, and birds are known as swarm intelligence algorithms [18]. This concept comes from the natural group behavior of these species as they search for food. A key feature of swarm-based algorithms is that they rely on simple agents that are not complex. These agents work together through indirect communication and move within the decision space to find better solutions [19]. For instance, shrike optimization algorithm (SHOA) is a popular optimization method that takes inspiration from how shrike birds migrate, keep alive and reproduce. Using this algorithm, both stages of exploration and exploitation are structured, just like birds feed and take care of their young until they are able to look after themselves. A mathematical expression was developed for the algorithm and it was also tested on 41 different problems in optimization. This meant using 19 benchmark test functions, 10 CEC-2019 and 12 CEC-2022 and also four engineering design problems with constraints and no constraints. According to the research, SHOA shows excellent performance when handling tasks that need to optimize multiple variables together [20].

Similarly, grey wolf optimization (GWO) is commonly used algorithm that take his inspiration from wolves foraging behavior. Various studies focused on improving the performance of the basic GWO. For instance, a velocity-aided grey wolf optimizer 90 (VAGWO) was proposed to enhance global search and avoid rash convergence [21]. Another improvement applied an exponential decay approach to better control the transition from exploration to exploitation throughout the optimization process by introducing a differential perturbation using three omega wolves and applying unified *A* and *C* parameters to promote exploration during exploitation [22]. Furthermore, the GWO has been modified to solve feature selection in high-dimensional data by integrating ReliefF and Copula entropy during initialization. It utilizes a competitive guidance strategy for flexible search and a differential evolution-based method to enhance leader positioning and avoid local optima [23]. Additionally, an improved version of GWO called variable weights grey Wolf optimization (VWGWO) has been proposed, utilizes social hierarchies to avoid local optima and performs well in high-dimensional scenarios. It used a modified control parameter equation to reduce the local optima trap by proposing that in the standard GWO, all dominant wolves influence the search equally, but this contradicts the social hierarchy of grey wolves. Since the alpha is the true leader, its position should have the highest influence, followed by the beta and the delta. Early in the search, the alpha’s position should either be the sole reference or weighted more heavily to reflect its dominant role [24]. The results of VWGWO indicate that it surpasses basic GWO, ant lion optimization (ALO) and particle swarm optimization (PSO) across several benchmark functions, which makes it a suitable choice for comparison and analysis purposes in this study.

Bio-inspired optimization algorithms, inspired by biological phenomena such as evolution and animal behavior, are widely used across many disciplines [25]. For example, Brown-bear optimization algorithm (BBOA) uses brown bear communication capabilities to balance search exploration and exploitation through its sniffing mechanisms and footprint markings, which prove superiority in minimizing power system expenses [26]. An additional bio-inspired algorithm, named dragonfly algorithm (DA), inspired by the hunting and migration behaviors of dragonflies, simulates two key behaviors: static hunting, where dragonflies create a small, static swarm to encircle prey, and dynamic migration, where they form more prominent groups to travel long distances [27]. The DA has been extended to its binary form binary dragonfly algorithm (BDA) to handle binary optimization tasks such as feature selection (FS). It updates the main coefficients—separation, alignment, cohesion, food attraction, and enemy distraction—using random values, aiming to imitate the natural movement and interactions of dragonflies in a binary space. Furthermore, experimental results demonstrate that the BDA achieves superior performance, selects fewer yet more relevant features, and attains better objective function values across 18 well-known benchmark datasets [28]. Moreover, recent study introduces an improved particle swarm optimization (IPSO) for automated guided vehicles (AGV) path planning problem in a one-line production. This improved algorithm incorporates a novel coding method, crossover operation, and mutation mechanism to refine solution quality. The experiments showed the superior performance of IPSO over the conventional optimization algorithms, underlining its efficacy in minimizing transportation time and avoiding local optima [31].

Evolutionary algorithms are stochastic population-based metaheuristics widely used to solve complex and various issues in domains such as optimization, system modeling, and engineering design. This advancement made evolutionary computation an independent research domain. Hybrid evolutionary approaches combine various optimization algorithms to enhance performance. For instance, in a recent study, researchers investigated the limitations of traditional genetic algorithms (GA), which, despite being powerful tools for global optimization based on evolution theory, traditional GA tend to have suboptimal convergence speed and efficiency. However, to tackle this issue, the study suggests an improved adaptive genetic algorithm (IAGA) that improves the crossover and mutation probabilities adaptively based on the fitness value of individuals in the group. The results of evaluating IAGA on traveling salesman problem (TSP) problems achieves faster convergence and improved efficiency [32]. Moreover whale optimization algorithm (WOA) is improved through hybridization with differential evolution (DE) to proposes the improved whale optimization algorithm (IWOA) by combining WOA’s exploitation with DE’s exploration, addressing WOA’s early convergence issues. Additionally, an extended version, IWOA+, incorporates re-initialization and adaptive parameter control. Comparative experiments demonstrate that both IWOA and IWOA+ outperform other algorithms regarding solution quality and convergence across 25 benchmark test functions [33].

The SOTA algorithms represent current benchmarks in optimization research. Among SOTA evolutionary algorithms, Linear population size reduction success-history based adaptive differential evolution (LSHADE) enhances DE by incorporating a linear population size reduction strategy and adaptive parameter control mechanisms. This approach significantly boosts convergence speed and robustness, consistently achieving superior results across complex benchmark suites like CEC 2014 and CEC 2017 [34]. Furthermore, the adaptive LSHADE (ALSHADE) algorithm extends LSHADE by adaptively adjusting strategies and parameters during optimization runs. It has shown significant performance improvements in solving challenging optimization problems from CEC 2017 and CEC 2020 benchmark test functions [35]. Additionally, the covariance matrix self-adaptation evolutionary algorithm (CMA-ES) adapts the covariance matrix of the mutation distribution dynamically, allowing efficient learning of the problem landscape structure. It is widely recognized for its robustness and reliability in solving complex global optimization problems, demonstrating superior results in various benchmark test suites, including CEC competitions and numerous real-world applications [36].

Several recent optimization algorithms further advance the optimization landscape, highlighting the continuous innovation in metaheuristic design. These include crested porcupine optimizer (CPO) is a bio-inspired algorithm that operates on porcupines’ defensive patterns and achieves population reduction cycling for exploration and exploitation control [37]; the coati optimization algorithm (COA) is inspired by two distinct natural behaviors of coatis [38]; the coati optimization algorithm (COA) introduces a novel three-phase behavioral model inspired by hippopotamus activities [39]; the nuclear reaction optimization (NRO) is physics-inspired algorithm of nuclear fission and fusion phases [40]; the lagrange elementary optimization (LEO) is a self-adaptive evolutionary method motivated by the precision of vaccinations using the albumin quotient of human blood. In LEO, intelligent search agents are developed based on their fitness values following a genetic crossover process. These gene-based mechanisms guide the agents through both the exploration and exploitation processes. The core idea and motivation behind LEO are introduced in [41]; the aquila optimizer (AO), inspired by eagle hunting strategies [42]; the eptile search algorithm (RAS), based on reptilian search behaviors [43]; the artificial gorilla troops optimizer (GTO), modeling social dynamics of gorilla troops [44]; the slime mould algorithm (SMA), derived from the foraging patterns of slime moulds [45]; the barnacles mating optimizer 186 (BMO), reflecting barnacle mating behavior [46]; the bear smell search algorithm 187 (BSSA), inspired by bear olfactory tracking [47]; the black widow optimization algorithm 188 (BWOA), simulating black widow spider mating and cannibalism [48]; the manta ray 189 foraging optimization (MRFO), based on manta ray foraging [49]; the marine predators 190 algorithm (MPA), utilizing predator motion patterns like lévy and brownian movements [50]; and the mayfly algorithm (MA), driven by the mating behavior of mayflies [51]. These optimization algorithms are emphasized in recent surveys [52–54], and demonstrate strong potential in solving complex optimization challenges.

Table 1 presents the SOTA and recent optimization algorithms reviewed, including the name, type, inspiration or main features, and evaluation method. The literature review showed that many algorithms have been improved from their basic form to overcome challenges and limitations and achieve better performance. Common limitations include tuning parameters, escaping local optima, and balancing exploration and exploitation. Based on these observations and an analysis of the limitations of the FOX, as will be explained in the Limitations in FOX optimization algorithm, this study aims to address the existing gaps by proposing an improved and more reliable variation of FOX, capable of enhancing search efficiency by proposing a new method for balancing exploration and exploitation.

**Table 1:** Summary of recent and state-of-the-art optimization algorithms.

Algorithm	Type	Main Feature/Inspiration	Evaluated On	Ref.
ACO	Swarm-based	Classic pheromone trail search	TSP	[55]
ALSHADE	Evolutionary	Self-tuned LSHADE	CEC 2017, CEC 2020	[35]
AO	Bio-inspired	Eagle hunting strategies	CEC 2021	[42]
BDA	Bio-inspired	Binary dragonfly for feature selection	18 FS datasets	[28]
BBOA	Bio-inspired	Bear olfactory tracking	Power systems	[26]
BMO	Bio-inspired	Barnacle mating process	CEC 2022	[46]
BSSA	Bio-inspired	Bear smell tracking	CEC 2022	[47]
BWOA	Bio-inspired	Widow spider mating/cannibalism	CEC 2020	[48]
CMA-ES	Evolutionary	Covariance-based mutation	CEC, real-world	[36]
COA	Bio-inspired	Coati foraging and climbing	CEC 2020	[38]
CPO	Bio-inspired	Porcupine defensive behavior	CEC 2021	[37]
DA	Bio-inspired	Swarm hunting and migration	Standard functions	[27]
FOX	Bio-inspired	Distance-controlled exploration	CEC 2019	[10]
GA	Evolutionary	Canonical genetic operators	General	[32]
GTO	Bio-inspired	Gorilla troop behavior	CEC 2021	[44]
GWO-FS	Swarm-based	Feature selection with entropy initialization	High-dim FS tasks	[23]
HO	Bio-inspired	Hippopotamus activity modeling	CEC 2022	[39]
IAGA	Evolutionary	Fitness-based adaptive GA	TSP	[32]
SHOA	Swarm-based	mimics shrike bird behavior for efficient optimization	CEC 2019, CEC 2022	[20]
IPSO	Bio-inspired	Enhanced PSO for AGV routing	Real-world AGV	[31]
IWOA	Hybrid	DE-exploration + WOA exploitation	CEC 2017, CEC 2020	[33]
IWOA+	Hybrid	IWOA with reinitialization	CEC 2020	[33]
LEO	Bio-inspired	Learning via albumin quotient	CEC 2023	[41]
LSHADE	Evolutionary	Adaptive DE with shrinking population	CEC 2014, CEC 2017	[34]
MA	Bio-inspired	Mayfly drift and mating	CEC 2021	[51]
MPA	Bio-inspired	Predator Lévy and Brownian paths	CEC 2021	[50]
MRFO	Bio-inspired	Manta ray motion modes	CEC 2020	[49]
NRO	Physics-based	Nuclear reaction phases	CEC 2020	[40]
RSA	Bio-inspired	Reptile-based adaptive movement	CEC 2022	[43]
SMA	Bio-inspired	Slime mould foraging	CEC 2020	[45]
VAGWO	Swarm-based	Velocity-guided wolf search	CEC 2019	[21]
VWGWO	Swarm-based	Weighted leadership in wolf hierarchy	CEC 2017, CEC 2020	[24]

## Methodology

This section presents the methods for improving the FOX. It begins by describing the benchmark test functions and RWPs used to evaluate the performance of the proposed algorithm. The subsequent section analyzes the basic FOX and its limitations, followed by a detailed description of the proposed improvements introduced in IFOX, along with supporting algorithms and flowcharts.

### Benchmark test functions

This section presents the benchmark test function that includes classical and CEC-based from the CEC 2017, 2019, 2021, and 2022. However, a specific optimization task identifier (TID) will used instead of the full name of the function to increase the clarity of results and discussion presentation. Classical functions will be denoted as *CL*, and CEC-based functions are refered as follows: *C17* for CEC 2017, *C19* for CEC 2019, *C21* for CEC 2021, and *C22* for CEC 2022.

Classical benchmark test functions are widely used in computational optimization to evaluate and compare the performance of algorithms. These functions serve as standardized tests due to their diverse mathematical properties, such as modality, separability, and dimensionality. Unimodal functions, characterized by a single global minimum, assess the exploitation capability of an algorithm—its ability to converge to the optimum in smooth landscapes. In contrast, multimodal functions contain multiple local minima and evaluate the exploration capability of algorithms, such as avoiding early convergence and determining the global optimum in complex search spaces. In this paper, 20 benchmark functions were selected, including ten unimodal and ten multimodal functions. These functions were chosen for their varied mathematical properties and recognition in the optimization literature [56–58]. On the other hand, The CEC is considered one of the most significant conferences within evolutionary computation, managed by IEEE, which presents complex benchmark test functions. These functions are more complicated than classical ones created to closely mimic real-world optimization challenges. They incorporate a variety of transformations and hybridizations to increase the complexity. Specifically, these functions are constructed using the following features. *Shifting* involves moving the global optimum to another point than the origin, thereby allowing algorithms to avoid any bias that centers around the origin. In addition, *rotation* refers to the coordinate system of the function being rotated to introduce dependencies among the decision variables, making the problem non-separable and more challenging. Moreover, *hybridization* combines different classical functions through weighted sums or division of decision space to form unified functions. The implementation of hybridization introduces various landscape features alongside multiple modes throughout other regions. Furthermore, *composition* combines two or more transformed functions into one entity, creating an unstable search landscape that misguides the decision-making process. Finally, *dimension* and range variability mean the test functions cover a wide range of dimensions and decision variable bounds to assess scalability and robustness [59]. The Table 2 provides 82 classical and CEC-based functions as follows: 20 classical [58], 29 from CEC 2017 [60], ten from CEC 2019 [61], ten from CEC 2021 [62], and 12 from the CEC 2022 [63]. The implementations of the classical and CEC-based benchmark test functions used in this study are available at https://github.com/thieu1995/opfunu.

**Table 2:** Summary of classical and CEC-based benchmark test functions used in this study.

TID	Name	Type	Range	Dim	$f_{min}$
CL1	Ackley 02 function	Unimodal	[-32, 32]	2	-200
CL2	Beale function	Unimodal	[-4.5, 4.5]	2	0
CL3	Booth function	Unimodal	[-10, 10]	2	0
CL4	Chen Bird function	Unimodal	[-500, 500]	2	-2000
CL5	Chung Reynolds function	Unimodal	[-100, 100]	2	0
CL6	Cube function	Unimodal	[-10, 10]	2	0
CL7	Dixon & Price function	Unimodal	[-10, 10]	2	0
CL8	Brent function	Unimodal	[-10, 10]	2	0
CL9	Leon function	Unimodal	[-1.2, 1.2]	2	0
CL10	Matyas function	Unimodal	[-10, 10]	2	0
CL11	Ackley 01 function	Multimodal	[-35, 35]	2	0
CL12	Adjiman function	Multimodal	[-1, 2]	2	-2.022
CL13	Alpine01 function	Multimodal	[-10, 10]	2	0
CL14	Bird function	Multimodal	[-6.2, 6.2]	2	-106.7
CL15	Camel 3-Hump function	Multimodal	[-5, 5]	2	0
CL16	Dolan function	Multimodal	[-100, 100]	5	0
CL17	Exponential function	Multimodal	[-1, 1]	2	-1
CL18	Griewank function	Multimodal	[-100, 100]	2	0
CL19	Hartman 3 function	Multimodal	[0, 1]	3	-3.863
CL20	McCormick	Multimodal	[-1.5, 4]	2	-1.913
C17F1	Shifted and Rotated Bent Cigar function	Unimodal	[-100.0, 100.0]	30	100
C17F2	Shifted and Rotated Zakharov function	Unimodal	[-100, 100]	30	200
C17F3	Shifted and Rotated Rosenbrock’s function	Multimodal	[-100, 100]	30	300
C17F4	Shifted and Rotated Rastrigin’s function	Multimodal	[-100, 100]	30	400
C17F5	Shifted and Rotated Schaffer’s F7 function	Multimodal	[-100, 100]	30	500
C17F6	Shifted and Rotated Lunacek Bi-Rastrigin’s function	Multimodal	[-100, 100]	30	600
C17F7	Shifted and Rotated Non-Continuous Rastrigin’s function	Multimodal	[-100, 100]	30	700
C17F8	Shifted and Rotated Levy function	Multimodal	[-100, 100]	30	800
C17F9	Shifted and Rotated Schwefel’s function	Multimodal	[-100, 100]	30	900
C17F10	Hybrid function 1	Hybrid	[-100, 100]	30	1000
C17F11	Hybrid function 2	Hybrid	[-100, 100]	30	1100
C17F12	Hybrid function 3	Hybrid	[-100, 100]	30	1200
C17F13	Hybrid function 4	Hybrid	[-100, 100]	30	1300
C17F14	Hybrid function 5	Hybrid	[-100, 100]	30	1400
C17F15	Hybrid function 6	Hybrid	[-100, 100]	30	1500
C17F16	Hybrid function 7	Hybrid	[-100, 100]	30	1600
C17F17	Hybrid function 8	Hybrid	[-100, 100]	30	1700
C17F18	Hybrid function 9	Hybrid	[-100, 100]	30	1800
C17F19	Hybrid function 10	Hybrid	[-100, 100]	30	1900
C17F20	Composition function 1	Composition	[-100, 100]	30	2000
C17F21	Composition function 2	Composition	[-100, 100]	30	2100
C17F22	Composition function 3	Composition	[-100, 100]	30	2200
C17F23	Composition function 4	Composition	[-100, 100]	30	2300
C17F24	Composition function 5	Composition	[-100, 100]	30	2400
C17F25	Composition function 6	Composition	[-100, 100]	30	2500
C17F26	Composition function 7	Composition	[-100, 100]	30	2600
C17F27	Composition function 8	Composition	[-100, 100]	30	2700
C17F28	Composition function 9	Composition	[-100, 100]	30	2800
C17F29	Composition function 10	Composition	[-100, 100]	30	2900
C19F1	Storn’s Chebyshev Polynomial Fitting Problem	Multimodal	[-8192, 8192]	9	1
C19F2	Inverse Hilbert Matrix	Multimodal	[-16384, 16384]	16	1
C19F3	Lennard-Jones Minimum Energy Cluster	Multimodal	[-4, 4]	18	1
C19F4	Shifted and rotated Rastrigin’s function	Multimodal	[-100, 100]	10	1
C19F5	Shifted and rotated Griewank’s function	Multimodal	[-100, 100]	10	1
C19F6	Shifted and rotated Weierstrass function	Multimodal	[-100, 100]	10	1
C19F7	Shifted and rotated Schwefel’s function	Multimodal	[-100, 100]	10	1
C19F8	Shifted and rotated Schaffer’s function	Multimodal	[-100, 100]	10	1
C19F9	Shifted and rotated Happy Cat function	Multimodal	[-100, 100]	10	1
TID	Name	Type	Range	Dim	$f_{min}$
C19F10	Shifted and rotated Ackley function	Multimodal	[-100, 100]	10	1
C21F1	Shifted and Rotated Bent Cigar function	Unimodal	[-100, 100]	10	100
C21F2	Shifted and Rotated Schwefel’s function	Basic	[-100, 100]	10	1100
C21F3	Shifted and Rotated Lunacek bi-Rastrigin function	Basic	[-100, 100]	10	700
C21F4	Expanded Rosenbrock’s plus Griewangk’s function	Basic	[-100, 100]	10	1900
C21F5	Hybrid function 1	Hybrid	[-100, 100]	10	1700
C21F6	Hybrid function 2	Hybrid	[-100, 100]	10	1600
C21F7	Hybrid function 3	Hybrid	[-100, 100]	10	2100
C21F8	Composition function 1	Composition	[-100, 100]	10	2200
C21F9	Composition function 2	Composition	[-100, 100]	10	2400
C21F10	Composition function 3	Composition	[-100, 100]	10	2500
C22F1	Shifted and full Rotated Zakharov function	Unimodal	[-100, 100]	10	300
C22F2	Shifted and full Rotated Rosenbrock’s function	Basic	[-100, 100]	10	400
C22F3	Shifted and full Rotated Expanded Schaffer’s F7	Basic	[-100, 100]	10	600
C22F4	Shifted and full Rotated Non-Continuous Rastrigin’s function	Basic	[-100, 100]	10	800
C22F5	Shifted and full Rotated Levy function	Basic	[-100, 100]	10	900
C22F6	Hybrid function 1	Hybrid	[-100, 100]	10	1800
C22F7	Hybrid function 2	Hybrid	[-100, 100]	10	2000
C22F8	Hybrid function 3	Hybrid	[-100, 100]	10	2200
C22F9	Composition function 1	Composition	[-100, 100]	10	2300
C22F10	Composition function 2	Composition	[-100, 100]	10	2400
C22F11	Composition function 3	Composition	[-100, 100]	10	2600
C22F12	Composition function 4	Composition	[-100, 100]	10	2700

### Real-world problems

The following ten RWPs have been used to evaluate the applicability of IFOX in the real-world: bulk carriers problem (BCP), cantilever beam problem (CBP), tension/compression spring design (CSD), car side impact problem (CSP), gear train problem (GTP), pressure vessel design (PVD), piston lever design (PLD), speed reducer problem (SRP), tubular column problem (TCP), and welded beam problem (WBP).

- BCP: Minimizes the cost-to-capacity ratio, lightweight structural weight, and maximizes deadweight capacity by optimizing six bulk carrier design variables (*L*, *B*, *D*, *T*, $V_{k}$, *C*_*B*_), subject to nine nonlinear structural and hydrodynamic constraints.
- CBP: Reduces cantilever beam weight by selecting optimal values for five structural variables, ensuring adequate load-bearing capacity.
- CSD: Minimizes spring weight through optimal selection of wire diameter (*d*), coil diameter (*D*), and number of active coils (*N*), satisfying constraints related to shear stress, surge frequency, and deflection.
- CSP: Optimizes vehicle structural weight, adhering to stringent side-collision safety standards using eleven mixed-type design variables and nonlinear crashworthiness constraints.
- GTP: Determines integer gear teeth numbers (*T*_*a*_, *T*_*b*_, *T*_*d*_, *T*_*f*_ between 12 and 60) to minimize error from a desired gear ratio of 6.931.
- PVD: Minimizes pressure vessel cost using four decision variables: shell thickness (*T*_*s*_), head thickness (*T*_*h*_), vessel radius (*R*), and cylindrical length (*L*).
- PLD: Minimizes oil volume in hydraulic piston mechanisms by optimizing piston components (*H*, *B*, *D*, and *X*) during lever actuation between $0^{\circ }$ and $45^{\circ }$.
- SRP: Minimizes speed reducer mass through seven design variables: face width (*Z*_1_), tooth module (*Z*_2_), number of pinion teeth (*Z*_3_), shaft lengths (*Z*_4_, *Z*_5_), and shaft diameters (*Z*_6_, *Z*_7_), subject to gear and shaft stress constraints.
- TCP: Optimizes tubular column outer diameter (*d*) and thickness (*t*) to minimize material cost, ensuring axial stress and buckling constraints are met.
- WBP: Minimizes welded beam fabrication cost via four variables: weld thickness (*h*), joint length (*l*), beam height (*t*), and beam thickness (*b*), under shear stress, bending stress, buckling, and deflection constraints.

Implementations of these RWPs are publicly accessible at: https://github.com/thieu1995/enoppy [64–68].

### FOX optimization algorithm

FOX is a recent optimization algorithm inspired by the hunting behavior of red foxes, it simulates red foxes’ behavior in the wild, including walking, jumping, searching for food, and hunting prey [10]. It incorporates physics-based principles such as prey detection based on sound and distance, agent jumping governed by gravity and direction, and additional computations such as timing and walking [69]. These features make FOX a competitive optimization algorithm, outperforming several established methods such as PSO, GA, GWO, fitness dependent optimizer (FDO), and WOA [70]. Furthermore, it has been widely adopted in optimization research due to its effectiveness and robustness [71–73].

FOX operates as a population-based algorithm, where multiple search agents, referred to as fox agents (FAs), work independently to find the optimal solution. Each agent has its own solution and fitness value, and they collectively strive to achieve the best fitness value in an iterative manner [10]. However, FA moves within the problem search space; it always thinks of two options, either exploration or exploitation. The probability of choosing exploration and exploitation in the FOX are fixed to 50% for each process. When the FA decides to move, the likelihood of exploration is equal to the probability of exploitation, meaning that FA has a probability of 0.5 for exploration and 0.5 for exploitation [70]. The implementation is available at https://github.com/hardi-mohammed/fox.

#### Exploitation in FOX optimization algorithm.

FAs estimate the distance to their prey based on the time it takes for the ultrasound signal to reach them, given that sound travels at a constant speed of 343 meters per second [74]. This estimation process enables the FAs to determine when to jump to capture their prey. The jump mechanism enhances FOX ’s ability to escape local optima, resulting in superior performance on benchmark test functions and real-world engineering design problems [5,10,75]. Consequently, the FA ’s success in capturing prey is closely tied to its ability to interpret the sound’s travel time while jumping accurately [73]. However, FA exploits through several steps inspired by nature and modeled using mathematical and physical equations.

$$Sp\_S=\frac{BestX}{Time\_ST}$$ (1)

Dist_ST=Sp_S⊙Time_ST (2)

where Dist_ST is the distance of sound travels, Sp_S is the sound speed in the medium that is approximately 343 meter per second, but the FOX calculates modified Sp_S based on the Eq (1), Time_ST is a random time variable in range [0,1] required by the sound wave to travels from the prey to the FA, and *it* is the current epoch [10].

Dist_Fox_Prey=Dist_ST·0.5 (3)

where Dist_Fox_Prey is the distance between the FA and his prey and *BestX* is the global best solution [10].

Initially, the FA use Eq (2) to calculate the distance of sound that travels from the prey by multiplying the random time variable by the modified speed of the sound from Eq (1). Red foxes have large ears compared to their head size, which works as radar to enable them to capture the ultrasound waves reflected from the environment. However, FA uses the principle of ultrasound waves inspired by the Doppler effect to determine the prey’s location as presented in the Eq (3).

$$Jump=0.5\cdot 9.81\cdot t^{2},\;t=\frac{AVG(Time\_ST)}{2}$$ (4)

where *Jump* is the control variable used in the following Eq (5) to determine the new position (solution), *t* is the average time divided by 2, and the approximate value 9.81 is the gravitational constant, ignoring the effects of air resistance, also termed *G* [10].

$$X_{it+1}=Dist\_Fox\_Prey\cdot Jump\cdot \left\{ \begin{array}{cc} c_{1}, & \text{if }p>0.18\\ c_{2}, & \text{otherwise} \end{array}\right.$$ (5)

At this moment, the FA has determined the location of the prey using the Eq (3), and he must decide how he will hunt. Thus, FA has two options: jumping towards the north or the opposite direction using the jump variable from the Eq (4). The method of determining the direction of the jump depends on a random variable called *p* in the range [0,1] and two hyperparameters, *c*_1_ and *c*_2_, where the value of *c*_1_ in range [0,0.18], and the value of *c*_2_ in range [0.19,0.82], which are fixed numbers tuned at the beginning of the optimization process. Eq (5) is used to carry out the process of hunting the prey (find the new solution *X*_*it* + 1_), depend on the variable *p*, which is the probability of using *c*_1_ or *c*_2_ in hunting process [10].

The exploitation process searches locally to refine the best solution by making slight adjustments multiple times. However, if the optimization algorithm relies on exploitation only, it may become stuck in local optima and cannot discover new solutions. Hence, optimization algorithms should use both exploitation and exploration. On the other hand, the exploration process expands the search globally to find new potential solutions. The following section will discuss the FOX ’s exploration phase in detail.

#### Exploration in FOX optimization algorithm.

During the exploration phase, FAs employs a random walk strategy to locate potential solutions, analogous to how red foxes search for prey. They utilize their ability to detect ultrasound signals to assist in the search process of locating prey [71]. The method of searching is facilitated through controlled random walks, ensuring the FA progresses toward the prey while maintaining an element of randomness. During this phase, FOX ’s unique random walk and distance measurement mechanisms enable a refined search process, distinguishing it from other established swarm-based algorithms like PSO and GWO [73].

$$a=2\cdot (it-(\frac{1}{Max}))$$ (6)

$$Mint=Min(tt),\;tt=\frac{\sum Time\_ST}{dim}$$ (7)

$$X_{it+1}=BestX⊙rnd(1,dim)\cdot Mint\cdot a$$ (8)

where *rnd*(1,*dim*) is a uniform random value used to provide slight perturbation for the solution to enhance the diversity, and *dim* is the problem [10].

As mentioned, the FA explore the problem search space by searching for the best solution by random walk strategy. The Eq (8) used for exploration to search for new position (*X*_*it* + 1_) for the current FA. Additionally, it has two adaptive hyperparameters: *a* is calculated based on the FOX epochs as seen in Eq (6) and *Mint* is calculated based on the minimum averaged time as seen in the Eq (7).

**Fig 1:**
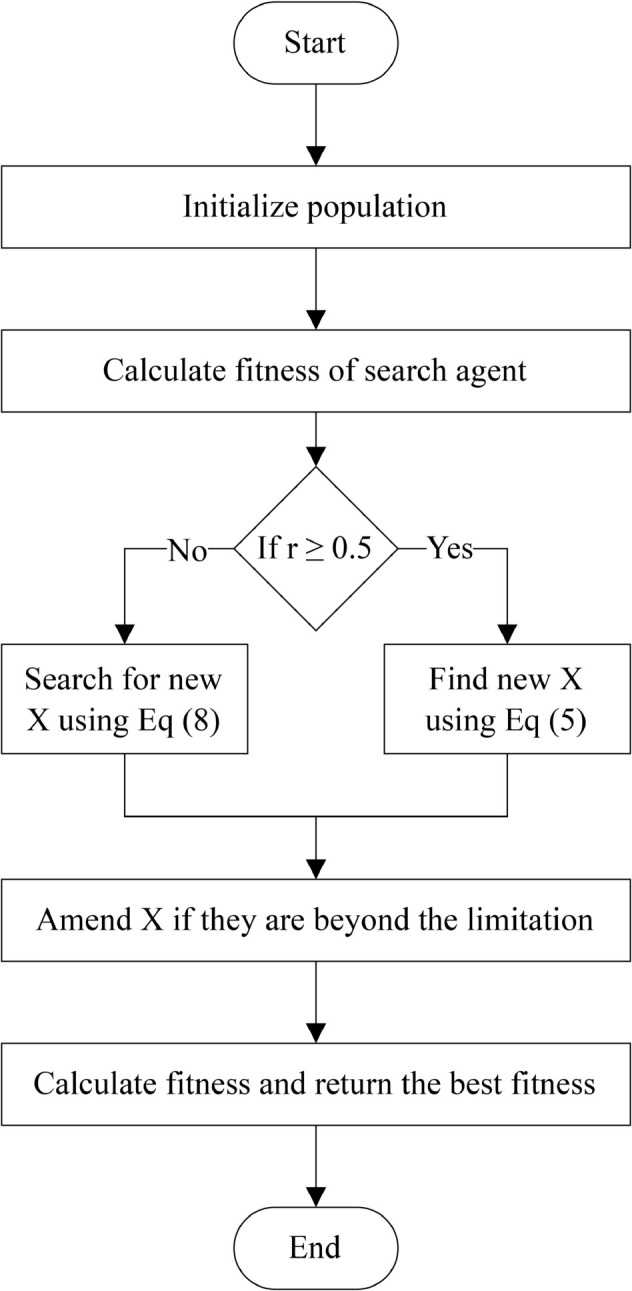
Flowchart of a single epoch in the FOX optimization algorithm [10].

After each epoch of optimization (whether exploration or exploitation), the FA evaluates the objective function value (fitness) based on the current solution. If this solution leads to better convergence, it is considered the best solution. Figure 1 visualizes the primary steps of FOX redrawn from the original study [10].

#### Limitations in FOX optimization algorithm.

**Fig 2:**
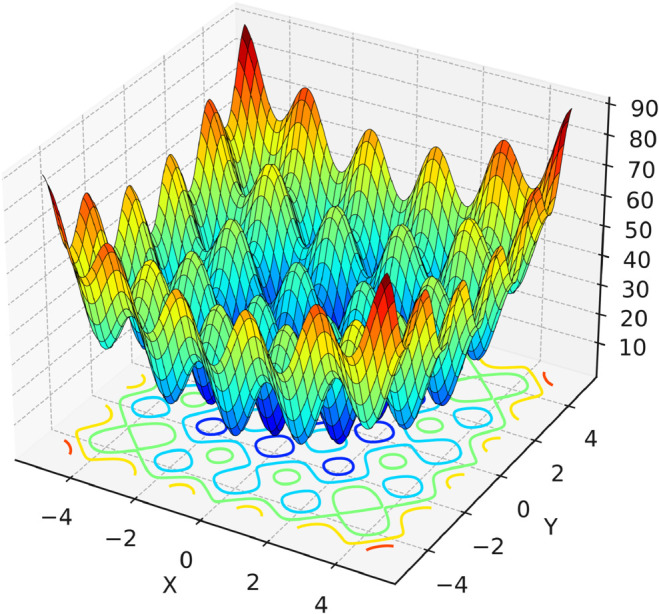
3D visualization of the Rastrigin function from the CEC 2019 benchmark test functions. It contains a large number of local optima. The optimization in such a function (landscape) requires a balance between exploration to avoid local optima and exploitation to converge accurately near the global optimum. It is important to use adaptive methods to balance exploration and exploitation in such situations. Adopted from [11].

Although the FOX is superior to several established optimization algorithms, it has notable limitations, as observed through experiments conducted in this paper across various tests. Furthermore, the authors of the FOX acknowledged these limitations, stating: “*The FOX exploration phase can be enhanced to be more competitive against other algorithms. The exploitation phase of the FOX also can be improved by changing the equations used to find the distance of the red Fox from prey and the jump equation. The convergence of the fitness value can be improved based on changing exploration and exploitation equations*” [10]. Additionally, the method of exploitation and exploration in the FOX is static, with each being set to 0.5. This does not match the nature of many optimization problems, as some issues need more exploration, and others need more exploitation. For instance, the search space has many local optima in a complex benchmark test function C19F4, as visualized in Fig 2. Therefore, more exploration is required in the early stages. However, with the fixed 0.5 value, the algorithm may start exploiting too early and become trapped in local optima. Conversely, in simple problems, excessive exploration may result in inefficient convergence. This shows that using a fixed value limits the algorithm’s ability to adapt to the nature of the problem.

### Improved FOX optimization algorithm

Several significant improvements have been made to the FOX. Address limitations, contribute to higher performance, and reduce the restriction the FOX faces. Consequently, a new adaptive method for balancing exploration and exploitation will be discussed in Adaptive exploration and exploitation section, modifying certain equations and rewriting others for better clarity. Furthermore, the complete implementation is available at https://github.com/mwdx93/ifox.

#### Exploitation in the improved FOX optimization algorithm.

Some original equations in FOX are modified throughout the exploitation phase due to their limitations and ambiguity, particularly in time and distance computations. The reasons for these modification such as reducing the number of equations and achieving higher performance with the IFOX, are discussed below. Additionally, the FOX ’s equations are slightly improved, but not entirely, to preserve their general structure and main idea, maintaining their superiority.

Dist_Fox_Prey=BestXTime_ST·Time_ST·0.5 (9)

Dist_Fox_Prey=BestX·0.5 (10)

Eq (9) represents the direct substitution of Eq (1) and (2) into Eq (3). When expanded, it becomes evident that the time variable Time_ST cancels out, leading to a redundant form where the distance to prey becomes directly proportional to the best solution *BestX*, scaled by a constant factor. This cancellation introduces ambiguity in the FOX exploitation mechanism, as the Time_ST variable initially intended to simulate prey detection based on sound travel time becomes mathematically ineffective.

To resolve this ambiguity, Eq (10) is introduced in the IFOX. It simplifies the computation by directly defining the distance between the fox agent and the prey as half of the best solution *BestX*. This modification eliminates the unnecessary dependence on the canceled Time_ST variable, resulting in a clearer, more consistent mathematical model. Additionally, it maintains the physical intuition behind the exploitation phase while enhancing computational efficiency and clarity. Thus, the transition from Eq (9) to Eq (10) represents a meaningful refinement in the IFOX design. Additionally, in the original FOX, the jump in Eq (4) is computed dynamically during each epoch based on the time variable and the gravitational constant. In contrast, in the IFOX, half of the gravitational constant value is precomputed and defined as a constant outside the main optimization loop. This refinement enhances computational clarity, reduces redundancy, and improves the overall readability of the optimization process.

The IFOX utilized a new adaptive value, *alpha α*. It plays a crucial role in most of the IFOX’s steps because of its impact on the improved exploration and exploitation equations. It must be updated adaptively at each epoch using Eq (11). However, the alpha *α* value has been employed to enhance the diversity and potentially enhance solutions for improved performance. Similarly, the alpha *α* value is also responsible for computing a random uniform value, denoted as *beta β*, which will be used in the modified exploration and exploitation equations to improve them further. The following equations describe how to compute the alpha *α* value in the primary loop and beta *β* vector in the FA loop, respectively:

$$\alpha =\alpha_{min}+(1-\alpha_{min})\cdot (1-\frac{it}{epochs})$$ (11)

$$\alpha_{min}=\frac{1}{0.5\cdot epochs}$$

$$\beta =\left\{ \begin{array}{cc} \text{LevyFlight}(dim)\cdot \alpha , & \text{if rnd}(0,1)<\alpha \\ \text{rnd}(-\alpha ,\alpha ,dim), & \text{otherwise} \end{array}\right.$$ (12)

where $\alpha_{min}$ denotes the empirically chosen lower bound on, ensuring a minimal nonzero perturbation throughout the optimization process. In the IFOX, the *β* vector is adaptively generated based on *α*. Specifically, if a random number in [0,1] is less than *α*, the *β* is computed using a Lévy flight distribution, random walk with mostly small steps and occasional large jumps [76], scaled by *α*; otherwise, it is drawn uniformly from the range [−α,α] for each dimension.

Eq (12) is employed to compute a crucial vector in IFOX, which is generated adaptively at each epoch within the FA loop and subsequently used in the improved exploitation equation:

Xt=Dist_Fox_Prey⊙β·Jump (13)

In the original FOX, the exploitation step is computed based on two fixed quantities: the distance between the fox and its prey from the Eq (3) and the jump value from Eq (4). These values remain constant for each FA during an epoch and are updated only once per main loop epoch. Additionally, the constants *c*_1_ and *c*_2_ used in the movement decision process are static and do not vary. This static behavior limits the diversity of solutions and increases the risk of premature convergence. In contrast, Eq (13) introduces adaptivity by incorporating the*β* vector, which is recalculated for each agent in every epoch. The dynamic nature of *β* significantly enhances solution diversity during the exploitation, leading to better search capabilities and faster convergence.

#### Exploration in the improved FOX optimization algorithm.

The analysis of the FOX’s exploration solution produced by Eq (8) revealed the need for improvements. The exploration process should generate solutions with acceptable diversity; however, the FOX exploration produces solutions with massive variations. For instance, when testing the FOX using a classical function, the best solution is BestX=[0.41,−2.3], while *BestX*_*it* + 1_ = *[*12.7,−9.5*]*, which is significantly different. Hence, the IFOX replaces the multiplication process with an addition process, ensuring a balance by generating solutions that exhibit sufficient—but not excessive—diversity. Furthermore, the variables *a* and *Mint* have been replaced with the proposed alpha *α* and beta *β*, respectively. These changes lead to the production of exploration solutions *Xr* that outperform those generated the FOX, as presented in the improved equation below:

Xr=BestX+β·α (14)

**Fig 3:**
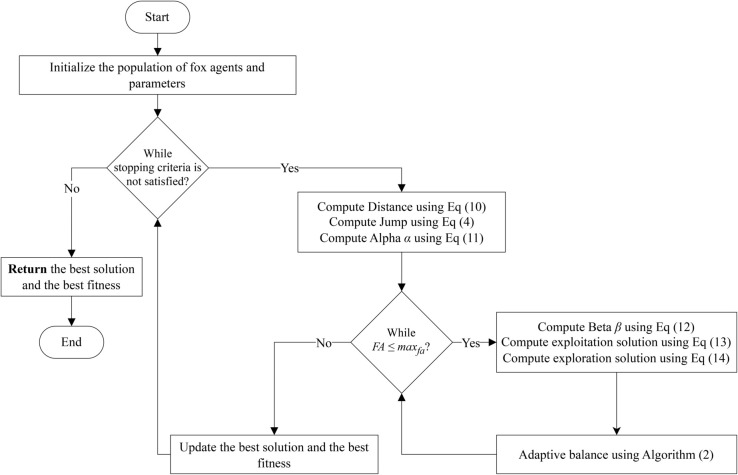
Flowchart of improved FOX optimization algorithm.

#### Adaptive exploration and exploitation.

**Fig 4:**
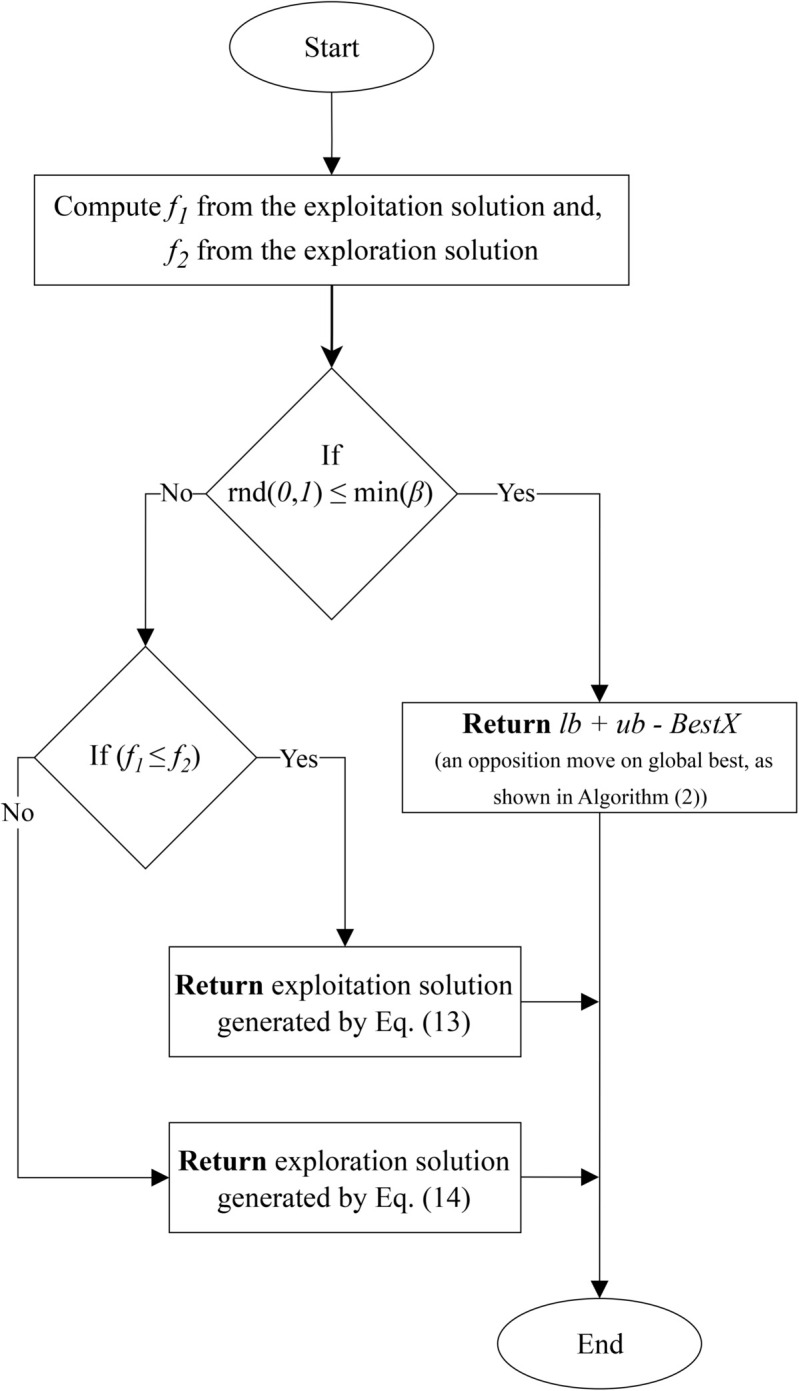
Adaptive exploration and exploitation method of Improved FOX optimization algorithm.

This section explains how the method of balancing exploration and exploitation is transformed into an adaptive approach in the IFOX, based on the fitness values of the problem solutions inspired by the study in [77]. The FOX uses a random variable, *r*, which ranges between 0 and 1, to determine whether the algorithm will perform exploration or exploitation. Specifically, if r≥0.5, the FA performs exploitation; otherwise, it performs exploration. In contrast, the IFOX eliminates the random *r* variable and instead uses the fitness values of candidate solutions, as visualized in Fig 4. The proposed method adaptively selects exploration or exploitation based on the solution’s fitness value while incorporating a small probability of performing an opposition-based move.

In Algorithm 2, after computing the fitness values *f*_1_ and *f*_2_, a random number in [0,1] is generated and compared to min(β). Suppose the random number is less than or equal to the minimum of beta *β*. In that case, the FA performs an opposition move by replacing the current solution with an opposite position relative to the global best. This adaptive strategy introduces controlled randomness into the search process: when *β* is large, more opposition moves occur to promote exploration; when *β* is small, the search process favors exploitation. This mechanism further improves the IFOX’s ability to escape local optima and enhances overall convergence behavior. However, if the opposition move is not triggered, the IFOX compares the two fitness values, *f*_1_ and *f*_2_, obtained by evaluating the exploitation solution from Eq (13) and exploration solution from Eq (14), respectively. If *f*_1_ is less than *f*_2_ (for minimization problems), the solution from the exploitation phase is selected for the current FA; otherwise, the exploration solution is chosen. This adaptive decision-making process enhances the algorithm’s ability to balance exploration and exploitation more effectively than the static balancing method used in the FOX, leading to faster convergence and higher-quality solutions.

**Algorithm 1. Main loop of the Improved FOX optimization algorithm.**

Initialize $max\_iter,max_{fa},Jump,BestX,BestFitness$

Initialize the fox agents population $X=\{X_{fa}|fa=1,2,\ldots ,max_{fa}\}$

**constant** half_G←4.905

**while** it<max_iter **do**

Compute the distance     ⊳ Eq (10)

$Jump\leftarrow half\_G\times t^{2}$     ⊳ Rewritten Eq (4)

Compute alpha *α*     ⊳ Eq (11)

**for** *fa* = 0 to *max*_*fa*_ **do**

Compute beta *β*     ⊳ Eq (12)

Compute exploitation solution *X*_*t*_     ⊳ Eq (13)

Compute exploration solution *X*_*r*_     ⊳ Eq (14)

$X_{fa}\leftarrow$ Balance(*BestX*, *X*_*t*_, *X*_*r*_, *β*)     ⊳ Algorithm 2

**end for**

**for** *fa* = 0 to *max*_*fa*_ **do**

$f\leftarrow Fitness(X_{fa})$

**if** f≤BestFitness **then**

BestFitness←f

$BestX\leftarrow X_{fa}$

**end if**

**end for**

it←it+1

**end while**

**return** *BestX*, *BestFitness*

## Results

Experimental evaluations were carried out to assess the performance of the proposed IFOX. Its results were compared against several recently improved and SOTA optimization algorithms discussed in the Literature review, including ALSHADE, BBOA, BDA, LEO, CMAES, COA, CPO, FOX, HO, IAGA, SHOA, IPSO, IWOA, LSHADE, NRO, and VWGWO. They were implemented in Python and executed on an MSI GL63 8RD laptop, equipped with an Intel Core i7-8750H CPU (12 threads) and 32GB of RAM. The experimental settings—including the number of epochs, population size, and trials—were standardized to ensure consistency, as detailed in Table 3. The evaluation employed a comprehensive set of 81 benchmark test functions and ten RWPs, with each experiment independently executed 30 times under identical conditions. Results were aggregated by averaging to ensure the robustness and reliability of the performance assessment across diverse optimization tests.

**Table 3:** General parameters setting.

Parameter	Value
No. of epochs	500
No. of populations	30
No. of trials	30
Trials aggregation type	Average

The performance metrics, including the average convergence (Avg), standard deviation (Std), best convergence (Best), worst convergence (Worst), Bayesian rank-sum test (BRT), and Rank have been used for evaluations. Avg represents the mean best objective value (fitness) over 30 independent runs, indicating the optimization efficiency. Std measures the stability across runs, with lower values implying higher robustness. Best and Worst denote the minimum and maximum objective values achieved, respectively. The BRT provides posterior probabilities of one algorithm outperforming another, addressing issues in multiple comparisons. It counts wins/ties/losses (+/=/-) for each optimization algorithm across the optimization experiment. A win count means better performance than other algorithms, a tie count means equivalent performance, and a loss count means worse performance. The Rank is computed using the Friedman statistical test. It defines the ranking of each optimization algorithm based on the Avg metric across all tested tasks. The lower rank values indicate better overall performance. Furthermore, the processing time (PT) is the average execution time the optimization algorithm takes per epoch.

**Algorithm 2. Adaptive method for exploration and exploitation.**

**function** Balance*BestX*, *X*_*t*_, *X*_*r*_, *β*

$f_{1}\leftarrow Fitness(X_{t})$

$f_{2}\leftarrow Fitness(X_{r})$

**if** rnd(0,1)≤min(β) **then**

**return** *lb* + *ub*−*BestX* ⊳ *lb* and *ub* are the problem’s bounds.

**else**

**if** $f_{1}\leq f_{2}$ **then**

**return** *X*_*t*_

**else**

**return** *X*_*r*_

**end if**

**end if**

**end function**

Table 4 provides the comprehensive performance results among 17 recent, improved, SOTA optimization algorithms, including the IFOX. The results demonstrate that IFOX achieved outstanding performance across various benchmark test functions, consistently ensuring top rankings on several functions like: CL1, CL5, CL8, CL10, CL11, CL13, CL15, CL17, C17F1, C17F2, C17F16, C17F20, C19F1, C19F2, C19F6, C21F9, C22F1, and C22F11. Moreover, in RWPs, IFOX showed strong performance on the CBP and GTP. Total results indicate that IFOX achieved 880 wins, 228 ties, and 348 losses across all experiments. It also maintained a promising mean rank of 5.92 and demonstrated computational performance with an average PT of 0.0038 seconds. Finally, these findings emphasize the competitiveness and effectiveness of the proposed IFOX, making it comparable or superior to SOTA and recent optimization algorithms such as ALSHADE, LSHADE, CMA-ES, and NRO.

**Table 4:**
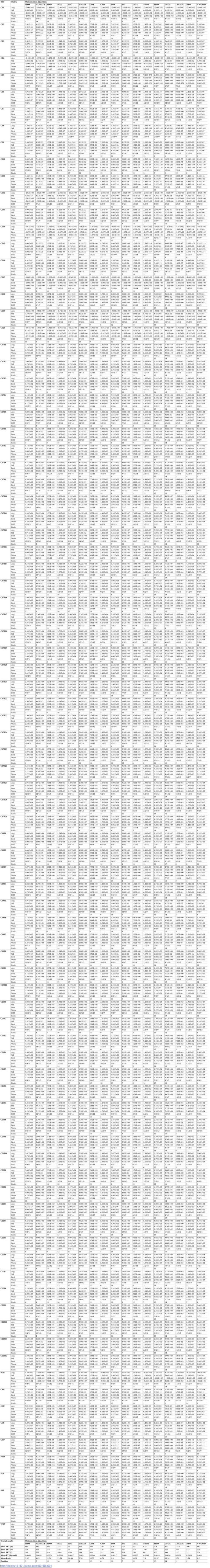
The performance results on benchmark test functions and real-world problems.

### Convergence results

Convergence in optimization refers to an algorithm’s capability to progressively approach the optimal or near-optimal solution over epochs. The following figures illustrate the convergence performance of IFOX (red) compared to other optimization algorithms that are considered in this paper. Each figure displays selected functions where significant differences in convergence are observed, whereas functions showing similar convergence patterns across all optimization algorithms have been omitted here and are supplied in the Supporting Information file S1 File.

**Fig 5:**
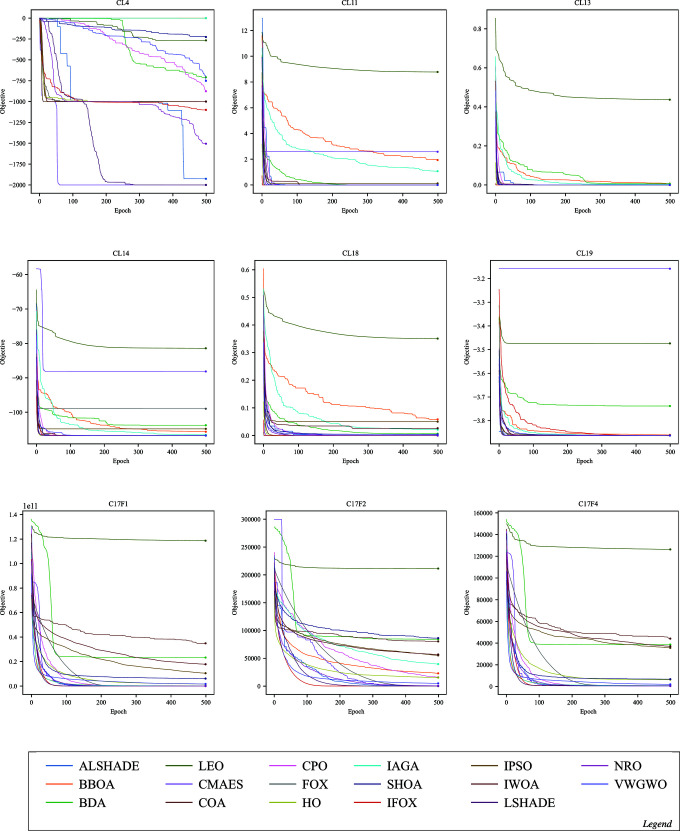
Convergence curves on the functions CL4, CL11, CL13, CL14, CL18, CL19, C17F1, C17F2, and C17F4.

**Fig 6:**
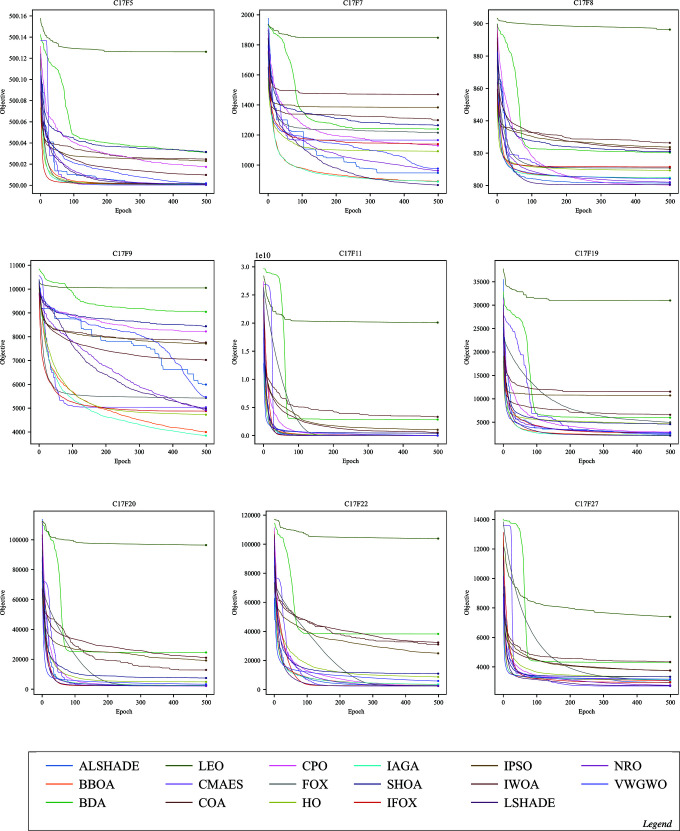
Convergence curves on the functions C17F5, C17F7, C17F8, C17F9, C17F11, C17F19, C17F20, C17F22, and C17F27.

**Fig 7:**
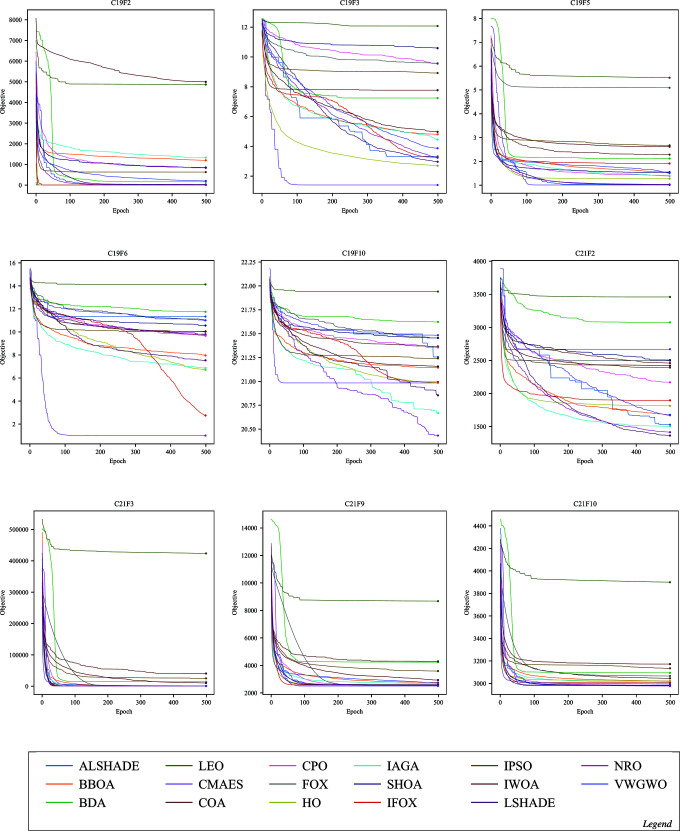
Convergence curves on the functions C19F2, C19F3, C19F5, C19F6, C19F10, C21F2, C21F3, C21F9, and C21F10.

**Fig 8:**
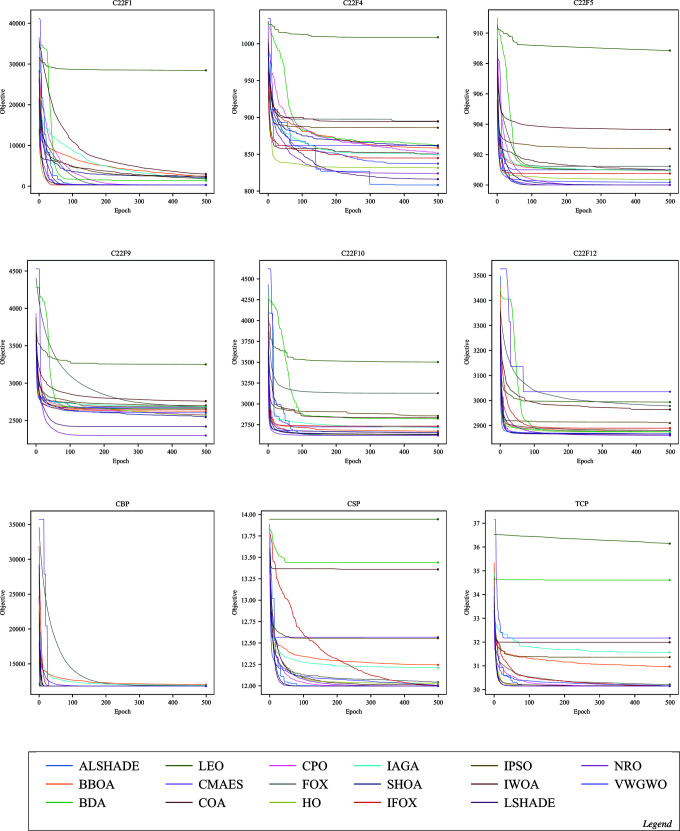
Convergence curves on the functions C22F1, C22F4, C22F5, C22F9, C22F10, C22F12, CBP, CSP, and TCP.

The convergence performance of IFOX in Figs 5, 6, 7, and 8, showed rapid convergence on functions CL11, CL13, CL14, and CL18, outperforming the competing optimization algorithms during the initial epochs (the first ten). Furthermore, it consistently outperformed other algorithms within the first 100 epochs on C17F1, C17F2, C17F4, C17F5, C17F20, C21F9, C21F3, C19F2, and C22F1, reflecting unique convergence. In contrast, for functions C17F7, C17F8, C17F9, C17F11, C17F19, C21F10, C22F9, and C22F10, it demonstrated convergence performance comparable to other algorithms, showing neither a distinct advantage nor significant delayed. For another group of functions, such as CL4, CL19, C19F3, C19F6, C19F5, C19F10, C21F2, C22F4, and C22F5, the convergence performance of it was relatively weaker, without clear superiority, despite achieving high rankings in terms of final optimal values, as observed in C19F6. Additionally, it demonstrated convergence patterns similar to competing algorithms in real-world optimization problems, except for the CBP, CSP, and TSP problems, where its convergence was less effective, particularly noticeable in the CSP problem. The above convergence examination shows that IFOX exhibits robust convergence performance on several experiments, particularly during early epochs. However, it performed comparably or somewhat weaker on other functions and RWPs, making it a good avenue for future improvement. Moreover, further interpretation and analysis of results are required to identify the strengths and limitations of the IFOX. Hence, an in-depth analysis will be discussed in the next section.

## Discussion

This section analyzes and discusses the performance of IFOX in comparison with other optimization algorithms. While the results indicate that IFOX is generally effective in solving optimization test functions and problems, achieving optimal solutions alone is not sufficient, as many algorithms are capable of this. Therefore, it is essential to examine and compare the convergence behavior, particularly the average convergence metrics, to highlight the advantages of IFOX and justify the proposed contributions. As shown in Table 4, IFOX outperformed most competing algorithms across 15 benchmark test functions and two RWPs. To ensure a rigorous comparison, statistical significance was assessed using the non-parametric Friedman and Wilcoxon signed-rank tests [78,79]. A p-value below 0.05 was considered statistically significant in this analysis.

The IFOX convergence curves (red) in Figs 5–8 demonstrate that the algorithm rapidly achieves optimal solutions on CL11, CL13, C17F1, and C17F2 functions before its competitors during initial epochs. The fast exploration and exploitation capabilities arise from the proposed parameters alpha *α* and beta *β* in Eqs (11) and (12) that utilized in the proposed adaptive balance strategy. IFOX converged similarly to other algorithms on functions such as C17F7 and C22F9 because the problem domains did not give it an advantage during exploration. The convergence time was slower on C19F6 and C22F4 because these functions exhibited short periods of stability and restricted early solution exploration, but IFOX proved able to discover efficient final solutions. The performance consistency in RWPs showed exceptions only in CBP, CSP, and TCP because these problems had complex constraints that reduced the effectiveness of IFOX. This evidence reveals that IFOX functions optimally in smooth continuous domains; however, it still lacks enhancement for highly constrained and discrete problem types.

**Fig 9:**
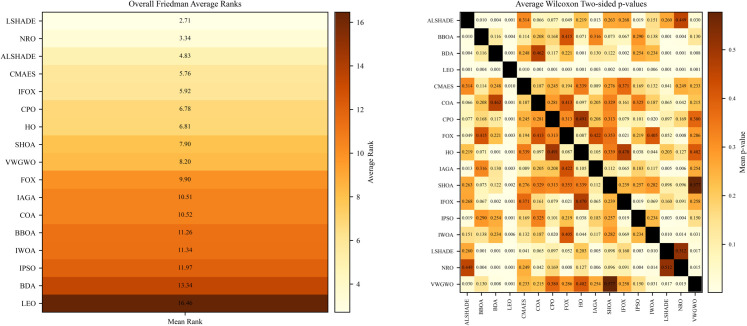
Average Friedman test ranks (left) and pairwise average Wilcoxon signed-rank test p-values (right) for all compared optimization algorithms across CL, C17, C19, C21, C22, and RWPs.

The statistical analysis from Table 5 showed that IFOX was superior to BBOA, BDA, LEO, COA, CPO, FOX, IAGA, and VWGWO on the CL benchmark test functions. It exhibited no statistically significant difference when compared to ALSHADE, CMA-ES, HO, SHOA, IPSO, and IWOA, but was significantly outperformed by NRO and LSHADE. On the C17 functions, IFOX outperformed all optimization algorithms except ALSHADE, CMA-ES, and NRO, where it showed comparable performance, while it was significantly defeated by LSHADE. Moreover, for the C19 functions, IFOX was superior to BDA, LEO, COA, FOX, IPSO, IWOA, and VWGWO. It performed statistically similar to ALSHADE, CPO, HO, IAGA, SHOA, LSHADE, and NRO, but was significantly outperformed by CMA-ES. Additionally, in the C21 functions, IFOX outperformed BBOA, BDA, LEO, COA, FOX, SHOA, IPSO, and IWOA, and showed no significant difference compared to CPO, HO, IAGA, and VWGWO. However, it was significantly defeated by ALSHADE, CMA-ES, LSHADE, and NRO. Furthermore, on C22, IFOX demonstrated superiority over BDA, LEO, COA, IPSO, and IWOA, and showed no significant difference with BBOA, CMA-ES, FOX, HO, IAGA, SHOA, and VWGWO, while it was significantly outperformed by ALSHADE, CPO, LSHADE, and NRO. Finally, on the RWPs, IFOX significantly outperformed BBOA, BDA, LEO, CMA-ES, FOX, IAGA, IPSO, and IWOA, and exhibited statistically similar performance with ALSHADE, COA, HO, SHOA, LSHADE, and VWGWO, but was significantly defeated by CPO and NRO. These analyses are further summarized by the pairwise comparisons in the right-hand side of Fig 9, which displays the average Wilcoxon signed-rank test p-values. The darker cells in the heatmap reflect non-significant differences, whereas lighter cells, especially those near the 0.05 p-value, demonstrate statistically significant differences between the tested algorithms. Additional analyses—including algorithm-vs-algorithm comparisons (e.g., LSHADE vs. other algorithms, NRO vs. other algorithms), pairwise p-value tables and figures for each benchmark group, and ranking figures for each group—are provided in the Supporting Information file S1 File.

**Table 5:** Wilcoxon signed-rank test results comparing IFOX with other optimization algorithms across CL, C17, C19, C21, C22, and RWPs. Values include positive ranks (R^ + ^), negative ranks (R^−^), and two-sided p-values indicating statistical significance

Wilcoxon signed-rank test of IFOX versus optimization algorithms																		
Algorithm	CL			C17			C19			C21			C22			RWPs		
	R^ + ^	R^−^	P-val	R^ + ^	R^−^	P-val	R^ + ^	R^−^	P-val	R^ + ^	R^−^	P-val	R^ + ^	R^−^	P-val	R^ + ^	R^−^	P-val
ALSHADE	59	112	0.25	150	285	0.15	22.0	23.0	1.00	5	50	0.02	11	67	0.03	10	35	0.16
BBOA	210	0	0.00	382	53	0.00	44.0	11.0	0.11	49	6	0.03	54	24	0.27	55	0	0.00
BDA	153	0	0.00	435	0	0.00	55.0	0.0	0.00	55	0	0.00	74	4	0.00	45	0	0.00
LEO	190	0	0.00	435	0	0.00	55.0	0.0	0.00	55	0	0.00	78	0	0.00	45	0	0.00
CMAES	72	64	0.84	200	235	0.72	0.0	45.0	0.00	7	48	0.04	32	46	0.62	44	1	0.01
COA	75	3	0.00	435	0	0.00	45.0	0.0	0.00	55	0	0.00	65	13	0.04	21	24	0.91
CPO	111	25	0.03	399	36	0.00	43.0	12.0	0.13	39	16	0.28	12	66	0.03	1	44	0.01
FOX	66	0	0.00	429	6	0.00	45.0	0.0	0.00	55	0	0.00	62	16	0.08	48	7	0.04
HO	51	27	0.35	424	11	0.00	24.0	21.0	0.91	39	16	0.28	29	49	0.47	20	25	0.82
IAGA	210	0	0.00	383	52	0.00	42.0	13.0	0.16	47	8	0.05	57	21	0.18	55	0	0.00
SHOA	80	56	0.53	430	5	0.00	43.0	12.0	0.13	50	5	0.02	45	33	0.68	7	38	0.07
IPSO	111	42	0.10	435	0	0.00	55.0	0.0	0.00	55	0	0.00	78	0	0.00	44	1	0.01
IWOA	56	97	0.33	435	0	0.00	55.0	0.0	0.00	48	7	0.04	66	12	0.03	44	1	0.01
LSHADE	24	96	0.04	49	386	0.00	24.0	31.0	0.77	5	50	0.02	0	78	0.00	9	36	0.13
NRO	19	86	0.04	163	272	0.25	12.0	33.0	0.25	0	55	0.00	0	78	0.00	3	52	0.01
VWGWO	120	0	0.00	409	26	0.00	45.0	10.0	0.08	43	12	0.13	28	50	0.42	24	21	0.91

As reported in Table 6 and illustrated in the left-hand side of Fig 9, IFOX classified 5th overall according to the non-parametric analysis using the Friedman test, with an average rank of 5.92. This rank is 118% behind the LSHADE (the top algorithm), 77% after the NRO (second), 22% behind the ALSHADE (third), and 2% behind the CMA-ES (fourth). Achieving a rank close to these top performers—and substantially better than the original FOX, with over 40% improvement in ranking—indicates that the IFOX has successfully produced an enhanced version of the original algorithm. Although the average rank of IFOX is 118% higher than LSHADE, it still ranks within the top five across 17 optimization algorithms that show a competitive performance. This improvement is primarily attributed to the proposed modifications, including the proposed parameters alpha *α* and beta *β*, which play a crucial role in the IFOX. Furthermore, the modification of key FOX’s exploration and exploitation equations, Eqs (13) and (14). In addition to the replacing of static balance method of FOX with the proposed adaptive method in Algorithm 2. These modifications collectively contribute to producing a competitive optimization algorithm. Regardless, the IFOX still has limitations, which are discussed in the following section.

**Table 6:** Friedman test average rankings of IFOX and competing optimization algorithms across CL, C17, C19, C21, C22, and RWPs. The “Position” column indicates the overall rank based on the average performance.

Algorithm	Friedman test ranking/Functions						Mean	Position
	CL	C17	C19	C21	C22	RWPs		
LSHADE	2.75	1.90	3.70	3.30	1.92	2.70	2.71	1
NRO	2.55	4.14	3.50	2.80	2.25	4.80	3.34	2
ALSHADE	7.00	4.45	6.20	3.40	4.25	3.70	4.83	3
CMAES	6.75	4.48	1.30	2.70	6.42	12.90	5.76	4
**IFOX**	**4.65**	**4.72**	**5.30**	**6.50**	**7.83**	**6.50**	**5.92**	**5**
CPO	7.55	7.38	8.40	6.90	5.67	4.80	6.78	6
HO	4.55	9.45	5.90	8.00	6.83	6.10	6.81	7
SHOA	5.70	11.79	9.90	8.40	7.50	4.10	7.90	8
VWGWO	8.00	8.76	10.20	8.00	7.92	6.30	8.20	9
FOX	6.75	7.72	10.70	11.10	12.25	10.90	9.90	10
IAGA	14.55	7.38	8.80	9.70	10.33	12.30	10.51	11
COA	5.95	14.41	11.30	13.40	11.75	6.30	10.52	12
BBOA	15.65	7.45	10.20	11.00	10.67	12.60	11.26	13
IWOA	4.80	13.41	12.40	12.30	13.75	11.40	11.34	14
IPSO	7.00	13.90	12.80	13.90	13.92	10.30	11.97	15
BDA	11.05	14.69	13.10	14.70	12.50	14.00	13.34	16
LEO	15.80	16.97	16.90	16.90	16.92	15.30	16.46	17

### Limitations and scalability analysis

Identifying the limitations of the proposed IFOX was crucial to pinpoint its strengths and weaknesses. To this end, we selected test function C17F8—on which neither IFOX nor competing optimizers reached the optimal objective value 800—and ran it at four problem dimensions (10, 30, 50, 100) and five epoch settings (50, 100, 250, 500, 1000). The results have been averaged over 30 independent runs as presented in Table 7.

**Table 7:** Scalability of the proposed IFOX on C17F8 with the following metrics: Runtime (seconds), Memory usage (MB), and Relative error across problem dimensions (10, 30, 50, 100) and epochs (50, 100, 250, 500, 1000), averaged over 30 independent runs.

Dim	Epoch	Runtime (seconds)	Memory usage (MB)	Relative error
**10**	50	0.304	168.865	0.002
	100	0.611	169.051	0.002
	250	1.549	169.517	0.002
	500	3.065	170.181	0.002
	1000	6.185	172.165	0.002
**30**	50	0.325	172.214	0.019
	100	0.642	172.254	0.014
	250	1.599	172.230	0.012
	500	3.234	172.261	0.014
	1000	6.360	172.692	0.011
**50**	50	0.330	172.663	0.063
	100	0.657	172.677	0.043
	250	1.642	172.677	0.043
	500	3.294	172.709	0.038
	1000	6.543	173.274	0.039
**100**	50	0.370	173.344	0.194
	100	0.727	173.358	0.133
	250	1.814	173.304	0.110
	500	3.656	173.337	0.116
	1000	7.227	174.348	0.118

**Fig 10:**
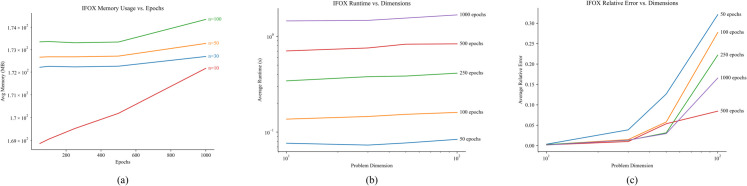
Scalability of IFOX on C17F8: (a) average memory usage, (b) average runtime, and (c) average relative error across epochs (50–1000) for dimensions 10, 30, 50, and 100.

As shown in Fig 10(a), memory consumption increases only slightly with both epochs and dimension: at dimension 10, memory usage rises from 168.9 MB at 50 epochs to 172.2 MB at 1000 epochs (1.9%), while at 1000 epochs scaling from dimension 10 to 100 yields an additional 2.2 MB (1.3%). On the other hand, runtime scales nearly linearly with epochs as shown in Fig 10(b): at dimension 10, average runtime grows from 0.30 s to 6.19 s (20×) when increasing epochs from 50 to 1000; similarly, at 1000 epochs, increasing the dimension from 10 to 100 raises runtime by 17% (6.19 s to 7.23 s). Lastly, the relative error remains very low for low-dimensional cases, decreasing from 0.00233 at 50 epochs to 0.00174 at 500 epochs for dimension 10, but exhibits significant degradation at higher dimensions as shown in Fig 10(c). For dimension 100, relative error peaks at 0.1936 for 50 epochs and plateaus near 0.1178 even at 1000 epochs, which indicates slower convergence.

The scalability analysis demonstrates that IFOX keeps the same trends for memory use and running duration when facing increases in problem dimensions which demonstrates the notable advantages of this algorithm. IFOX faces a major limitation due to its increased relative error in finding the optimal objective value between low and high-dimensional problems. Thus, future investigation needs to develop methods for adaptive epoch control according to problem dimensions and elastic search strategies to boost convergence results in big-dimensional problems. Furthermore, utilizing techniques such as quantum computing and parallel processing with IFOX can potentially lead to improved overall performance.

## Conclusion

In conclusion, this paper introduces IFOX as a new improved optimization algorithm which surpasses FOX through its multiple improvements. Unlike the static balance in FOX, IFOX suggests an adaptive method to balance exploration and exploitation dynamically. It also reduces the number of hyperparameters and modifies the primary equations to enhance performance and address existing limitations. The performance of IFOX has been evaluated using 82 benchmark test functions and ten real-world problems. The results show that IFOX has 880 wins, 228 ties, and 348 losses against the established optimization algorithms across all tested functions and problems. This substantial improvement highlights the effectiveness of the IFOX in solving diverse optimization tasks and real-world problems. However, IFOX also presents certain limitations. Scalability analysis revealed that while memory usage and runtime increase moderately with higher dimensions and epochs, the relative error in high-dimensional problems remains a challenge, indicating a slower convergence rate. Future work should focus on incorporating adaptive epoch control mechanisms based on problem dimensionality and integrating elastic search strategies to mitigate performance degradation in high-dimensional spaces. Moreover, leveraging advanced computational techniques such as parallel processing or quantum computing may further enhance the scalability and applicability of IFOX to real-time and large-scale optimization problems.

## Supporting information

S1 File Appendix A. This appendix includes all convergence plots, as well as the figures and tables for the non-parametric statistical analyses of the conducted experiments.
(PDF)

S2 File Raw results. This compressed file contains all the results of the conducted experiments. Each main directory within the archive includes three subdirectories: Analysis, best_fit, and convergence. The best_fit and convergence folders contain the raw experimental data obtained from 30 independent runs. The Analysis folder includes tables and figures derived from these raw results. These files can be used to reproduce and further analyze the results presented in the manuscript.
(ZIP)

## Abbreviations

FOX: FOX optimization algorithm
IFOX: improved FOX
CEC: congress on evolutionary computation
ACO: ant colony optimization
SHOA: shrike optimization algorithm
TSP: traveling salesman problem
LEO: lagrange elementary optimization
BA: bat algorithm
BBOA: brown-bear optimization algorithm
DA: dragonfly algorithm
BDA: binary dragonfly algorithm
FS: feature selection
GA: genetic algorithms
IAGA: improved adaptive genetic algorithm
GWO: grey wolf optimization
VWGWO: variable weights grey Wolf optimization
ALO: ant lion optimization
PSO: particle swarm optimization
IPSO: improved particle swarm optimization
NRO: nuclear reaction optimization
AGV: automated guided vehicles
FDO: fitness dependent optimizer
WOA: whale optimization algorithm
IWOA: improved whale optimization algorithm
DE: differential evolution
FA: fox agent
TID: optimization task identifier
PT: processing time
ANA: ant nesting algorithm
LPB: learner performance-based behavior algorithm
ABC: artificial bee colony algorithms
SOTA: state-of-the-art
PVD: pressure vessel design
SRP: speed reducer problem
CSD: tension/compression spring design
PLD: piston lever design
CSP: car side impact problem
BCP: bulk carriers problem
WBP: welded beam problem
CBP: cantilever beam problem
TCP: tubular column problem
GTP: gear train problem
VAGWO: velocity-aided grey wolf optimizer
ML: machine learning
CPO: crested porcupine optimizer
COA: coati optimization algorithm
HO: hippopotamus optimization algorithm
LSHADE: Linear population size reduction success-history based adaptive differential evolution
ALSHADE: adaptive LSHADE
CMA-ES: covariance matrix self-adaptation evolutionary algorithm
AO: aquila optimizer
RAS: reptile search algorithm
GTO: artificial gorilla troops optimizer
SMA: slime mould algorithm
BMO: barnacles mating optimizer
BSSA: bear smell search algorithm
BWOA: black widow optimization algorithm
MRFO: manta ray foraging optimization
MPA: marine predators algorithm
MA: mayfly algorithm
RWP: real-world problem
