# Supplementary material for: An improved FOX optimization algorithm using adaptive exploration and exploitation for global optimization
Source: PLoS One. 2025 Sep 18;20(9):e0331965. doi: 10.1371/journal.pone.0331965 (PMC12445531; doi:10.1371/journal.pone.0331965)
Supplement: S1 File — This appendix includes all convergence plots, as well as the figures and tables for the non-parametric statistical analyses of the conducted experiments. (PDF) [file pone.0331965.s001.pdf]

Appendix for: An Improved FOX Optimization Algorithm Using Adaptive Exploration and Exploitation for Global Optimization

Mahmood A. Jumaah[1*], Yossra H. Ali[1], Tarik A. Rashid[2],

1 Department of Computer Science, University of Technology, Baghdad 10066, Iraq
2 Department of Computer Science and Engineering, AIIC, University of Kurdistan Hewlêr, Erbil 44001, Iraq
* cs.22.27@uotechnology.edu.iq

A Supplementary materials

This appendix provides supplementary material for the experiments presented in this paper, organized as follows:

- **Section A.1** shows the convergence plots for all six benchmark groups: classical (CL), CEC 2017 (C17), CEC 2019 (C19), CEC 2021 (C21), CEC 2022 (C22), and real-world problems (RWPs). Each subfigure displays the average convergence over independent runs, with the red curve denoting IFOX's performance.
- **Section A.2** presents statistical-analysis figures. Every figure has two parts: (left) the average algorithm rankings by the Friedman test, and (right) a heatmap of pairwise Wilcoxon signed-rank test p-values, where lighter shades indicate more significant results.
- **Section A.3** provides numerical tables of Wilcoxon signed-rank test results for each benchmark group, listing positive ranks (R^+), negative ranks (R^-), and two-sided p-values.
- **Section A.4** gives tables of the weighted Wilcoxon signed-rank test for each optimization algorithm against all others.

A.1 Convergence figures

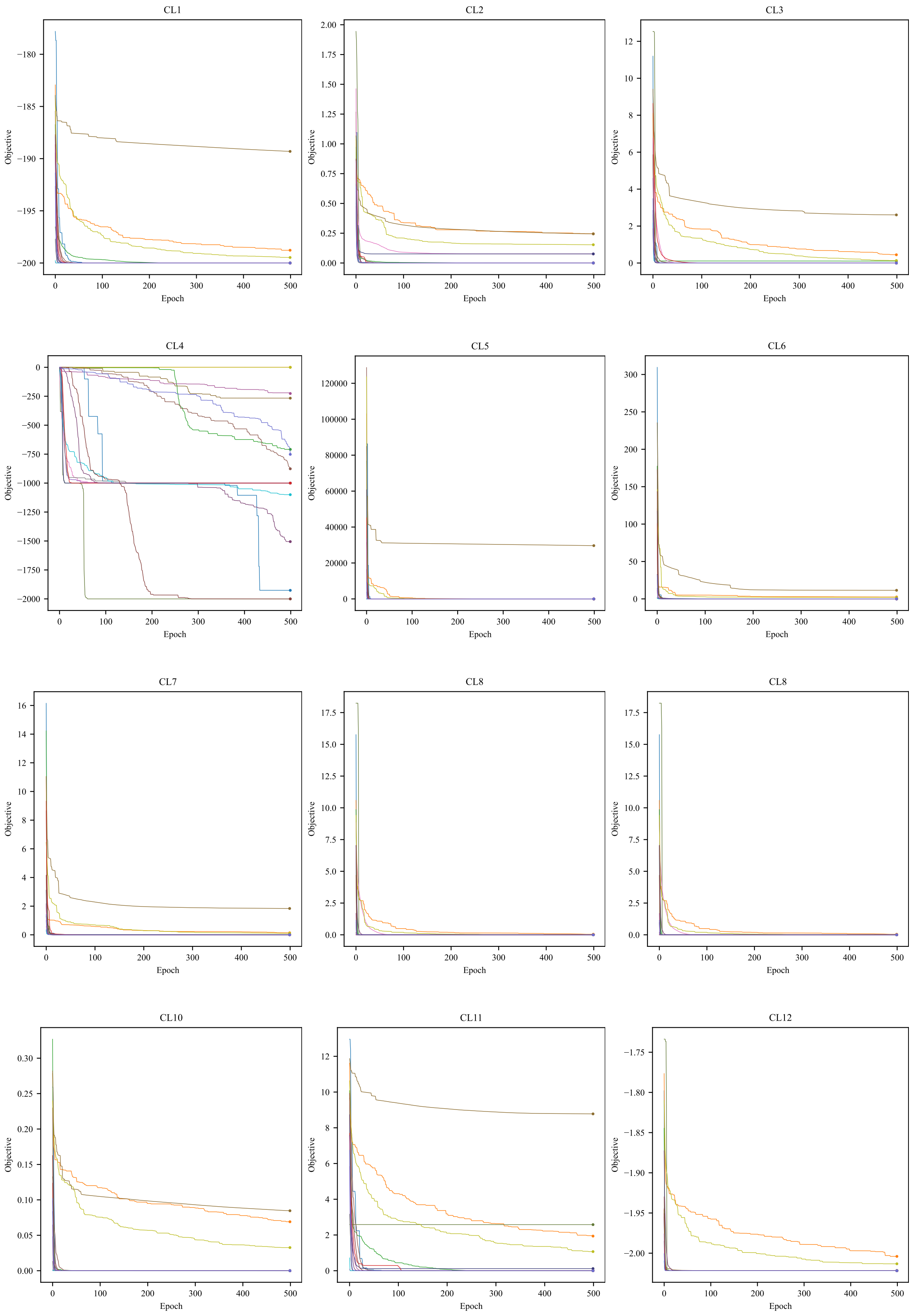

continued on next page . . .

... continued from previous page

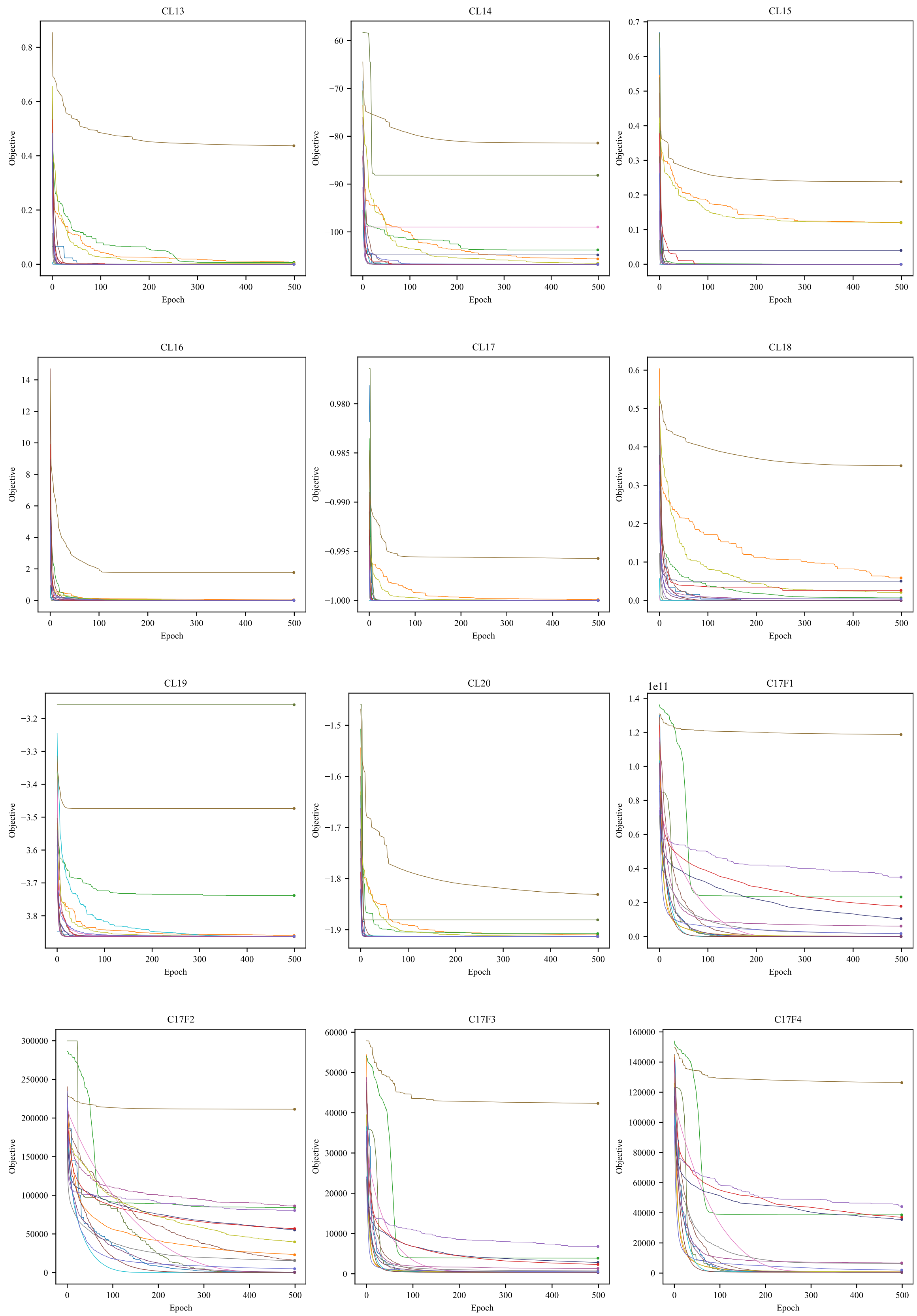

continued on next page ...

. . . continued from previous page

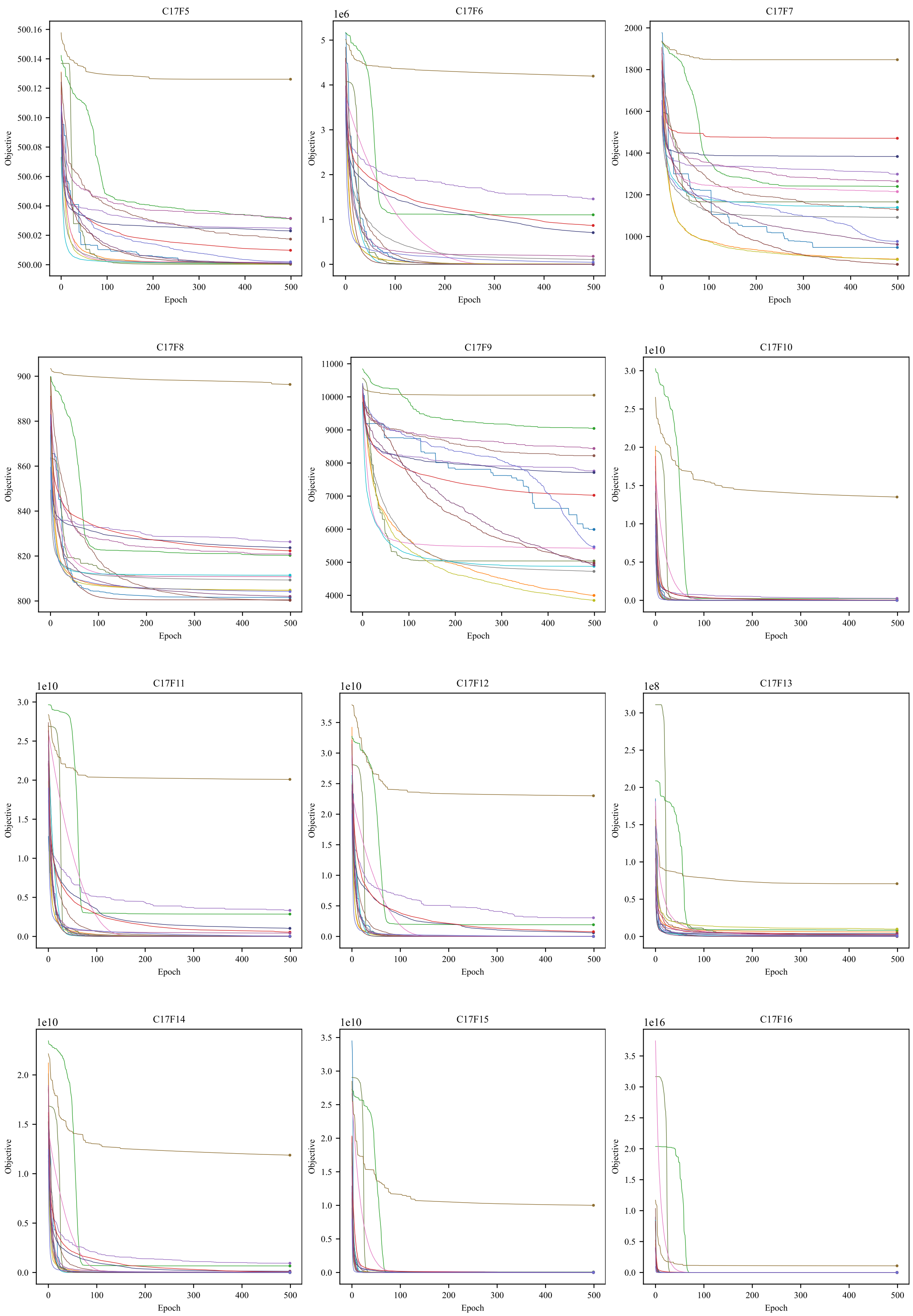

continued on next page . . .

... continued from previous page

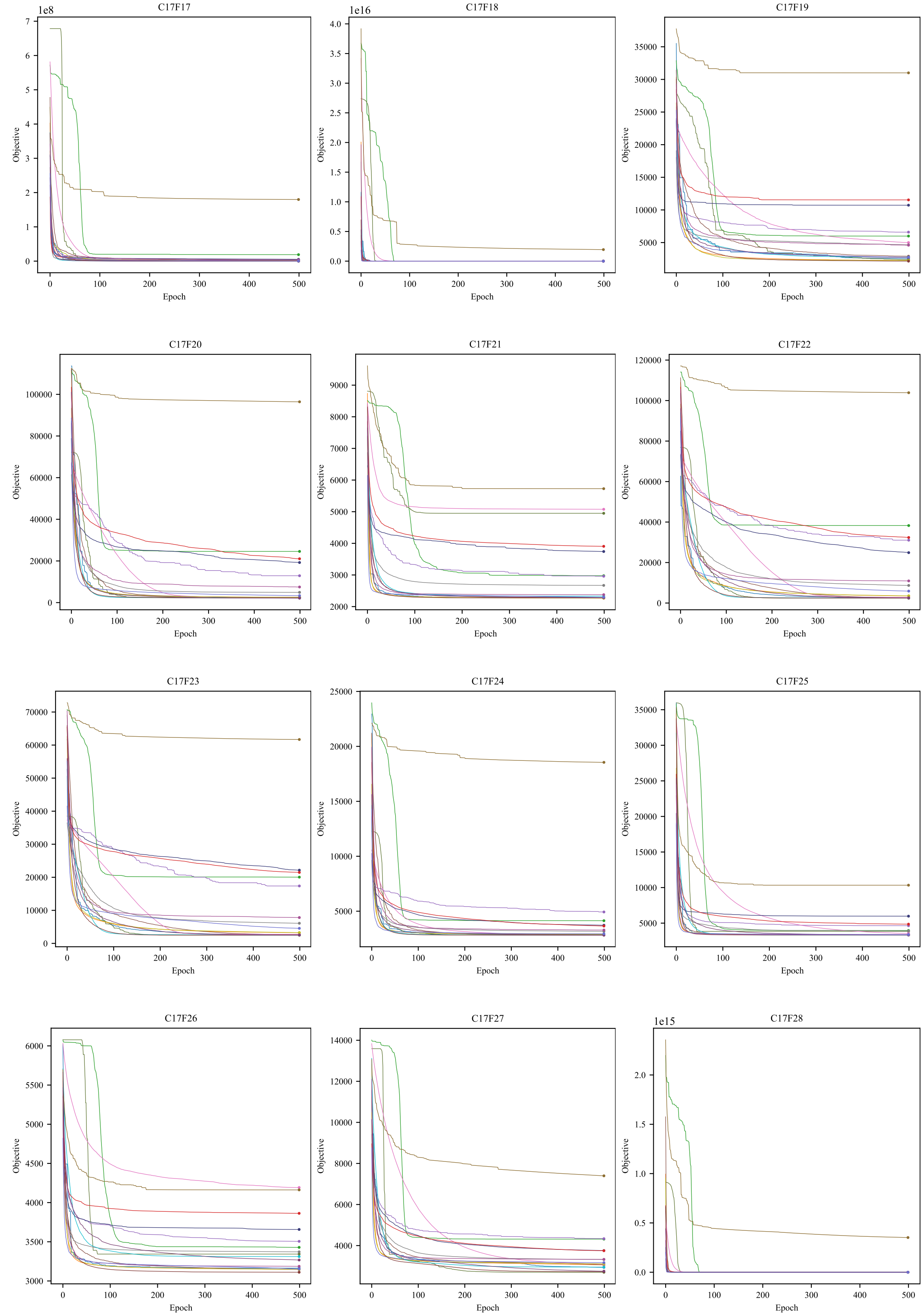

continued on next page ...

... continued from previous page

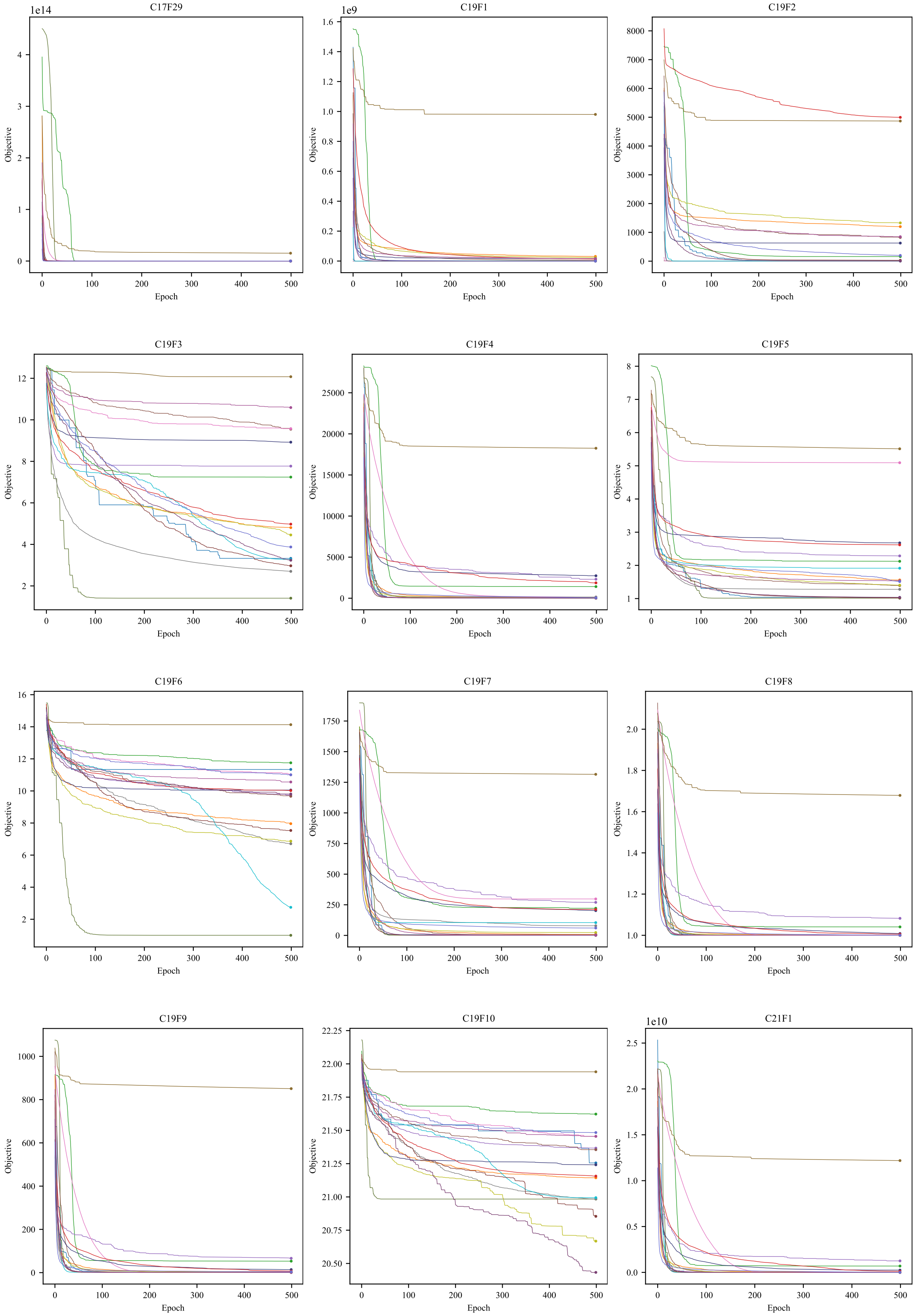

continued on next page ...

... continued from previous page

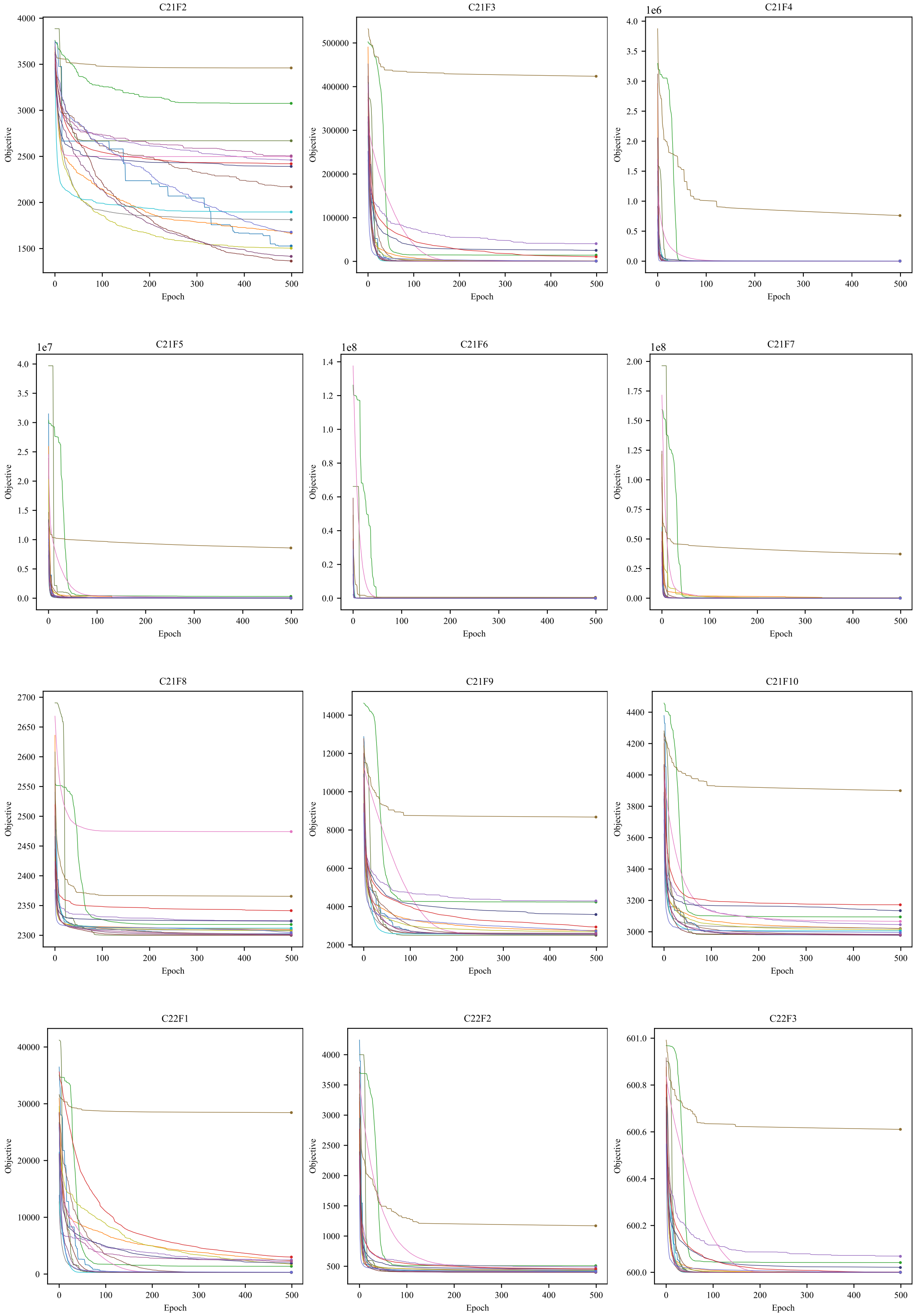

continued on next page ...

... continued from previous page

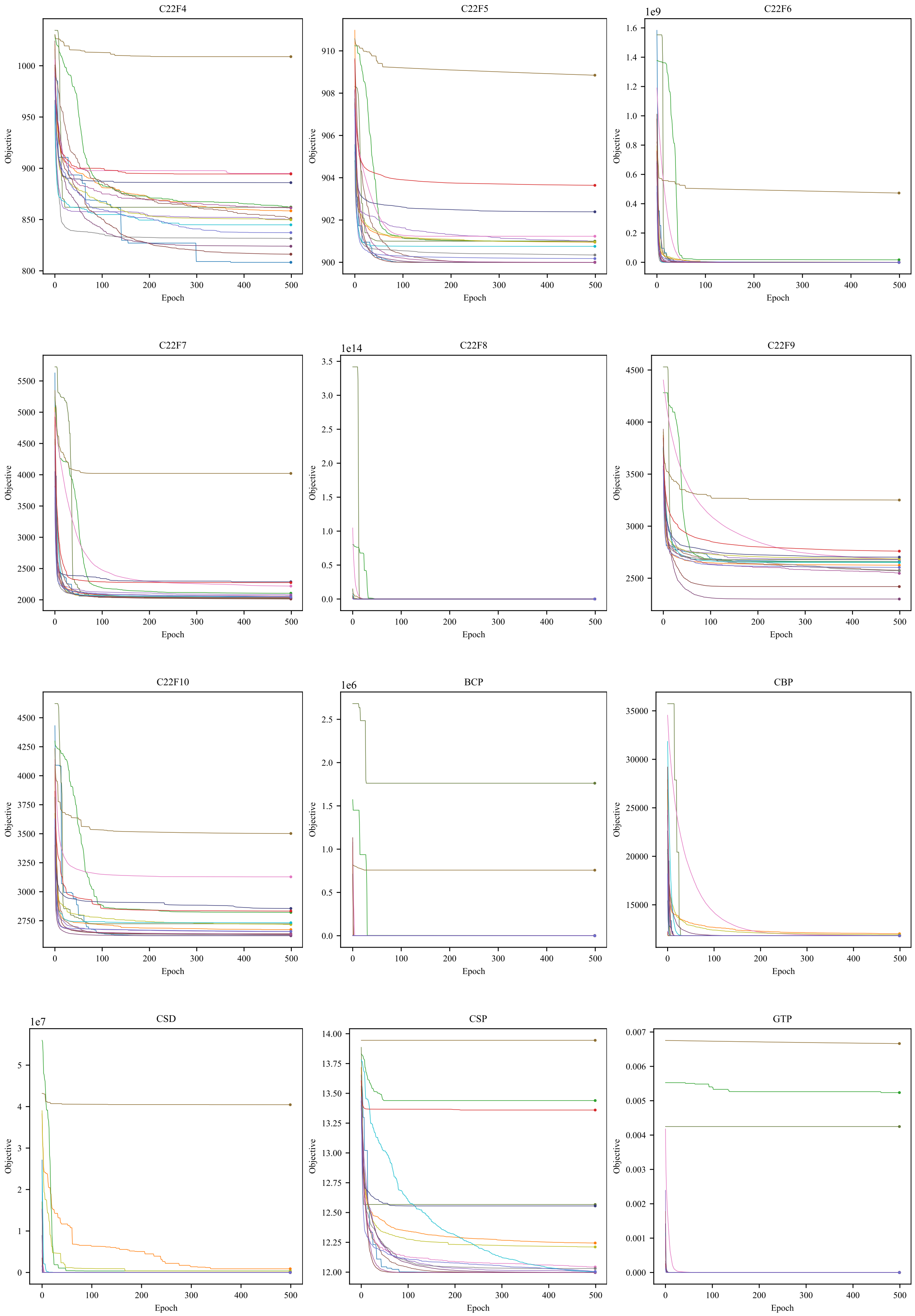

continued on next page ...

... continued from previous page

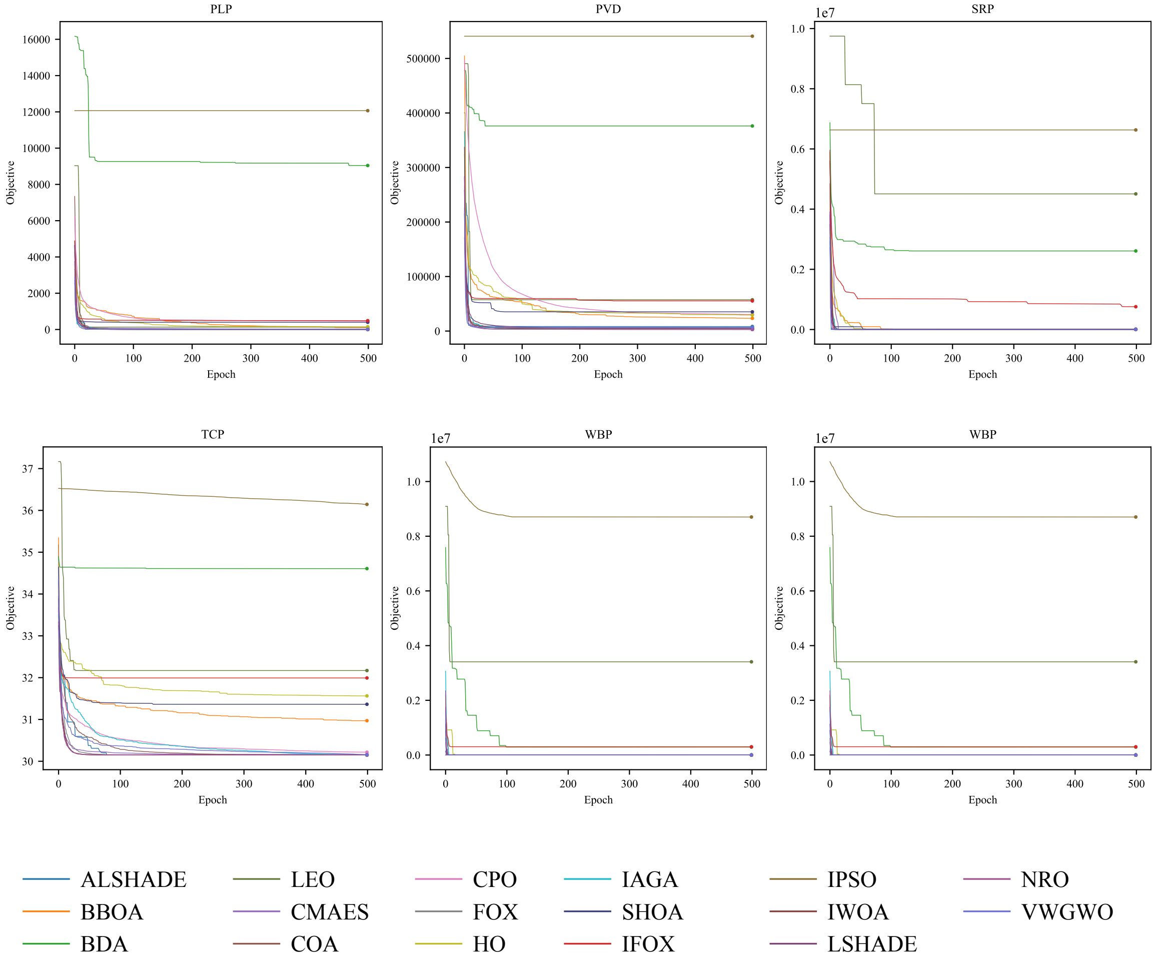

Figure 1: Convergence performance of all optimization algorithms across benchmark problems. Each subfigure corresponds to a test function from the CL, C17, C19, C21, C22, or RWPs. The plots illustrate the average convergence behavior over multiple independent runs, emphasizing the performance of IFOX (red) relative to other algorithms.

A.2 Statistical rankings and heatmaps figures

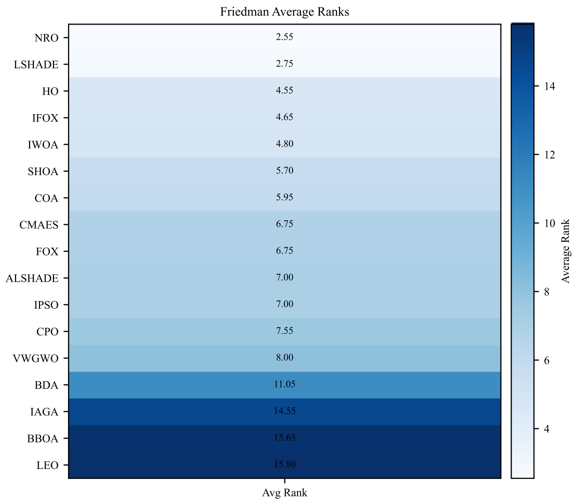

(a) Friedman test rankings

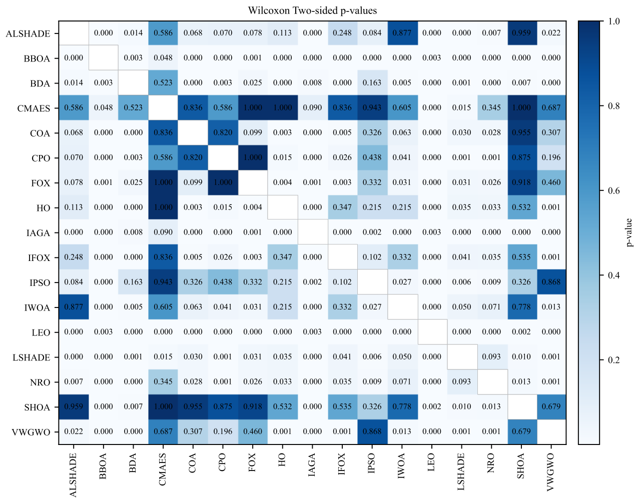

(b) Wilcoxon signed-rank p-values

Figure 2: Non-parametric statistical comparison on classical benchmark test functions. The Friedman heatmap ranks optimizers based on average performance, while the Wilcoxon heatmap highlights statistically significant pairwise differences ($p < 0.05$).

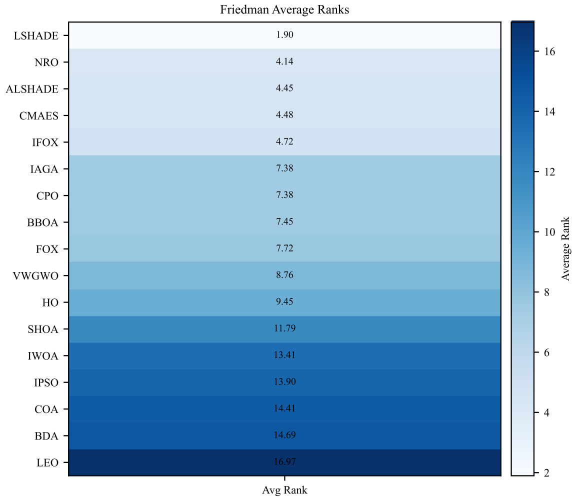

(a) Friedman test rankings

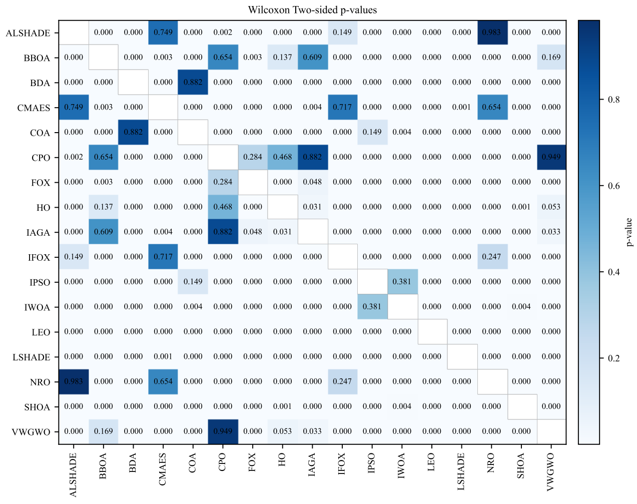

(b) Wilcoxon signed-rank p-values

Figure 3: Statistical analysis on CEC 2017 benchmark functions. Friedman rankings reflect overall optimizer performance, while Wilcoxon p-values assess the significance of differences between algorithm pairs.

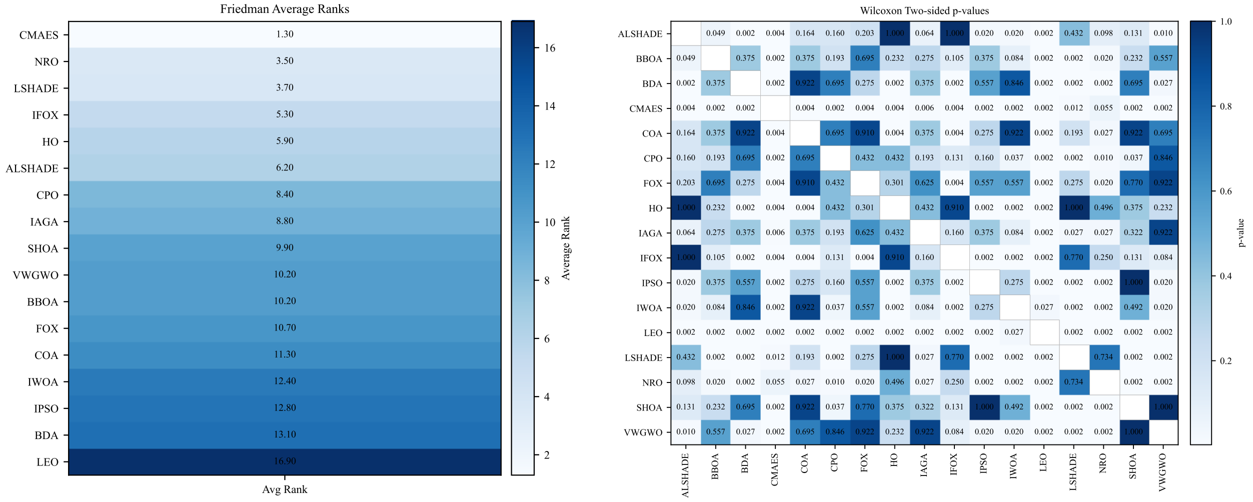

(a) Friedman test rankings

(b) Wilcoxon signed-rank p-values

Figure 4: Statistical comparison of optimizers over CEC 2019 benchmark suite. The rankings and significance tests help reveal consistent outperformers and distinguish subtle performance differences.

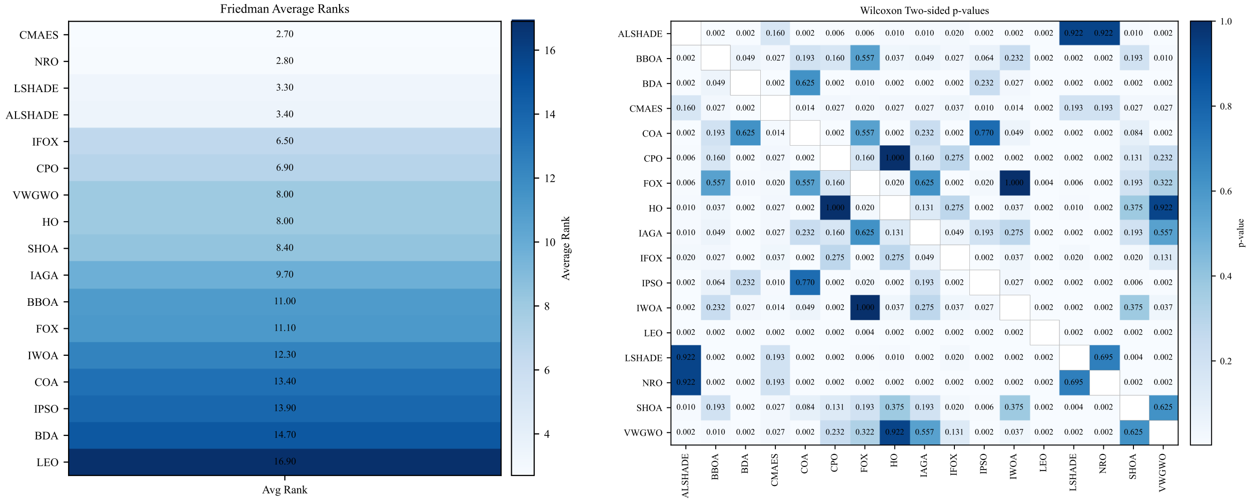

(a) Friedman test rankings

(b) Wilcoxon signed-rank p-values

Figure 5: Statistical analysis on the CEC 2021 test suite. The Friedman ranking identifies the best overall performers, while Wilcoxon tests detect statistically significant pairwise differences.

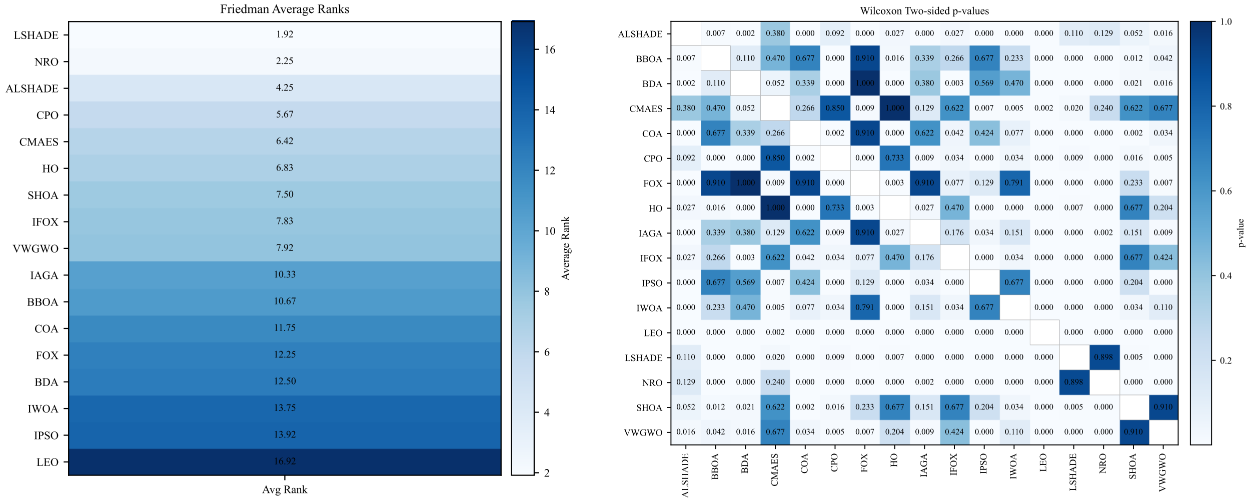

(a) Friedman test rankings

(b) Wilcoxon signed-rank p-values

Figure 6: Evaluation of optimizer performance on CEC 2022 benchmarks using non-parametric tests. Results reveal relative rankings and the robustness of statistical differences.

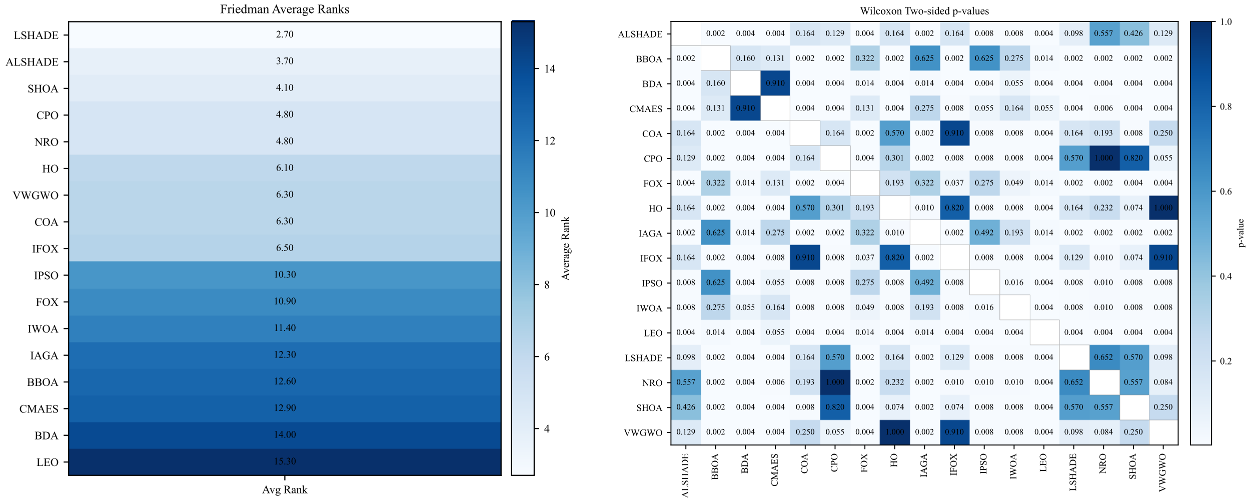

(a) Friedman test rankings

(b) Wilcoxon signed-rank p-values

Figure 7: Statistical results for real-world optimization problems. The Friedman and Wilcoxon tests assess optimizer generalization ability beyond synthetic benchmarks.

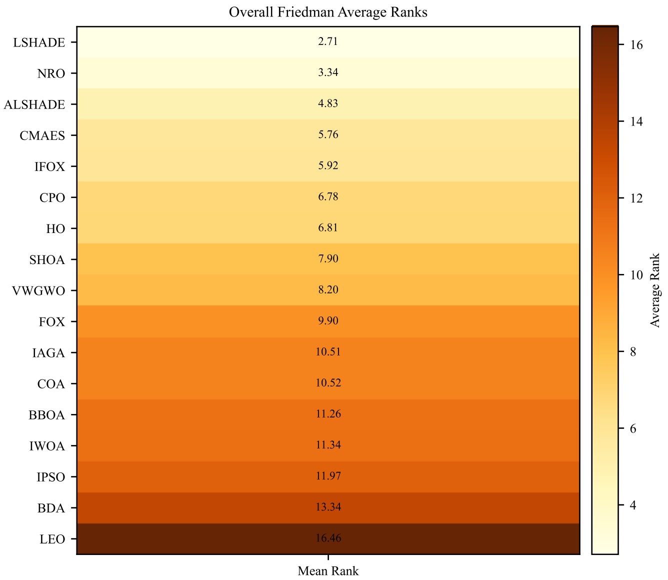

(a) Friedman overall mean ranking

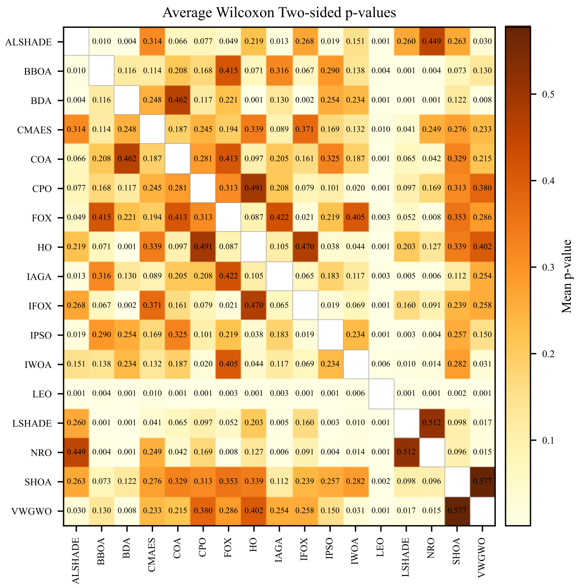

(b) Average Wilcoxon p-values

Figure 8: Overall non-parametric statistical analysis across all benchmark suites and real-world problems. These aggregated plots summarize algorithm performance and highlight consistent statistical dominance.

A.3 Pairwise Wilcoxon signed-rank test tables

Table 1: Two-sided Wilcoxon p-values for pairwise comparisons of each algorithm against the others on classical benchmark test functions.

	ALSHADE	BBOA	BDA	LEO	CMAES	COA	CPO	FOX	HO	IAGA	SHOA	IFOX	IPSO	IWOA	LSHADE	NRO	VWGWO
**ALSHADE**		0.000	0.014	0.000	0.586	0.068	0.070	0.078	0.113	0.000	0.959	0.248	0.084	0.877	0.000	0.007	0.022
**BBOA**	0.000		0.003	0.003	0.048	0.000	0.000	0.001	0.000	0.000	0.000	0.000	0.000	0.000	0.000	0.000	0.000
**BDA**	0.014	0.003		0.000	0.523	0.000	0.003	0.025	0.000	0.008	0.007	0.000	0.163	0.005	0.001	0.000	0.000
**LEO**	0.000	0.003	0.000		0.000	0.000	0.000	0.000	0.000	0.003	0.002	0.000	0.000	0.000	0.000	0.000	0.000
**CMAES**	0.586	0.048	0.523	0.000		0.836	0.586	1.000	1.000	0.090	1.000	0.836	0.943	0.605	0.015	0.345	0.687
**COA**	0.068	0.000	0.000	0.000	0.836		0.820	0.099	0.003	0.000	0.955	0.005	0.326	0.063	0.030	0.028	0.307
**CPO**	0.070	0.000	0.003	0.000	0.586	0.820		1.000	0.015	0.000	0.875	0.026	0.438	0.041	0.001	0.001	0.196
**FOX**	0.078	0.001	0.025	0.000	1.000	0.099	1.000		0.004	0.001	0.918	0.003	0.332	0.031	0.031	0.026	0.460
**HO**	0.113	0.000	0.000	0.000	1.000	0.003	0.015	0.004		0.000	0.532	0.347	0.215	0.215	0.035	0.033	0.001
**IAGA**	0.000	0.000	0.008	0.003	0.090	0.000	0.000	0.001	0.000		0.000	0.000	0.002	0.000	0.000	0.000	0.000
**SHOA**	0.959	0.000	0.007	0.002	1.000	0.955	0.875	0.918	0.532	0.000		0.535	0.326	0.778	0.010	0.013	0.679
**IFOX**	0.248	0.000	0.000	0.000	0.836	0.005	0.026	0.003	0.347	0.000	0.535		0.102	0.332	0.041	0.035	0.001
**IPSO**	0.084	0.000	0.163	0.000	0.943	0.326	0.438	0.332	0.215	0.002	0.326	0.102		0.027	0.006	0.009	0.868
**IWOA**	0.877	0.000	0.005	0.000	0.605	0.063	0.041	0.031	0.215	0.000	0.778	0.332	0.027		0.050	0.071	0.013
**LSHADE**	0.000	0.000	0.001	0.000	0.015	0.030	0.001	0.031	0.035	0.000	0.010	0.041	0.006	0.050		0.093	0.001
**NRO**	0.007	0.000	0.000	0.000	0.345	0.028	0.001	0.026	0.033	0.000	0.013	0.035	0.009	0.071	0.093		0.001
**VWGWO**	0.022	0.000	0.000	0.000	0.687	0.307	0.196	0.460	0.001	0.000	0.679	0.001	0.868	0.013	0.001	0.001	

Table 2: Two-sided Wilcoxon p-values for pairwise comparisons of each algorithm against the others on CEC 2017 benchmark test functions.

	ALSHADE	BBOA	BDA	LEO	CMAES	COA	CPO	FOX	HO	IAGA	SHOA	IFOX	IPSO	IWOA	LSHADE	NRO	VWGWO
**ALSHADE**		0.000	0.000	0.000	0.749	0.000	0.002	0.000	0.000	0.000	0.000	0.149	0.000	0.000	0.000	0.983	0.000
**BBOA**	0.000		0.000	0.000	0.003	0.000	0.654	0.003	0.137	0.609	0.000	0.000	0.000	0.000	0.000	0.000	0.169
**BDA**	0.000	0.000		0.000	0.000	0.882	0.000	0.000	0.000	0.000	0.000	0.000	0.000	0.000	0.000	0.000	0.000
**LEO**	0.000	0.000	0.000		0.000	0.000	0.000	0.000	0.000	0.000	0.000	0.000	0.000	0.000	0.000	0.000	0.000
**CMAES**	0.749	0.003	0.000	0.000		0.000	0.000	0.000	0.000	0.004	0.000	0.717	0.000	0.000	0.001	0.654	0.000
**COA**	0.000	0.000	0.882	0.000	0.000		0.000	0.000	0.000	0.000	0.000	0.000	0.149	0.004	0.000	0.000	0.000
**CPO**	0.002	0.654	0.000	0.000	0.000	0.000		0.284	0.468	0.882	0.000	0.000	0.000	0.000	0.000	0.000	0.949
**FOX**	0.000	0.003	0.000	0.000	0.000	0.000	0.284		0.000	0.048	0.000	0.000	0.000	0.000	0.000	0.000	0.000
**HO**	0.000	0.137	0.000	0.000	0.000	0.000	0.468	0.000		0.031	0.001	0.000	0.000	0.000	0.000	0.000	0.053
**IAGA**	0.000	0.609	0.000	0.000	0.004	0.000	0.882	0.048	0.031		0.000	0.000	0.000	0.000	0.000	0.000	0.033
**SHOA**	0.000	0.000	0.000	0.000	0.000	0.000	0.000	0.000	0.001	0.000		0.000	0.000	0.004	0.000	0.000	0.000
**IFOX**	0.149	0.000	0.000	0.000	0.717	0.000	0.000	0.000	0.000	0.000	0.000		0.000	0.000	0.000	0.247	0.000
**IPSO**	0.000	0.000	0.000	0.000	0.000	0.149	0.000	0.000	0.000	0.000	0.000	0.000		0.381	0.000	0.000	0.000
**IWOA**	0.000	0.000	0.000	0.000	0.000	0.004	0.000	0.000	0.000	0.000	0.004	0.000	0.381		0.000	0.000	0.000
**LSHADE**	0.000	0.000	0.000	0.000	0.001	0.000	0.000	0.000	0.000	0.000	0.000	0.000	0.000	0.000		0.000	0.000
**NRO**	0.983	0.000	0.000	0.000	0.654	0.000	0.000	0.000	0.000	0.000	0.000	0.247	0.000	0.000	0.000		0.000
**VWGWO**	0.000	0.169	0.000	0.000	0.000	0.000	0.949	0.000	0.053	0.033	0.000	0.000	0.000	0.000	0.000	0.000	

Table 3: Two-sided Wilcoxon p-values for pairwise comparisons of each algorithm against the others on CEC 2019 benchmark test functions.

	ALSHADE	BBOA	BDA	LEO	CMAES	COA	CPO	FOX	HO	IAGA	SHOA	IFOX	IPSO	IWOA	LSHADE	NRO	VWGWO
**ALSHADE**		0.049	0.002	0.002	0.004	0.164	0.160	0.203	1.000	0.064	0.131	1.000	0.020	0.020	0.432	0.098	0.010
**BBOA**	0.049		0.375	0.002	0.002	0.375	0.193	0.695	0.232	0.275	0.232	0.105	0.375	0.084	0.002	0.020	0.557
**BDA**	0.002	0.375		0.002	0.002	0.922	0.695	0.275	0.002	0.375	0.695	0.002	0.557	0.846	0.002	0.002	0.027
**LEO**	0.002	0.002	0.002		0.002	0.002	0.002	0.002	0.002	0.002	0.002	0.002	0.002	0.027	0.002	0.002	0.002
**CMAES**	0.004	0.002	0.002	0.002		0.004	0.002	0.004	0.004	0.006	0.002	0.004	0.002	0.002	0.012	0.055	0.002
**COA**	0.164	0.375	0.922	0.002	0.004		0.695	0.910	0.004	0.375	0.922	0.004	0.275	0.922	0.193	0.027	0.695
**CPO**	0.160	0.193	0.695	0.002	0.002	0.695		0.432	0.432	0.193	0.037	0.131	0.160	0.037	0.002	0.010	0.846
**FOX**	0.203	0.695	0.275	0.002	0.004	0.910	0.432		0.301	0.625	0.770	0.004	0.557	0.557	0.275	0.020	0.922
**HO**	1.000	0.232	0.002	0.002	0.004	0.004	0.432	0.301		0.432	0.375	0.910	0.002	0.002	1.000	0.496	0.232
**IAGA**	0.064	0.275	0.375	0.002	0.006	0.375	0.193	0.625	0.432		0.322	0.160	0.375	0.084	0.027	0.027	0.922
**SHOA**	0.131	0.232	0.695	0.002	0.002	0.922	0.037	0.770	0.375	0.322		0.131	1.000	0.492	0.002	0.002	1.000
**IFOX**	1.000	0.105	0.002	0.002	0.004	0.004	0.131	0.004	0.910	0.160	0.131		0.002	0.002	0.770	0.250	0.084
**IPSO**	0.020	0.375	0.557	0.002	0.002	0.275	0.160	0.557	0.002	0.375	1.000	0.002		0.275	0.002	0.002	0.020
**IWOA**	0.020	0.084	0.846	0.027	0.002	0.922	0.037	0.557	0.002	0.084	0.492	0.002	0.275		0.002	0.002	0.020
**LSHADE**	0.432	0.002	0.002	0.002	0.012	0.193	0.002	0.275	1.000	0.027	0.002	0.770	0.002	0.002		0.734	0.002
**NRO**	0.098	0.020	0.002	0.002	0.055	0.027	0.010	0.020	0.496	0.027	0.002	0.250	0.002	0.002	0.734		0.002
**VWGWO**	0.010	0.557	0.027	0.002	0.002	0.695	0.846	0.922	0.232	0.922	1.000	0.084	0.020	0.020	0.002	0.002	

Table 4: Two-sided Wilcoxon p-values for pairwise comparisons of each algorithm against the others on CEC 2021 benchmark test functions.

	**ALSHADE**	**BBOA**	**BDA**	**LEO**	**CMAES**	**COA**	**CPO**	**FOX**	**HO**	**IAGA**	**SHOA**	**IFOX**	**IPSO**	**IWOA**	**LSHADE**	**NRO**	**VWGWO**
**ALSHADE**		0.002	0.002	0.002	0.160	0.002	0.006	0.006	0.010	0.010	0.010	0.020	0.002	0.002	0.922	0.922	0.002
**BBOA**	0.002		0.049	0.002	0.027	0.193	0.160	0.557	0.037	0.049	0.193	0.027	0.064	0.232	0.002	0.002	0.010
**BDA**	0.002	0.049		0.002	0.002	0.625	0.002	0.010	0.002	0.002	0.002	0.002	0.232	0.027	0.002	0.002	0.002
**LEO**	0.002	0.002	0.002		0.002	0.002	0.002	0.004	0.002	0.002	0.002	0.002	0.002	0.002	0.002	0.002	0.002
**CMAES**	0.160	0.027	0.002	0.002		0.014	0.027	0.020	0.027	0.027	0.027	0.037	0.010	0.014	0.193	0.193	0.027
**COA**	0.002	0.193	0.625	0.002	0.014		0.002	0.557	0.002	0.232	0.084	0.002	0.770	0.049	0.002	0.002	0.002
**CPO**	0.006	0.160	0.002	0.002	0.027	0.002		0.160	1.000	0.160	0.131	0.275	0.002	0.002	0.002	0.002	0.232
**FOX**	0.006	0.557	0.010	0.004	0.020	0.557	0.160		0.020	0.625	0.193	0.002	0.020	1.000	0.006	0.002	0.322
**HO**	0.010	0.037	0.002	0.002	0.027	0.002	1.000	0.020		0.131	0.375	0.275	0.002	0.037	0.010	0.002	0.922
**IAGA**	0.010	0.049	0.002	0.002	0.027	0.232	0.160	0.625	0.131		0.193	0.049	0.193	0.275	0.002	0.002	0.557
**SHOA**	0.010	0.193	0.002	0.002	0.027	0.084	0.131	0.193	0.375	0.193		0.020	0.006	0.375	0.004	0.002	0.625
**IFOX**	0.020	0.027	0.002	0.002	0.037	0.002	0.275	0.002	0.275	0.049	0.020		0.002	0.037	0.020	0.002	0.131
**IPSO**	0.002	0.064	0.232	0.002	0.010	0.770	0.002	0.020	0.002	0.193	0.006	0.002		0.027	0.002	0.002	0.002
**IWOA**	0.002	0.232	0.027	0.002	0.014	0.049	0.002	1.000	0.037	0.275	0.375	0.037	0.027		0.002	0.002	0.037
**LSHADE**	0.922	0.002	0.002	0.002	0.193	0.002	0.002	0.006	0.010	0.002	0.004	0.020	0.002	0.002		0.695	0.002
**NRO**	0.922	0.002	0.002	0.002	0.193	0.002	0.002	0.002	0.002	0.002	0.002	0.002	0.002	0.002	0.695		0.002
**VWGWO**	0.002	0.010	0.002	0.002	0.027	0.002	0.232	0.322	0.922	0.557	0.625	0.131	0.002	0.037	0.002	0.002	

Table 5: Two-sided Wilcoxon p-values for pairwise comparisons of each algorithm against the others on CEC 2022 benchmark test functions.

	**ALSHADE**	**BBOA**	**BDA**	**LEO**	**CMAES**	**COA**	**CPO**	**FOX**	**HO**	**IAGA**	**SHOA**	**IFOX**	**IPSO**	**IWOA**	**LSHADE**	**NRO**	**VWGWO**
**ALSHADE**		0.007	0.002	0.000	0.380	0.000	0.092	0.000	0.027	0.000	0.052	0.027	0.000	0.000	0.110	0.129	0.016
**BBOA**	0.007		0.110	0.000	0.470	0.677	0.000	0.910	0.016	0.339	0.012	0.266	0.677	0.233	0.000	0.000	0.042
**BDA**	0.002	0.110		0.000	0.052	0.339	0.000	1.000	0.000	0.380	0.021	0.003	0.569	0.470	0.000	0.000	0.016
**LEO**	0.000	0.000	0.000		0.002	0.000	0.000	0.000	0.000	0.000	0.000	0.000	0.000	0.000	0.000	0.000	0.000
**CMAES**	0.380	0.470	0.052	0.002		0.266	0.850	0.009	1.000	0.129	0.622	0.622	0.007	0.005	0.020	0.240	0.677
**COA**	0.000	0.677	0.339	0.000	0.266		0.002	0.910	0.000	0.622	0.002	0.042	0.424	0.077	0.000	0.000	0.034
**CPO**	0.092	0.000	0.000	0.000	0.850	0.002		0.000	0.733	0.009	0.016	0.034	0.000	0.034	0.009	0.000	0.005
**FOX**	0.000	0.910	1.000	0.000	0.009	0.910	0.000		0.003	0.910	0.233	0.077	0.129	0.791	0.000	0.000	0.007
**HO**	0.027	0.016	0.000	0.000	1.000	0.000	0.733	0.003		0.027	0.677	0.470	0.000	0.000	0.007	0.000	0.204
**IAGA**	0.000	0.339	0.380	0.000	0.129	0.622	0.009	0.910	0.027		0.151	0.176	0.034	0.151	0.000	0.002	0.009
**SHOA**	0.052	0.012	0.021	0.000	0.622	0.002	0.016	0.233	0.677	0.151		0.677	0.204	0.034	0.005	0.000	0.910
**IFOX**	0.027	0.266	0.003	0.000	0.622	0.042	0.034	0.077	0.470	0.176	0.677		0.000	0.034	0.000	0.000	0.424
**IPSO**	0.000	0.677	0.569	0.000	0.007	0.424	0.000	0.129	0.000	0.034	0.204	0.000		0.677	0.000	0.000	0.000
**IWOA**	0.000	0.233	0.470	0.000	0.005	0.077	0.034	0.791	0.000	0.151	0.034	0.034	0.677		0.000	0.000	0.110
**LSHADE**	0.110	0.000	0.000	0.000	0.020	0.000	0.009	0.000	0.007	0.000	0.005	0.000	0.000	0.000		0.898	0.000
**NRO**	0.129	0.000	0.000	0.000	0.240	0.000	0.000	0.000	0.000	0.002	0.000	0.000	0.000	0.000	0.898		0.000
**VWGWO**	0.016	0.042	0.016	0.000	0.677	0.034	0.005	0.007	0.204	0.009	0.910	0.424	0.000	0.110	0.000	0.000	

Table 6: Two-sided Wilcoxon p-values for pairwise comparisons of each algorithm against the others on real-world problems.

	ALSHADE	BBOA	BDA	LEO	CMAES	COA	CPO	FOX	HO	IAGA	SHOA	IFOX	IPSO	IWOA	LSHADE	NRO	VWGWO
**ALSHADE**		0.002	0.004	0.004	0.004	0.164	0.129	0.004	0.164	0.002	0.426	0.164	0.008	0.008	0.098	0.557	0.129
**BBOA**	0.002		0.160	0.014	0.131	0.002	0.002	0.322	0.002	0.625	0.002	0.002	0.625	0.275	0.002	0.002	0.002
**BDA**	0.004	0.160		0.004	0.910	0.004	0.004	0.014	0.004	0.014	0.004	0.004	0.004	0.055	0.004	0.004	0.004
**LEO**	0.004	0.014	0.004		0.055	0.004	0.004	0.014	0.004	0.014	0.004	0.004	0.004	0.004	0.004	0.004	0.004
**CMAES**	0.004	0.131	0.910	0.055		0.004	0.004	0.131	0.004	0.275	0.004	0.008	0.055	0.164	0.004	0.006	0.004
**COA**	0.164	0.002	0.004	0.004	0.004		0.164	0.002	0.570	0.002	0.008	0.910	0.008	0.008	0.164	0.193	0.250
**CPO**	0.129	0.002	0.004	0.004	0.004	0.164		0.004	0.301	0.002	0.820	0.008	0.008	0.008	0.570	1.000	0.055
**FOX**	0.004	0.322	0.014	0.014	0.131	0.002	0.004		0.193	0.322	0.004	0.037	0.275	0.049	0.002	0.002	0.004
**HO**	0.164	0.002	0.004	0.004	0.004	0.570	0.301	0.193		0.010	0.074	0.820	0.008	0.008	0.164	0.232	1.000
**IAGA**	0.002	0.625	0.014	0.014	0.275	0.002	0.002	0.322	0.010		0.002	0.002	0.492	0.193	0.002	0.002	0.002
**SHOA**	0.426	0.002	0.004	0.004	0.004	0.008	0.820	0.004	0.074	0.002		0.074	0.008	0.008	0.570	0.557	0.250
**IFOX**	0.164	0.002	0.004	0.004	0.008	0.910	0.008	0.037	0.820	0.002	0.074		0.008	0.008	0.129	0.010	0.910
**IPSO**	0.008	0.625	0.004	0.004	0.055	0.008	0.008	0.275	0.008	0.492	0.008	0.008		0.016	0.008	0.010	0.008
**IWOA**	0.008	0.275	0.055	0.004	0.164	0.008	0.008	0.049	0.008	0.193	0.008	0.008	0.016		0.008	0.010	0.008
**LSHADE**	0.098	0.002	0.004	0.004	0.004	0.164	0.570	0.002	0.164	0.002	0.570	0.129	0.008	0.008		0.652	0.098
**NRO**	0.557	0.002	0.004	0.004	0.006	0.193	1.000	0.002	0.232	0.002	0.557	0.010	0.010	0.010	0.652		0.084
**VWGWO**	0.129	0.002	0.004	0.004	0.004	0.250	0.055	0.004	1.000	0.002	0.250	0.910	0.008	0.008	0.098	0.084	

Table 7: Average two-sided Wilcoxon p-values for pairwise comparisons of each algorithm against the others.

	ALSHADE	BBOA	BDA	LEO	CMAES	COA	CPO	FOX	HO	IAGA	SHOA	IFOX	IPSO	IWOA	LSHADE	NRO	VWGWO
**ALSHADE**		0.010	0.004	0.001	0.314	0.066	0.077	0.049	0.219	0.013	0.263	0.268	0.019	0.151	0.260	0.449	0.030
**BBOA**	0.010		0.116	0.004	0.114	0.208	0.168	0.415	0.071	0.316	0.073	0.067	0.290	0.138	0.001	0.004	0.130
**BDA**	0.004	0.116		0.001	0.248	0.462	0.117	0.221	0.001	0.130	0.122	0.002	0.254	0.234	0.001	0.001	0.008
**LEO**	0.001	0.004	0.001		0.010	0.001	0.001	0.003	0.001	0.003	0.002	0.001	0.001	0.006	0.001	0.001	0.001
**CMAES**	0.314	0.114	0.248	0.010		0.187	0.245	0.194	0.339	0.089	0.276	0.371	0.169	0.132	0.041	0.249	0.233
**COA**	0.066	0.208	0.462	0.001	0.187		0.281	0.413	0.097	0.205	0.329	0.161	0.325	0.187	0.065	0.042	0.215
**CPO**	0.077	0.168	0.117	0.001	0.245	0.281		0.313	0.491	0.208	0.313	0.079	0.101	0.020	0.097	0.169	0.380
**FOX**	0.049	0.415	0.221	0.003	0.194	0.413	0.313		0.087	0.422	0.353	0.021	0.219	0.405	0.052	0.008	0.286
**HO**	0.219	0.071	0.001	0.001	0.339	0.097	0.491	0.087		0.105	0.339	0.470	0.038	0.044	0.203	0.127	0.402
**IAGA**	0.013	0.316	0.130	0.003	0.089	0.205	0.208	0.422	0.105		0.112	0.065	0.183	0.117	0.005	0.006	0.254
**SHOA**	0.263	0.073	0.122	0.002	0.276	0.329	0.313	0.353	0.339	0.112		0.239	0.257	0.282	0.098	0.096	0.577
**IFOX**	0.268	0.067	0.002	0.001	0.371	0.161	0.079	0.021	0.470	0.065	0.239		0.019	0.069	0.160	0.091	0.258
**IPSO**	0.019	0.290	0.254	0.001	0.169	0.325	0.101	0.219	0.038	0.183	0.257	0.019		0.234	0.003	0.004	0.150
**IWOA**	0.151	0.138	0.234	0.006	0.132	0.187	0.020	0.405	0.044	0.117	0.282	0.069	0.234		0.010	0.014	0.031
**LSHADE**	0.260	0.001	0.001	0.001	0.041	0.065	0.097	0.052	0.203	0.005	0.098	0.160	0.003	0.010		0.512	0.017
**NRO**	0.449	0.004	0.001	0.001	0.249	0.042	0.169	0.008	0.127	0.006	0.096	0.091	0.004	0.014	0.512		0.015
**VWGWO**	0.030	0.130	0.008	0.001	0.233	0.215	0.380	0.286	0.402	0.254	0.577	0.258	0.150	0.031	0.017	0.015	

A.4 Algorithm-vs-algorithm statistical analyses

Table 8: Wilcoxon signed-rank test results comparing IFOX with other optimization algorithms across CL, C17, C19, C21, C22, and RWPs.

Wilcoxon signed-rank test of IFOX versus optimization algorithms																		
**Algorithm**	**CL**			**C17**			**C19**			**C21**			**C22**			**RWPs**		
	R^+	R^-	**P-val**	R^+	R^-	**P-val**	R^+	R^-	**P-val**	R^+	R^-	**P-val**	R^+	R^-	**P-val**	R^+	R^-	**P-val**
ALSHADE	59	112	0.25	150	285	0.15	22.0	23.0	1.00	5	50	0.02	11	67	0.03	10	35	0.16
BBOA	210	0	0.00	382	53	0.00	44.0	11.0	0.11	49	6	0.03	54	24	0.27	55	0	0.00
BDA	153	0	0.00	435	0	0.00	55.0	0.0	0.00	55	0	0.00	74	4	0.00	45	0	0.00
LEO	190	0	0.00	435	0	0.00	55.0	0.0	0.00	55	0	0.00	78	0	0.00	45	0	0.00
CMAES	72	64	0.84	200	235	0.72	0.0	45.0	0.00	7	48	0.04	32	46	0.62	44	1	0.01
COA	75	3	0.00	435	0	0.00	45.0	0.0	0.00	55	0	0.00	65	13	0.04	21	24	0.91
CPO	111	25	0.03	399	36	0.00	43.0	12.0	0.13	39	16	0.28	12	66	0.03	1	44	0.01
FOX	66	0	0.00	429	6	0.00	45.0	0.0	0.00	55	0	0.00	62	16	0.08	48	7	0.04
HO	51	27	0.35	424	11	0.00	24.0	21.0	0.91	39	16	0.28	29	49	0.47	20	25	0.82
IAGA	210	0	0.00	383	52	0.00	42.0	13.0	0.16	47	8	0.05	57	21	0.18	55	0	0.00
SHOA	80	56	0.53	430	5	0.00	43.0	12.0	0.13	50	5	0.02	45	33	0.68	7	38	0.07
IPSO	111	42	0.10	435	0	0.00	55.0	0.0	0.00	55	0	0.00	78	0	0.00	44	1	0.01
IWOA	56	97	0.33	435	0	0.00	55.0	0.0	0.00	48	7	0.04	66	12	0.03	44	1	0.01
LSHADE	24	96	0.04	49	386	0.00	24.0	31.0	0.77	5	50	0.02	0	78	0.00	9	36	0.13
NRO	19	86	0.04	163	272	0.25	12.0	33.0	0.25	0	55	0.00	0	78	0.00	3	52	0.01
VWGWO	120	0	0.00	409	26	0.00	45.0	10.0	0.08	43	12	0.13	28	50	0.42	24	21	0.91

Table 9: Wilcoxon signed-rank test results comparing ALSHADE with other optimization algorithms across CL, C17, C19, C21, C22, and RWPs.

Wilcoxon signed-rank test of ALSHADE versus optimization algorithms																		
**Algorithm**	**CL**			**C17**			**C19**			**C21**			**C22**			**RWPs**		
	R^+	R^-	**P-val**	R^+	R^-	**P-val**	R^+	R^-	**P-val**	R^+	R^-	**P-val**	R^+	R^-	**P-val**	R^+	R^-	**P-val**
BBOA	210	0	0.00	390	45	0.00	47	8	0.05	55	0	0.00	72	6	0.01	55	0	0.00
BDA	142	29	0.01	435	0	0.00	55	0	0.00	55	0	0.00	75	3	0.00	45	0	0.00
LEO	190	0	0.00	435	0	0.00	55	0	0.00	55	0	0.00	78	0	0.00	45	0	0.00
CMAES	73	98	0.59	233	202	0.75	0	45	0.00	13	42	0.16	51	27	0.38	45	0	0.00
COA	115	38	0.07	435	0	0.00	35	10	0.16	55	0	0.00	78	0	0.00	35	10	0.16
CPO	103	33	0.07	359	76	0.00	42	13	0.16	53	2	0.01	61	17	0.09	36	9	0.13
FOX	126	45	0.08	394	41	0.00	34	11	0.20	53	2	0.01	78	0	0.00	54	1	0.00
HO	110	43	0.11	424	11	0.00	23	22	1.00	52	3	0.01	67	11	0.03	35	10	0.16
IAGA	203	7	0.00	390	45	0.00	46	9	0.06	52	3	0.01	78	0	0.00	55	0	0.00
SHOA	67	69	0.96	435	0	0.00	43	12	0.13	52	3	0.01	64	14	0.05	30	15	0.43
IFOX	112	59	0.25	285	150	0.15	23	22	1.00	50	5	0.02	67	11	0.03	35	10	0.16
IPSO	113	40	0.08	435	0	0.00	50	5	0.02	55	0	0.00	78	0	0.00	44	1	0.01
IWOA	71	65	0.88	435	0	0.00	50	5	0.02	55	0	0.00	78	0	0.00	44	1	0.01
LSHADE	0	136	0.00	20	415	0.00	19	36	0.43	29	26	0.92	18	60	0.11	8	37	0.10
NRO	16	120	0.01	216	219	0.98	8	37	0.10	29	26	0.92	19	59	0.13	34	21	0.56
VWGWO	138	33	0.02	421	14	0.00	52	3	0.01	55	0	0.00	69	9	0.02	36	9	0.13

Table 10: Wilcoxon signed-rank test results comparing BBOA with other optimization algorithms across CL, C17, C19, C21, C22, and RWPs.

Wilcoxon signed-rank test of BBOA versus optimization algorithms																		
**Algorithm**	**CL**			**C17**			**C19**			**C21**			**C22**			**RWPs**		
	**R^+**	**R^-**	**P-val**	**R^+**	**R^-**	**P-val**	**R^+**	**R^-**	**P-val**	**R^+**	**R^-**	**P-val**	**R^+**	**R^-**	**P-val**	**R^+**	**R^-**	**P-val**
ALSHADE	0	210	0.00	45	390	0.00	8	47	0.05	0	55	0.00	6	72	0.01	0	55	0.00
BDA	29	181	0.00	435	0	0.00	37	18	0.38	47	8	0.05	60	18	0.11	42	13	0.16
LEO	181	29	0.00	435	0	0.00	55	0	0.00	55	0	0.00	78	0	0.00	51	4	0.01
CMAES	52	158	0.05	85	350	0.00	0	55	0.00	6	49	0.03	29	49	0.47	43	12	0.13
COA	0	210	0.00	416	19	0.00	37	18	0.38	41	14	0.19	45	33	0.68	0	55	0.00
CPO	0	210	0.00	196	239	0.65	14	41	0.19	13	42	0.16	0	78	0.00	0	55	0.00
FOX	19	191	0.00	82	353	0.00	32	23	0.70	21	34	0.56	41	37	0.91	17	38	0.32
HO	0	210	0.00	287	148	0.14	15	40	0.23	7	48	0.04	9	69	0.02	0	55	0.00
IAGA	14	196	0.00	193	242	0.61	16	39	0.28	8	47	0.05	26	52	0.34	22	33	0.63
SHOA	0	210	0.00	372	63	0.00	15	40	0.23	14	41	0.19	8	70	0.01	0	55	0.00
IFOX	0	210	0.00	53	382	0.00	11	44	0.11	6	49	0.03	24	54	0.27	0	55	0.00
IPSO	15	195	0.00	417	18	0.00	37	18	0.38	46	9	0.06	45	33	0.68	33	22	0.63
IWOA	0	210	0.00	399	36	0.00	45	10	0.08	40	15	0.23	55	23	0.23	39	16	0.28
LSHADE	0	210	0.00	17	418	0.00	0	55	0.00	0	55	0.00	0	78	0.00	0	55	0.00
NRO	0	210	0.00	45	390	0.00	5	50	0.02	0	55	0.00	0	78	0.00	0	55	0.00
VWGWO	0	210	0.00	282	153	0.17	21	34	0.56	3	52	0.01	13	65	0.04	0	55	0.00

Table 11: Wilcoxon signed-rank test results comparing BDA with other optimization algorithms across CL, C17, C19, C21, C22, and RWPs.

Wilcoxon signed-rank test of BDA versus optimization algorithms																		
**Algorithm**	**CL**			**C17**			**C19**			**C21**			**C22**			**RWPs**		
	**R^+**	**R^-**	**P-val**	**R^+**	**R^-**	**P-val**	**R^+**	**R^-**	**P-val**	**R^+**	**R^-**	**P-val**	**R^+**	**R^-**	**P-val**	**R^+**	**R^-**	**P-val**
ALSHADE	29	142	0.01	0	435	0.00	0	55	0.00	0	55	0.00	3	75	0.00	0	45	0.00
BBOA	181	29	0.00	0	435	0.00	18	37	0.38	8	47	0.05	18	60	0.11	13	42	0.16
LEO	190	0	0.00	435	0	0.00	55	0	0.00	55	0	0.00	78	0	0.00	45	0	0.00
CMAES	63	90	0.52	8	427	0.00	0	55	0.00	0	55	0.00	14	64	0.05	24	21	0.91
COA	0	153	0.00	225	210	0.88	29	26	0.92	22	33	0.63	26	52	0.34	0	45	0.00
CPO	13	140	0.00	0	435	0.00	23	32	0.70	0	55	0.00	0	78	0.00	0	45	0.00
FOX	29	124	0.02	14	421	0.00	16	39	0.28	3	52	0.01	39	39	1.00	4	51	0.01
HO	0	153	0.00	0	435	0.00	0	55	0.00	0	55	0.00	0	78	0.00	0	45	0.00
IAGA	174	36	0.01	18	417	0.00	18	37	0.38	0	55	0.00	27	51	0.38	4	51	0.01
SHOA	20	133	0.01	17	418	0.00	23	32	0.70	0	55	0.00	10	68	0.02	0	45	0.00
IFOX	0	153	0.00	0	435	0.00	0	55	0.00	0	55	0.00	4	74	0.00	0	45	0.00
IPSO	47	106	0.16	50	385	0.00	34	21	0.56	15	40	0.23	47	31	0.57	0	45	0.00
IWOA	17	136	0.00	47	388	0.00	30	25	0.85	6	49	0.03	49	29	0.47	6	39	0.05
LSHADE	3	150	0.00	0	435	0.00	0	55	0.00	0	55	0.00	0	78	0.00	0	45	0.00
NRO	0	153	0.00	0	435	0.00	0	55	0.00	0	55	0.00	0	78	0.00	1	54	0.00
VWGWO	0	153	0.00	0	435	0.00	6	49	0.03	0	55	0.00	9	69	0.02	0	45	0.00

Table 12: Wilcoxon signed-rank test results comparing LEO with other optimization algorithms across CL, C17, C19, C21, C22, and RWPs.

Wilcoxon signed-rank test of LEO versus optimization algorithms																		
**Algorithm**	**CL**			**C17**			**C19**			**C21**			**C22**			**RWPs**		
	R^+	R^-	**P-val**	R^+	R^-	**P-val**	R^+	R^-	**P-val**	R^+	R^-	**P-val**	R^+	R^-	**P-val**	R^+	R^-	**P-val**
ALSHADE	0	190	0.00	0	435	0.00	0	55	0.00	0	55	0.00	0	78	0.00	0	45	0.00
BBOA	29	181	0.00	0	435	0.00	0	55	0.00	0	55	0.00	0	78	0.00	4	51	0.01
BDA	0	190	0.00	0	435	0.00	0	55	0.00	0	55	0.00	0	78	0.00	0	45	0.00
CMAES	8	182	0.00	0	435	0.00	0	55	0.00	0	55	0.00	3	75	0.00	6	39	0.05
COA	0	190	0.00	0	435	0.00	0	55	0.00	0	55	0.00	0	78	0.00	0	45	0.00
CPO	0	190	0.00	0	435	0.00	0	55	0.00	0	55	0.00	0	78	0.00	0	45	0.00
FOX	0	190	0.00	2	433	0.00	0	55	0.00	1	54	0.00	0	78	0.00	4	51	0.01
HO	0	190	0.00	0	435	0.00	0	55	0.00	0	55	0.00	0	78	0.00	0	45	0.00
IAGA	28	182	0.00	0	435	0.00	0	55	0.00	0	55	0.00	0	78	0.00	4	51	0.01
SHOA	18	172	0.00	0	435	0.00	0	55	0.00	0	55	0.00	0	78	0.00	0	45	0.00
IFOX	0	190	0.00	0	435	0.00	0	55	0.00	0	55	0.00	0	78	0.00	0	45	0.00
IPSO	0	190	0.00	0	435	0.00	0	55	0.00	0	55	0.00	0	78	0.00	0	45	0.00
IWOA	0	190	0.00	0	435	0.00	6	49	0.03	0	55	0.00	0	78	0.00	0	45	0.00
LSHADE	0	190	0.00	0	435	0.00	0	55	0.00	0	55	0.00	0	78	0.00	0	45	0.00
NRO	0	190	0.00	0	435	0.00	0	55	0.00	0	55	0.00	0	78	0.00	1	54	0.00
VWGWO	0	190	0.00	0	435	0.00	0	55	0.00	0	55	0.00	0	78	0.00	0	45	0.00

Table 13: Wilcoxon signed-rank test results comparing CMAES with other optimization algorithms across CL, C17, C19, C21, C22, and RWPs.

Wilcoxon signed-rank test of CMAES versus optimization algorithms																		
**Algorithm**	**CL**			**C17**			**C19**			**C21**			**C22**			**RWPs**		
	R^+	R^-	**P-val**	R^+	R^-	**P-val**	R^+	R^-	**P-val**	R^+	R^-	**P-val**	R^+	R^-	**P-val**	R^+	R^-	**P-val**
ALSHADE	98	73	0.59	202	233	0.75	45	0	0.00	42	13	0.16	27	51	0.38	0	45	0.00
BBOA	158	52	0.05	350	85	0.00	55	0	0.00	49	6	0.03	49	29	0.47	12	43	0.13
BDA	90	63	0.52	427	8	0.00	55	0	0.00	55	0	0.00	64	14	0.05	21	24	0.91
LEO	182	8	0.00	435	0	0.00	55	0	0.00	55	0	0.00	75	3	0.00	39	6	0.05
COA	72	64	0.84	428	7	0.00	45	0	0.00	51	4	0.01	54	24	0.27	0	45	0.00
CPO	88	65	0.59	388	47	0.00	55	0	0.00	49	6	0.03	36	42	0.85	0	45	0.00
FOX	68	68	1.00	391	44	0.00	45	0	0.00	50	5	0.02	71	7	0.01	12	43	0.13
HO	68	68	1.00	411	24	0.00	45	0	0.00	49	6	0.03	39	39	1.00	0	45	0.00
IAGA	151	59	0.09	348	87	0.00	53	2	0.01	49	6	0.03	59	19	0.13	16	39	0.28
SHOA	68	68	1.00	415	20	0.00	55	0	0.00	49	6	0.03	46	32	0.62	0	45	0.00
IFOX	64	72	0.84	235	200	0.72	45	0	0.00	48	7	0.04	46	32	0.62	1	44	0.01
IPSO	78	75	0.94	428	7	0.00	55	0	0.00	52	3	0.01	72	6	0.01	6	39	0.05
IWOA	58	78	0.61	428	7	0.00	55	0	0.00	51	4	0.01	73	5	0.00	10	35	0.16
LSHADE	8	70	0.02	71	364	0.00	43	2	0.01	41	14	0.19	5	50	0.02	0	45	0.00
NRO	32	59	0.35	239	196	0.65	32	4	0.05	41	14	0.19	19	47	0.24	2	53	0.01
VWGWO	85	68	0.69	399	36	0.00	55	0	0.00	49	6	0.03	45	33	0.68	0	45	0.00

Table 14: Wilcoxon signed-rank test results comparing COA with other optimization algorithms across CL, C17, C19, C21, C22, and RWPs.

Wilcoxon signed-rank test of COA versus optimization algorithms																		
**Algorithm**	**CL**			**C17**			**C19**			**C21**			**C22**			**RWPs**		
	R^+	R^-	**P-val**	R^+	R^-	**P-val**	R^+	R^-	**P-val**	R^+	R^-	**P-val**	R^+	R^-	**P-val**	R^+	R^-	**P-val**
ALSHADE	38	115	0.07	0	435	0.00	10	35	0.16	0	55	0.00	0	78	0.00	10	35	0.16
BBOA	210	0	0.00	19	416	0.00	18	37	0.38	14	41	0.19	33	45	0.68	55	0	0.00
BDA	153	0	0.00	210	225	0.88	26	29	0.92	33	22	0.63	52	26	0.34	45	0	0.00
LEO	190	0	0.00	435	0	0.00	55	0	0.00	55	0	0.00	78	0	0.00	45	0	0.00
CMAES	64	72	0.84	7	428	0.00	0	45	0.00	4	51	0.01	24	54	0.27	45	0	0.00
CPO	56	64	0.82	40	395	0.00	23	32	0.70	0	55	0.00	3	75	0.00	10	35	0.16
FOX	60	18	0.10	13	422	0.00	24	21	0.91	21	34	0.56	41	37	0.91	55	0	0.00
HO	0	66	0.00	0	435	0.00	0	45	0.00	0	55	0.00	0	78	0.00	28	17	0.57
IAGA	210	0	0.00	19	416	0.00	18	37	0.38	15	40	0.23	32	46	0.62	55	0	0.00
SHOA	59	61	0.95	55	380	0.00	29	26	0.92	10	45	0.08	3	75	0.00	1	44	0.01
IFOX	3	75	0.00	0	435	0.00	0	45	0.00	0	55	0.00	13	65	0.04	24	21	0.91
IPSO	87	49	0.33	150	285	0.15	39	16	0.28	31	24	0.77	50	28	0.42	44	1	0.01
IWOA	32	104	0.06	87	348	0.00	26	29	0.92	8	47	0.05	62	16	0.08	44	1	0.01
LSHADE	18	87	0.03	0	435	0.00	14	41	0.19	0	55	0.00	0	78	0.00	10	35	0.16
NRO	14	77	0.03	0	435	0.00	4	41	0.03	0	55	0.00	0	78	0.00	14	41	0.19
VWGWO	78	42	0.31	0	435	0.00	23	32	0.70	0	55	0.00	12	66	0.03	12	33	0.25

Table 15: Wilcoxon signed-rank test results comparing CPO with other optimization algorithms across CL, C17, C19, C21, C22, and RWPs.

Wilcoxon signed-rank test of CPO versus optimization algorithms																		
**Algorithm**	**CL**			**C17**			**C19**			**C21**			**C22**			**RWPs**		
	R^+	R^-	**P-val**	R^+	R^-	**P-val**	R^+	R^-	**P-val**	R^+	R^-	**P-val**	R^+	R^-	**P-val**	R^+	R^-	**P-val**
ALSHADE	33	103	0.07	76	359	0.00	13	42	0.16	2	53	0.01	17	61	0.09	9	36	0.13
BBOA	210	0	0.00	239	196	0.65	41	14	0.19	42	13	0.16	78	0	0.00	55	0	0.00
BDA	140	13	0.00	435	0	0.00	32	23	0.70	55	0	0.00	78	0	0.00	45	0	0.00
LEO	190	0	0.00	435	0	0.00	55	0	0.00	55	0	0.00	78	0	0.00	45	0	0.00
CMAES	65	88	0.59	47	388	0.00	0	55	0.00	6	49	0.03	42	36	0.85	45	0	0.00
COA	64	56	0.82	395	40	0.00	32	23	0.70	55	0	0.00	75	3	0.00	35	10	0.16
FOX	68	68	1.00	167	268	0.28	36	19	0.43	42	13	0.16	78	0	0.00	54	1	0.00
HO	17	103	0.01	252	183	0.47	19	36	0.43	28	27	1.00	44	34	0.73	32	13	0.30
IAGA	210	0	0.00	225	210	0.88	41	14	0.19	42	13	0.16	71	7	0.01	55	0	0.00
SHOA	50	55	0.88	435	0	0.00	48	7	0.04	43	12	0.13	69	9	0.02	20	25	0.82
IFOX	25	111	0.03	36	399	0.00	12	43	0.13	16	39	0.28	66	12	0.03	44	1	0.01
IPSO	83	53	0.44	431	4	0.00	42	13	0.16	55	0	0.00	78	0	0.00	44	1	0.01
IWOA	24	96	0.04	408	27	0.00	48	7	0.04	55	0	0.00	66	12	0.03	44	1	0.01
LSHADE	0	105	0.00	2	433	0.00	0	55	0.00	0	55	0.00	7	71	0.01	17	28	0.57
NRO	0	105	0.00	28	407	0.00	3	52	0.01	0	55	0.00	0	78	0.00	28	27	1.00
VWGWO	93	43	0.20	221	214	0.95	30	25	0.85	40	15	0.23	73	5	0.00	39	6	0.05

Table 16: Wilcoxon signed-rank test results comparing FOX with other optimization algorithms across CL, C17, C19, C21, C22, and RWPs.

Wilcoxon signed-rank test of FOX versus optimization algorithms																		
**Algorithm**	**CL**			**C17**			**C19**			**C21**			**C22**			**RWPs**		
	R^+	R^-	**P-val**	R^+	R^-	**P-val**	R^+	R^-	**P-val**	R^+	R^-	**P-val**	R^+	R^-	**P-val**	R^+	R^-	**P-val**
ALSHADE	45	126	0.08	41	394	0.00	11	34	0.20	2	53	0.01	0	78	0.00	1	54	0.00
BBOA	191	19	0.00	353	82	0.00	23	32	0.70	34	21	0.56	37	41	0.91	38	17	0.32
BDA	124	29	0.02	421	14	0.00	39	16	0.28	52	3	0.01	39	39	1.00	51	4	0.01
LEO	190	0	0.00	433	2	0.00	55	0	0.00	54	1	0.00	78	0	0.00	51	4	0.01
CMAES	68	68	1.00	44	391	0.00	0	45	0.00	5	50	0.02	7	71	0.01	43	12	0.13
COA	18	60	0.10	422	13	0.00	21	24	0.91	34	21	0.56	37	41	0.91	0	55	0.00
CPO	68	68	1.00	268	167	0.28	19	36	0.43	13	42	0.16	0	78	0.00	1	54	0.00
HO	2	76	0.00	374	61	0.00	13	32	0.30	5	50	0.02	4	74	0.00	14	41	0.19
IAGA	189	21	0.00	309	126	0.05	22	33	0.63	33	22	0.63	41	37	0.91	38	17	0.32
SHOA	70	66	0.92	406	29	0.00	24	31	0.77	14	41	0.19	23	55	0.23	1	54	0.00
IFOX	0	66	0.00	6	429	0.00	0	45	0.00	0	55	0.00	16	62	0.08	7	48	0.04
IPSO	56	97	0.33	424	11	0.00	34	21	0.56	50	5	0.02	59	19	0.13	39	16	0.28
IWOA	31	122	0.03	424	11	0.00	34	21	0.56	28	27	1.00	43	35	0.79	47	8	0.05
LSHADE	22	98	0.03	0	435	0.00	16	39	0.28	2	53	0.01	0	78	0.00	0	55	0.00
NRO	17	88	0.03	49	386	0.00	3	42	0.02	0	55	0.00	0	78	0.00	0	55	0.00
VWGWO	73	47	0.46	381	54	0.00	26	29	0.92	17	38	0.32	6	72	0.01	1	54	0.00

Table 17: Wilcoxon signed-rank test results comparing HO with other optimization algorithms across CL, C17, C19, C21, C22, and RWPs.

Wilcoxon signed-rank test of HO versus optimization algorithms																		
**Algorithm**	**CL**			**C17**			**C19**			**C21**			**C22**			**RWPs**		
	R^+	R^-	**P-val**	R^+	R^-	**P-val**	R^+	R^-	**P-val**	R^+	R^-	**P-val**	R^+	R^-	**P-val**	R^+	R^-	**P-val**
ALSHADE	43	110	0.11	11	424	0.00	22	23	1.00	3	52	0.01	11	67	0.03	10	35	0.16
BBOA	210	0	0.00	148	287	0.14	40	15	0.23	48	7	0.04	69	9	0.02	55	0	0.00
BDA	153	0	0.00	435	0	0.00	55	0	0.00	55	0	0.00	78	0	0.00	45	0	0.00
LEO	190	0	0.00	435	0	0.00	55	0	0.00	55	0	0.00	78	0	0.00	45	0	0.00
CMAES	68	68	1.00	24	411	0.00	0	45	0.00	6	49	0.03	39	39	1.00	45	0	0.00
COA	66	0	0.00	435	0	0.00	45	0	0.00	55	0	0.00	78	0	0.00	17	28	0.57
CPO	103	17	0.01	183	252	0.47	36	19	0.43	27	28	1.00	34	44	0.73	13	32	0.30
FOX	76	2	0.00	61	374	0.00	32	13	0.30	50	5	0.02	74	4	0.00	41	14	0.19
IAGA	210	0	0.00	118	317	0.03	36	19	0.43	43	12	0.13	67	11	0.03	52	3	0.01
SHOA	71	49	0.53	364	71	0.00	37	18	0.38	37	18	0.38	45	33	0.68	7	38	0.07
IFOX	27	51	0.35	11	424	0.00	21	24	0.91	16	39	0.28	49	29	0.47	25	20	0.82
IPSO	92	44	0.21	435	0	0.00	55	0	0.00	55	0	0.00	78	0	0.00	44	1	0.01
IWOA	44	92	0.21	435	0	0.00	55	0	0.00	48	7	0.04	78	0	0.00	44	1	0.01
LSHADE	19	86	0.04	4	431	0.00	27	28	1.00	3	52	0.01	6	72	0.01	10	35	0.16
NRO	15	76	0.03	5	430	0.00	16	29	0.50	0	55	0.00	0	78	0.00	15	40	0.23
VWGWO	120	0	0.00	128	307	0.05	40	15	0.23	29	26	0.92	56	22	0.20	22	23	1.00

Table 18: Wilcoxon signed-rank test results comparing IAGA with other optimization algorithms across CL, C17, C19, C21, C22, and RWPs.

Wilcoxon signed-rank test of IAGA versus optimization algorithms																		
**Algorithm**	**CL**			**C17**			**C19**			**C21**			**C22**			**RWPs**		
	**R^+**	**R^-**	**P-val**	**R^+**	**R^-**	**P-val**	**R^+**	**R^-**	**P-val**	**R^+**	**R^-**	**P-val**	**R^+**	**R^-**	**P-val**	**R^+**	**R^-**	**P-val**
ALSHADE	7	203	0.00	45	390	0.00	9	46	0.06	3	52	0.01	0	78	0.00	0	55	0.00
BBOA	196	14	0.00	242	193	0.61	39	16	0.28	47	8	0.05	52	26	0.34	33	22	0.63
BDA	36	174	0.01	417	18	0.00	37	18	0.38	55	0	0.00	51	27	0.38	51	4	0.01
LEO	182	28	0.00	435	0	0.00	55	0	0.00	55	0	0.00	78	0	0.00	51	4	0.01
CMAES	59	151	0.09	87	348	0.00	2	53	0.01	6	49	0.03	19	59	0.13	39	16	0.28
COA	0	210	0.00	416	19	0.00	37	18	0.38	40	15	0.23	46	32	0.62	0	55	0.00
CPO	0	210	0.00	210	225	0.88	14	41	0.19	13	42	0.16	7	71	0.01	0	55	0.00
FOX	21	189	0.00	126	309	0.05	33	22	0.63	22	33	0.63	37	41	0.91	17	38	0.32
HO	0	210	0.00	317	118	0.03	19	36	0.43	12	43	0.13	11	67	0.03	3	52	0.01
SHOA	0	210	0.00	376	59	0.00	17	38	0.32	14	41	0.19	20	58	0.15	0	55	0.00
IFOX	0	210	0.00	52	383	0.00	13	42	0.16	8	47	0.05	21	57	0.18	0	55	0.00
IPSO	26	184	0.00	416	19	0.00	37	18	0.38	41	14	0.19	66	12	0.03	35	20	0.49
IWOA	5	205	0.00	399	36	0.00	45	10	0.08	39	16	0.28	58	20	0.15	41	14	0.19
LSHADE	0	210	0.00	19	416	0.00	6	49	0.03	0	55	0.00	0	78	0.00	0	55	0.00
NRO	0	210	0.00	45	390	0.00	6	49	0.03	0	55	0.00	3	75	0.00	0	55	0.00
VWGWO	2	208	0.00	316	119	0.03	26	29	0.92	21	34	0.56	7	71	0.01	0	55	0.00

Table 19: Wilcoxon signed-rank test results comparing SHOA with other optimization algorithms across CL, C17, C19, C21, C22, and RWPs.

Wilcoxon signed-rank test of SHOA versus optimization algorithms																		
**Algorithm**	**CL**			**C17**			**C19**			**C21**			**C22**			**RWPs**		
	**R^+**	**R^-**	**P-val**	**R^+**	**R^-**	**P-val**	**R^+**	**R^-**	**P-val**	**R^+**	**R^-**	**P-val**	**R^+**	**R^-**	**P-val**	**R^+**	**R^-**	**P-val**
ALSHADE	69	67	0.96	0	435	0.00	12	43	0.13	3	52	0.01	14	64	0.05	15	30	0.43
BBOA	210	0	0.00	63	372	0.00	40	15	0.23	41	14	0.19	70	8	0.01	55	0	0.00
BDA	133	20	0.01	418	17	0.00	32	23	0.70	55	0	0.00	68	10	0.02	45	0	0.00
LEO	172	18	0.00	435	0	0.00	55	0	0.00	55	0	0.00	78	0	0.00	45	0	0.00
CMAES	68	68	1.00	20	415	0.00	0	55	0.00	6	49	0.03	32	46	0.62	45	0	0.00
COA	61	59	0.95	380	55	0.00	26	29	0.92	45	10	0.08	75	3	0.00	44	1	0.01
CPO	55	50	0.88	0	435	0.00	7	48	0.04	12	43	0.13	9	69	0.02	25	20	0.82
FOX	66	70	0.92	29	406	0.00	31	24	0.77	41	14	0.19	55	23	0.23	54	1	0.00
HO	49	71	0.53	71	364	0.00	18	37	0.38	18	37	0.38	33	45	0.68	38	7	0.07
IAGA	210	0	0.00	59	376	0.00	38	17	0.32	41	14	0.19	58	20	0.15	55	0	0.00
IFOX	56	80	0.53	5	430	0.00	12	43	0.13	5	50	0.02	33	45	0.68	38	7	0.07
IPSO	87	49	0.33	411	24	0.00	27	28	1.00	53	2	0.01	56	22	0.20	44	1	0.01
IWOA	48	57	0.78	346	89	0.00	35	20	0.49	37	18	0.38	66	12	0.03	44	1	0.01
LSHADE	6	72	0.01	0	435	0.00	0	55	0.00	1	54	0.00	5	73	0.00	17	28	0.57
NRO	5	61	0.01	4	431	0.00	0	55	0.00	0	55	0.00	0	78	0.00	34	21	0.56
VWGWO	76	60	0.68	47	388	0.00	27	28	1.00	33	22	0.63	41	37	0.91	33	12	0.25

Table 20: Wilcoxon signed-rank test results comparing IPSO with other optimization algorithms across CL, C17, C19, C21, C22, and RWPs.

Wilcoxon signed-rank test of IPSO versus optimization algorithms																		
**Algorithm**	**CL**			**C17**			**C19**			**C21**			**C22**			**RWPs**		
	R^+	R^-	**P-val**	R^+	R^-	**P-val**	R^+	R^-	**P-val**	R^+	R^-	**P-val**	R^+	R^-	**P-val**	R^+	R^-	**P-val**
ALSHADE	40	113	0.08	0	435	0.00	5	50	0.02	0	55	0.00	0	78	0.00	1	44	0.01
BBOA	195	15	0.00	18	417	0.00	18	37	0.38	9	46	0.06	33	45	0.68	22	33	0.63
BDA	106	47	0.16	385	50	0.00	21	34	0.56	40	15	0.23	31	47	0.57	45	0	0.00
LEO	190	0	0.00	435	0	0.00	55	0	0.00	55	0	0.00	78	0	0.00	45	0	0.00
CMAES	75	78	0.94	7	428	0.00	0	55	0.00	3	52	0.01	6	72	0.01	39	6	0.05
COA	49	87	0.33	285	150	0.15	16	39	0.28	24	31	0.77	28	50	0.42	1	44	0.01
CPO	53	83	0.44	4	431	0.00	13	42	0.16	0	55	0.00	0	78	0.00	1	44	0.01
FOX	97	56	0.33	11	424	0.00	21	34	0.56	5	50	0.02	19	59	0.13	16	39	0.28
HO	44	92	0.21	0	435	0.00	0	55	0.00	0	55	0.00	0	78	0.00	1	44	0.01
IAGA	184	26	0.00	19	416	0.00	18	37	0.38	14	41	0.19	12	66	0.03	20	35	0.49
SHOA	49	87	0.33	24	411	0.00	28	27	1.00	2	53	0.01	22	56	0.20	1	44	0.01
IFOX	42	111	0.10	0	435	0.00	0	55	0.00	0	55	0.00	0	78	0.00	1	44	0.01
IWOA	21	99	0.03	176	259	0.38	16	39	0.28	6	49	0.03	45	33	0.68	35	1	0.02
LSHADE	12	108	0.01	0	435	0.00	0	55	0.00	0	55	0.00	0	78	0.00	1	44	0.01
NRO	14	106	0.01	0	435	0.00	0	55	0.00	0	55	0.00	0	78	0.00	3	52	0.01
VWGWO	73	80	0.87	0	435	0.00	5	50	0.02	0	55	0.00	0	78	0.00	1	44	0.01

Table 21: Wilcoxon signed-rank test results comparing IWOA with other optimization algorithms across CL, C17, C19, C21, C22, and RWPs.

Wilcoxon signed-rank test of IWOA versus optimization algorithms																		
**Algorithm**	**CL**			**C17**			**C19**			**C21**			**C22**			**RWPs**		
	R^+	R^-	**P-val**	R^+	R^-	**P-val**	R^+	R^-	**P-val**	R^+	R^-	**P-val**	R^+	R^-	**P-val**	R^+	R^-	**P-val**
ALSHADE	65	71	0.88	0	435	0.00	5	50	0.02	0	55	0.00	0	78	0.00	1	44	0.01
BBOA	210	0	0.00	36	399	0.00	10	45	0.08	15	40	0.23	23	55	0.23	16	39	0.28
BDA	136	17	0.00	388	47	0.00	25	30	0.85	49	6	0.03	29	49	0.47	39	6	0.05
LEO	190	0	0.00	435	0	0.00	49	6	0.03	55	0	0.00	78	0	0.00	45	0	0.00
CMAES	78	58	0.61	7	428	0.00	0	55	0.00	4	51	0.01	5	73	0.00	35	10	0.16
COA	104	32	0.06	348	87	0.00	29	26	0.92	47	8	0.05	16	62	0.08	1	44	0.01
CPO	96	24	0.04	27	408	0.00	7	48	0.04	0	55	0.00	12	66	0.03	1	44	0.01
FOX	122	31	0.03	11	424	0.00	21	34	0.56	27	28	1.00	35	43	0.79	8	47	0.05
HO	92	44	0.21	0	435	0.00	0	55	0.00	7	48	0.04	0	78	0.00	1	44	0.01
IAGA	205	5	0.00	36	399	0.00	10	45	0.08	16	39	0.28	20	58	0.15	14	41	0.19
SHOA	57	48	0.78	89	346	0.00	20	35	0.49	18	37	0.38	12	66	0.03	1	44	0.01
IFOX	97	56	0.33	0	435	0.00	0	55	0.00	7	48	0.04	12	66	0.03	1	44	0.01
IPSO	99	21	0.03	259	176	0.38	39	16	0.28	49	6	0.03	33	45	0.68	1	35	0.02
LSHADE	14	64	0.05	0	435	0.00	0	55	0.00	0	55	0.00	0	78	0.00	1	44	0.01
NRO	16	62	0.07	0	435	0.00	0	55	0.00	0	55	0.00	0	78	0.00	3	52	0.01
VWGWO	129	24	0.01	0	435	0.00	5	50	0.02	7	48	0.04	18	60	0.11	1	44	0.01

Table 22: Wilcoxon signed-rank test results comparing LSHADE with other optimization algorithms across CL, C17, C19, C21, C22, and RWPs.

Wilcoxon signed-rank test of LSHADE versus optimization algorithms																		
**Algorithm**	**CL**			**C17**			**C19**			**C21**			**C22**			**RWPs**		
	**R^+**	**R^-**	**P-val**	**R^+**	**R^-**	**P-val**	**R^+**	**R^-**	**P-val**	**R^+**	**R^-**	**P-val**	**R^+**	**R^-**	**P-val**	**R^+**	**R^-**	**P-val**
ALSHADE	136	0	0.00	415	20	0.00	36	19	0.43	26	29	0.92	60	18	0.11	37	8	0.10
BBOA	210	0	0.00	418	17	0.00	55	0	0.00	55	0	0.00	78	0	0.00	55	0	0.00
BDA	150	3	0.00	435	0	0.00	55	0	0.00	55	0	0.00	78	0	0.00	45	0	0.00
LEO	190	0	0.00	435	0	0.00	55	0	0.00	55	0	0.00	78	0	0.00	45	0	0.00
CMAES	70	8	0.02	364	71	0.00	2	43	0.01	14	41	0.19	50	5	0.02	45	0	0.00
COA	87	18	0.03	435	0	0.00	41	14	0.19	55	0	0.00	78	0	0.00	35	10	0.16
CPO	105	0	0.00	433	2	0.00	55	0	0.00	55	0	0.00	71	7	0.01	28	17	0.57
FOX	98	22	0.03	435	0	0.00	39	16	0.28	53	2	0.01	78	0	0.00	55	0	0.00
HO	86	19	0.04	431	4	0.00	28	27	1.00	52	3	0.01	72	6	0.01	35	10	0.16
IAGA	210	0	0.00	416	19	0.00	49	6	0.03	55	0	0.00	78	0	0.00	55	0	0.00
SHOA	72	6	0.01	435	0	0.00	55	0	0.00	54	1	0.00	73	5	0.00	28	17	0.57
IFOX	96	24	0.04	386	49	0.00	31	24	0.77	50	5	0.02	78	0	0.00	36	9	0.13
IPSO	108	12	0.01	435	0	0.00	55	0	0.00	55	0	0.00	78	0	0.00	44	1	0.01
IWOA	64	14	0.05	435	0	0.00	55	0	0.00	55	0	0.00	78	0	0.00	44	1	0.01
NRO	30	6	0.09	395	40	0.00	19	26	0.73	32	23	0.70	35	31	0.90	27	18	0.65
VWGWO	130	6	0.00	435	0	0.00	55	0	0.00	55	0	0.00	78	0	0.00	37	8	0.10

Table 23: Wilcoxon signed-rank test results comparing NRO with other optimization algorithms across CL, C17, C19, C21, C22, and RWPs.

Wilcoxon signed-rank test of NRO versus optimization algorithms																		
**Algorithm**	**CL**			**C17**			**C19**			**C21**			**C22**			**RWPs**		
	**R^+**	**R^-**	**P-val**	**R^+**	**R^-**	**P-val**	**R^+**	**R^-**	**P-val**	**R^+**	**R^-**	**P-val**	**R^+**	**R^-**	**P-val**	**R^+**	**R^-**	**P-val**
ALSHADE	120	16	0.01	219	216	0.98	37	8	0.10	26	29	0.92	59	19	0.13	21	34	0.56
BBOA	210	0	0.00	390	45	0.00	50	5	0.02	55	0	0.00	78	0	0.00	55	0	0.00
BDA	153	0	0.00	435	0	0.00	55	0	0.00	55	0	0.00	78	0	0.00	54	1	0.00
LEO	190	0	0.00	435	0	0.00	55	0	0.00	55	0	0.00	78	0	0.00	54	1	0.00
CMAES	59	32	0.35	196	239	0.65	4	32	0.05	14	41	0.19	47	19	0.24	53	2	0.01
COA	77	14	0.03	435	0	0.00	41	4	0.03	55	0	0.00	78	0	0.00	41	14	0.19
CPO	105	0	0.00	407	28	0.00	52	3	0.01	55	0	0.00	78	0	0.00	27	28	1.00
FOX	88	17	0.03	386	49	0.00	42	3	0.02	55	0	0.00	78	0	0.00	55	0	0.00
HO	76	15	0.03	430	5	0.00	29	16	0.50	55	0	0.00	78	0	0.00	40	15	0.23
IAGA	210	0	0.00	390	45	0.00	49	6	0.03	55	0	0.00	75	3	0.00	55	0	0.00
SHOA	61	5	0.01	431	4	0.00	55	0	0.00	55	0	0.00	78	0	0.00	21	34	0.56
IFOX	86	19	0.04	272	163	0.25	33	12	0.25	55	0	0.00	78	0	0.00	52	3	0.01
IPSO	106	14	0.01	435	0	0.00	55	0	0.00	55	0	0.00	78	0	0.00	52	3	0.01
IWOA	62	16	0.07	435	0	0.00	55	0	0.00	55	0	0.00	78	0	0.00	52	3	0.01
LSHADE	6	30	0.09	40	395	0.00	26	19	0.73	23	32	0.70	31	35	0.90	18	27	0.65
VWGWO	119	1	0.00	427	8	0.00	55	0	0.00	55	0	0.00	78	0	0.00	45	10	0.08

Table 24: Wilcoxon signed-rank test results comparing VWGWO with other optimization algorithms across CL, C17, C19, C21, C22, and RWPs.

Wilcoxon signed-rank test of VWGWO versus optimization algorithms																		
**Algorithm**	**CL**			**C17**			**C19**			**C21**			**C22**			**RWPs**		
	**R^+**	**R^-**	**P-val**	**R^+**	**R^-**	**P-val**	**R^+**	**R^-**	**P-val**	**R^+**	**R^-**	**P-val**	**R^+**	**R^-**	**P-val**	**R^+**	**R^-**	**P-val**
ALSHADE	33	138	0.02	14	421	0.00	3	52	0.01	0	55	0.00	9	69	0.02	9	36	0.13
BBOA	210	0	0.00	153	282	0.17	34	21	0.56	52	3	0.01	65	13	0.04	55	0	0.00
BDA	153	0	0.00	435	0	0.00	49	6	0.03	55	0	0.00	69	9	0.02	45	0	0.00
LEO	190	0	0.00	435	0	0.00	55	0	0.00	55	0	0.00	78	0	0.00	45	0	0.00
CMAES	68	85	0.69	36	399	0.00	0	55	0.00	6	49	0.03	33	45	0.68	45	0	0.00
COA	42	78	0.31	435	0	0.00	32	23	0.70	55	0	0.00	66	12	0.03	33	12	0.25
CPO	43	93	0.20	214	221	0.95	25	30	0.85	15	40	0.23	5	73	0.00	6	39	0.05
FOX	47	73	0.46	54	381	0.00	29	26	0.92	38	17	0.32	72	6	0.01	54	1	0.00
HO	0	120	0.00	307	128	0.05	15	40	0.23	26	29	0.92	22	56	0.20	23	22	1.00
IAGA	208	2	0.00	119	316	0.03	29	26	0.92	34	21	0.56	71	7	0.01	55	0	0.00
SHOA	60	76	0.68	388	47	0.00	28	27	1.00	22	33	0.63	37	41	0.91	12	33	0.25
IFOX	0	120	0.00	26	409	0.00	10	45	0.08	12	43	0.13	50	28	0.42	21	24	0.91
IPSO	80	73	0.87	435	0	0.00	50	5	0.02	55	0	0.00	78	0	0.00	44	1	0.01
IWOA	24	129	0.01	435	0	0.00	50	5	0.02	48	7	0.04	60	18	0.11	44	1	0.01
LSHADE	6	130	0.00	0	435	0.00	0	55	0.00	0	55	0.00	0	78	0.00	8	37	0.10
NRO	1	119	0.00	8	427	0.00	0	55	0.00	0	55	0.00	0	78	0.00	10	45	0.08
