# Supplementary figures and images for: An improved FOX optimization algorithm using adaptive exploration and exploitation for global optimization
Source: PLoS One. 2025 Sep 18;20(9):e0331965. doi: 10.1371/journal.pone.0331965 (PMC12445531; doi:10.1371/journal.pone.0331965)

C17F25

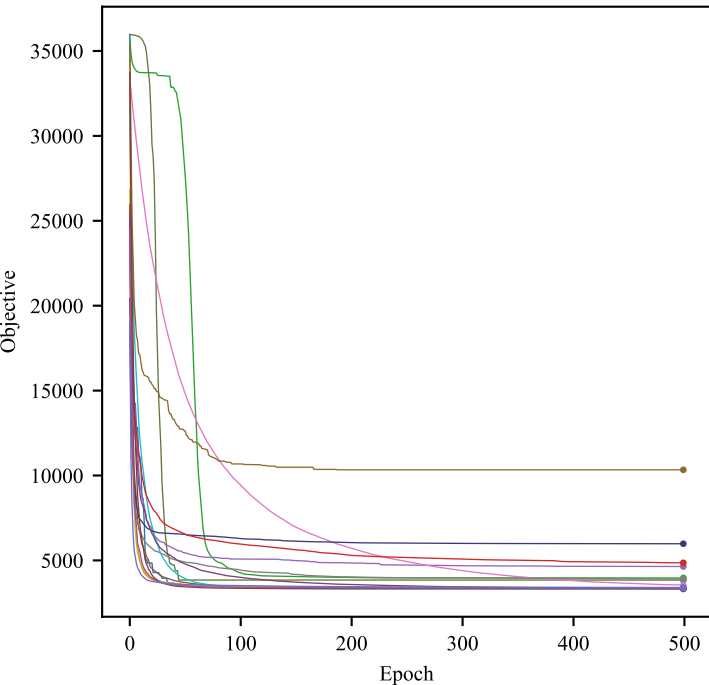

Supplement: S2 File — This compressed file contains all the results of the conducted experiments. Each main directory within the archive includes three subdirectories: Analysis, best_fit, and convergence. The best_fit and convergence folders contain the raw experimental data obtained from 30 independent runs. The Analysis folder includes tables and figures derived from these raw results. These files can be used to reproduce and further analyze the results presented in the manuscript. (ZIP) [file pone.0331965.s002.zip › cec2017/Analysis/PLOTS/C17F25.pdf]

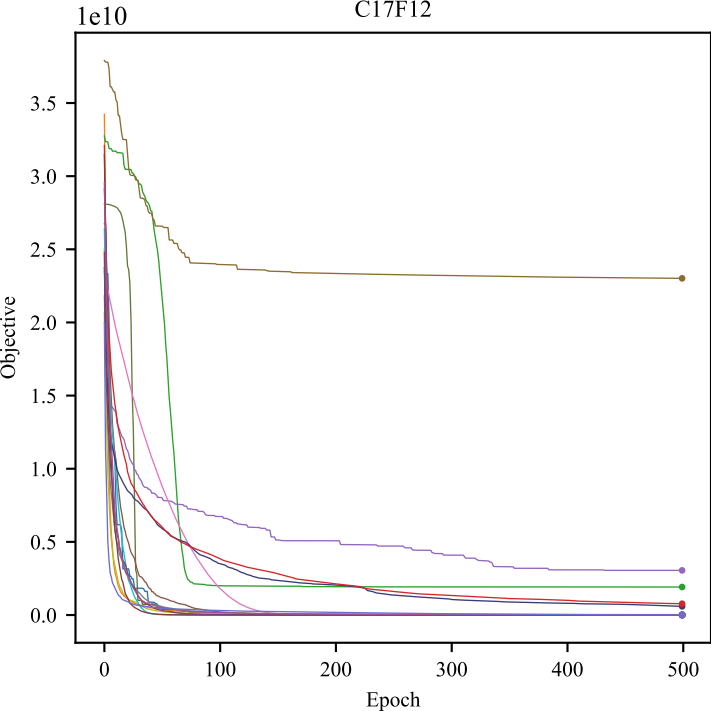

Supplement: S2 File — This compressed file contains all the results of the conducted experiments. Each main directory within the archive includes three subdirectories: Analysis, best_fit, and convergence. The best_fit and convergence folders contain the raw experimental data obtained from 30 independent runs. The Analysis folder includes tables and figures derived from these raw results. These files can be used to reproduce and further analyze the results presented in the manuscript. (ZIP) [file pone.0331965.s002.zip › cec2017/Analysis/PLOTS/C17F12.pdf]

C17F26

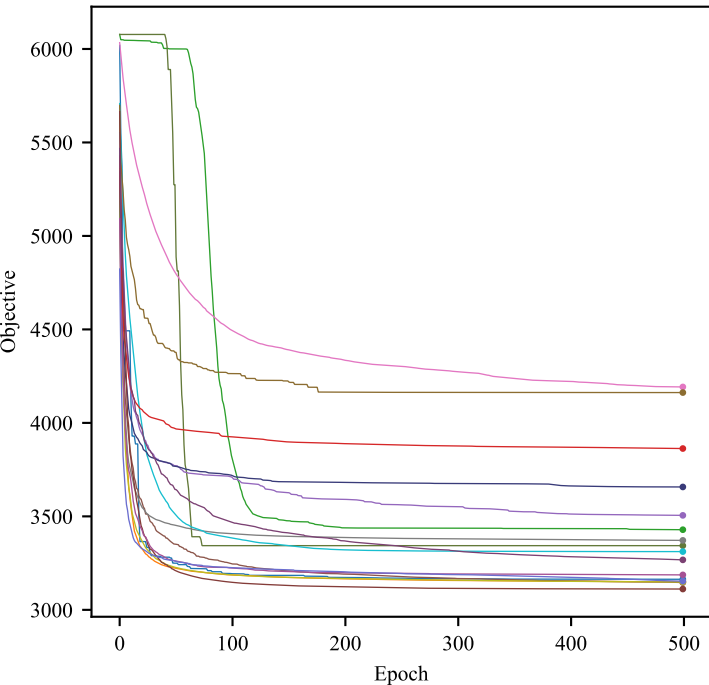

Supplement: S2 File — This compressed file contains all the results of the conducted experiments. Each main directory within the archive includes three subdirectories: Analysis, best_fit, and convergence. The best_fit and convergence folders contain the raw experimental data obtained from 30 independent runs. The Analysis folder includes tables and figures derived from these raw results. These files can be used to reproduce and further analyze the results presented in the manuscript. (ZIP) [file pone.0331965.s002.zip › cec2017/Analysis/PLOTS/C17F26.pdf]

# Friedman Average Ranks

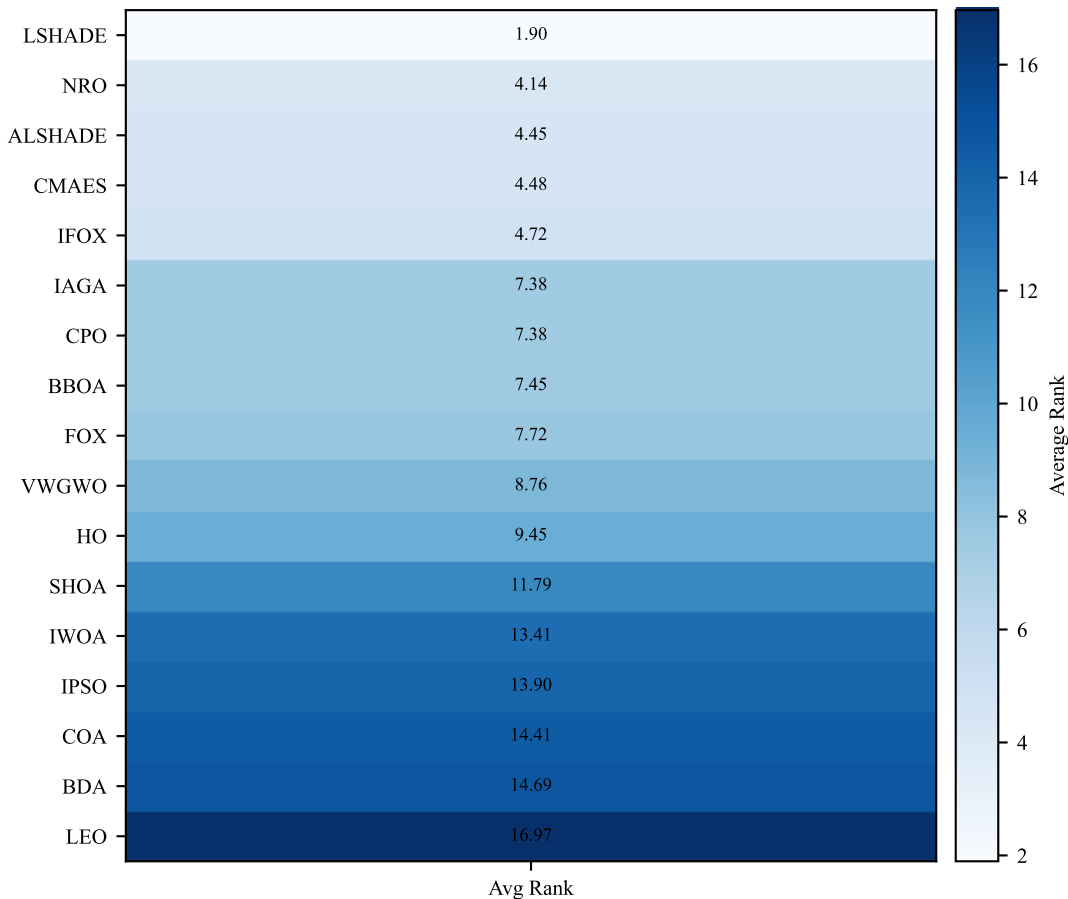

Supplement: S2 File — This compressed file contains all the results of the conducted experiments. Each main directory within the archive includes three subdirectories: Analysis, best_fit, and convergence. The best_fit and convergence folders contain the raw experimental data obtained from 30 independent runs. The Analysis folder includes tables and figures derived from these raw results. These files can be used to reproduce and further analyze the results presented in the manuscript. (ZIP) [file pone.0331965.s002.zip › cec2017/Analysis/PLOTS/non_parametric/friedman_ranking_heatmap_annotated.pdf]

Wilcoxon Two-sided p-values

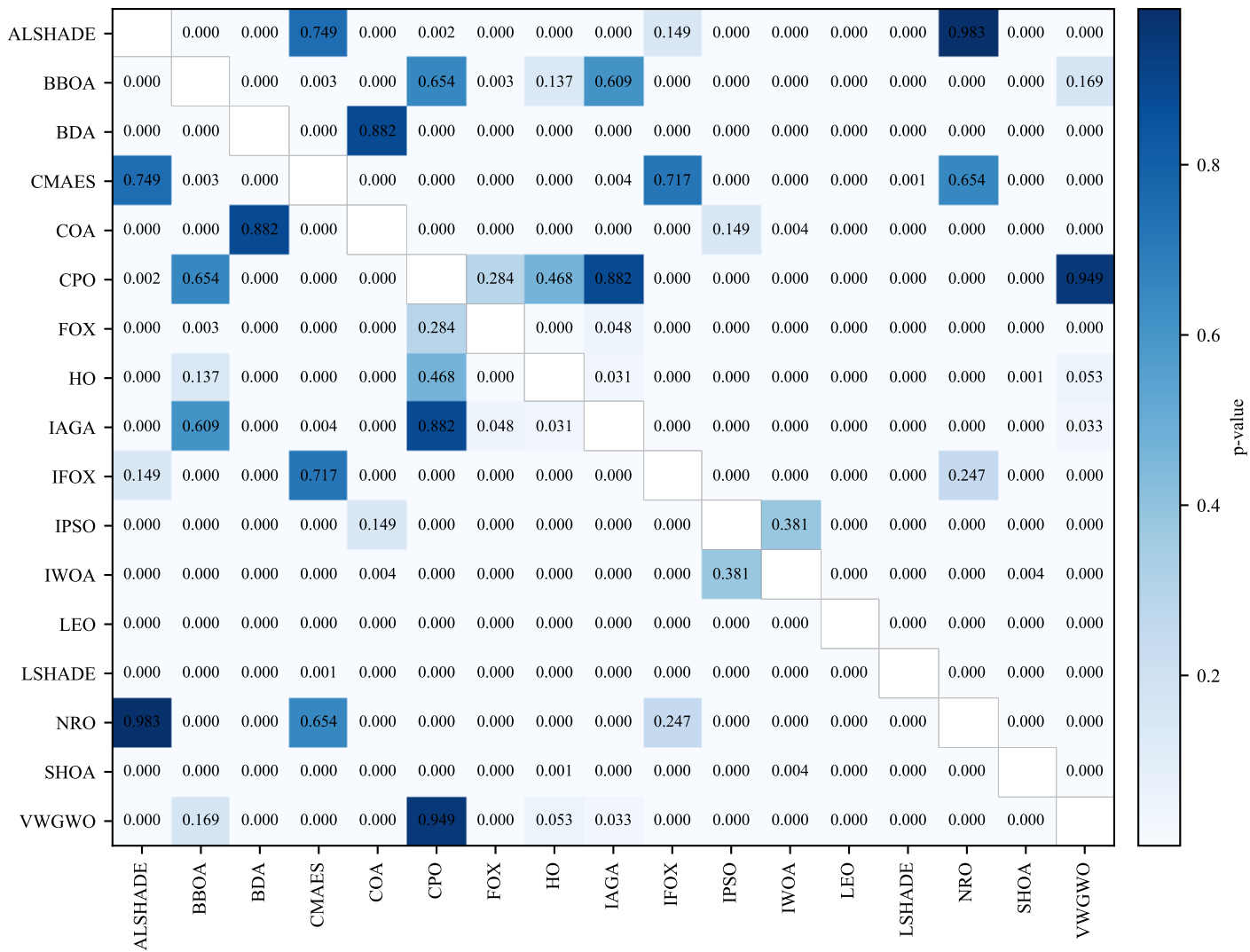

Supplement: S2 File — This compressed file contains all the results of the conducted experiments. Each main directory within the archive includes three subdirectories: Analysis, best_fit, and convergence. The best_fit and convergence folders contain the raw experimental data obtained from 30 independent runs. The Analysis folder includes tables and figures derived from these raw results. These files can be used to reproduce and further analyze the results presented in the manuscript. (ZIP) [file pone.0331965.s002.zip › cec2017/Analysis/PLOTS/non_parametric/wilcoxon_pvalues_heatmap_annotated.pdf]

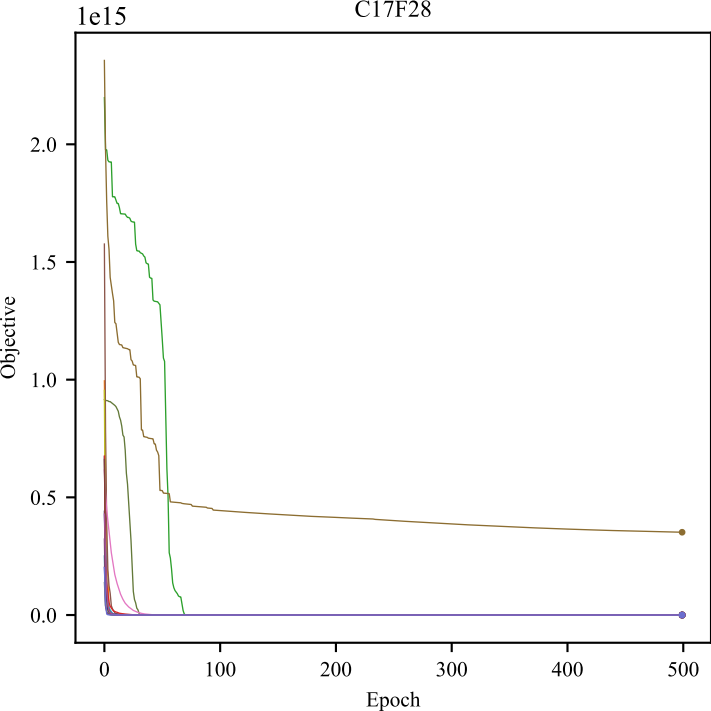

Supplement: S2 File — This compressed file contains all the results of the conducted experiments. Each main directory within the archive includes three subdirectories: Analysis, best_fit, and convergence. The best_fit and convergence folders contain the raw experimental data obtained from 30 independent runs. The Analysis folder includes tables and figures derived from these raw results. These files can be used to reproduce and further analyze the results presented in the manuscript. (ZIP) [file pone.0331965.s002.zip › cec2017/Analysis/PLOTS/C17F28.pdf]

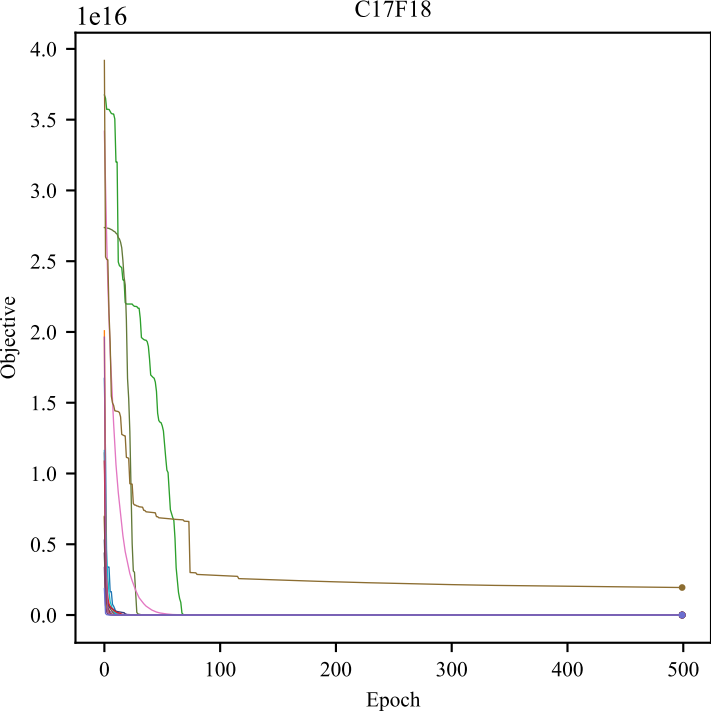

Supplement: S2 File — This compressed file contains all the results of the conducted experiments. Each main directory within the archive includes three subdirectories: Analysis, best_fit, and convergence. The best_fit and convergence folders contain the raw experimental data obtained from 30 independent runs. The Analysis folder includes tables and figures derived from these raw results. These files can be used to reproduce and further analyze the results presented in the manuscript. (ZIP) [file pone.0331965.s002.zip › cec2017/Analysis/PLOTS/C17F18.pdf]

C17F2

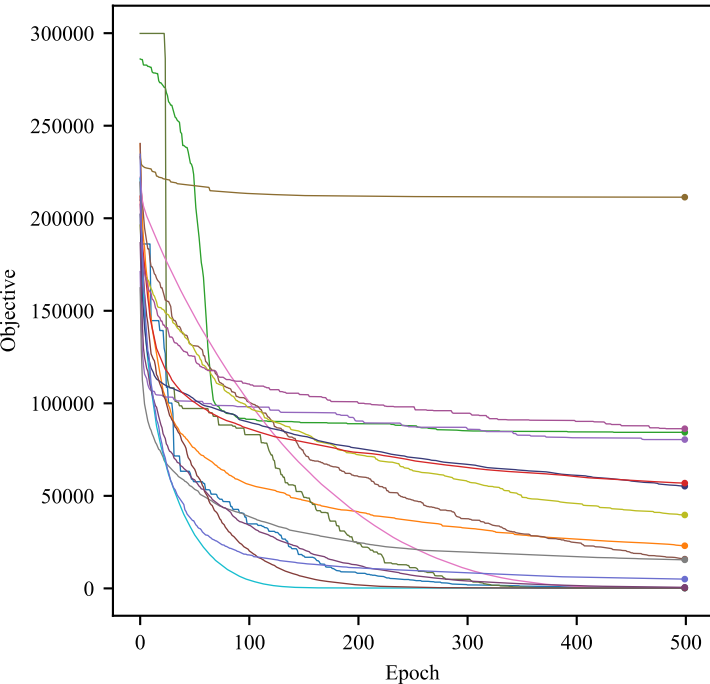

Supplement: S2 File — This compressed file contains all the results of the conducted experiments. Each main directory within the archive includes three subdirectories: Analysis, best_fit, and convergence. The best_fit and convergence folders contain the raw experimental data obtained from 30 independent runs. The Analysis folder includes tables and figures derived from these raw results. These files can be used to reproduce and further analyze the results presented in the manuscript. (ZIP) [file pone.0331965.s002.zip › cec2017/Analysis/PLOTS/C17F2.pdf]

C17F17

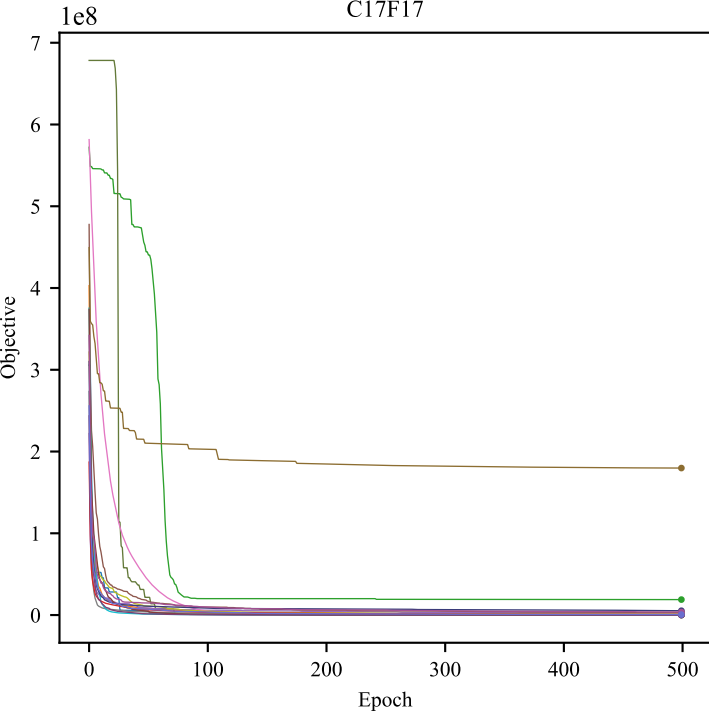

Supplement: S2 File — This compressed file contains all the results of the conducted experiments. Each main directory within the archive includes three subdirectories: Analysis, best_fit, and convergence. The best_fit and convergence folders contain the raw experimental data obtained from 30 independent runs. The Analysis folder includes tables and figures derived from these raw results. These files can be used to reproduce and further analyze the results presented in the manuscript. (ZIP) [file pone.0331965.s002.zip › cec2017/Analysis/PLOTS/C17F17.pdf]

C17F21

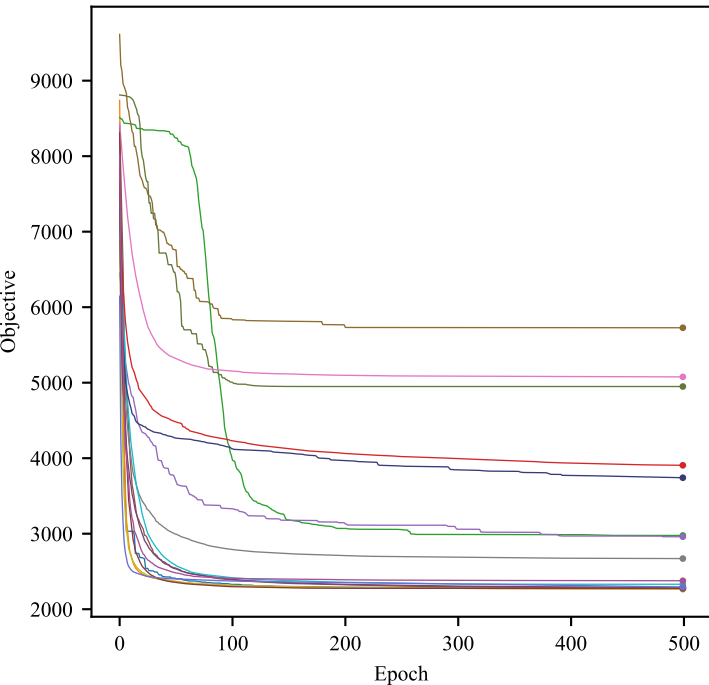

Supplement: S2 File — This compressed file contains all the results of the conducted experiments. Each main directory within the archive includes three subdirectories: Analysis, best_fit, and convergence. The best_fit and convergence folders contain the raw experimental data obtained from 30 independent runs. The Analysis folder includes tables and figures derived from these raw results. These files can be used to reproduce and further analyze the results presented in the manuscript. (ZIP) [file pone.0331965.s002.zip › cec2017/Analysis/PLOTS/C17F21.pdf]

C17F20

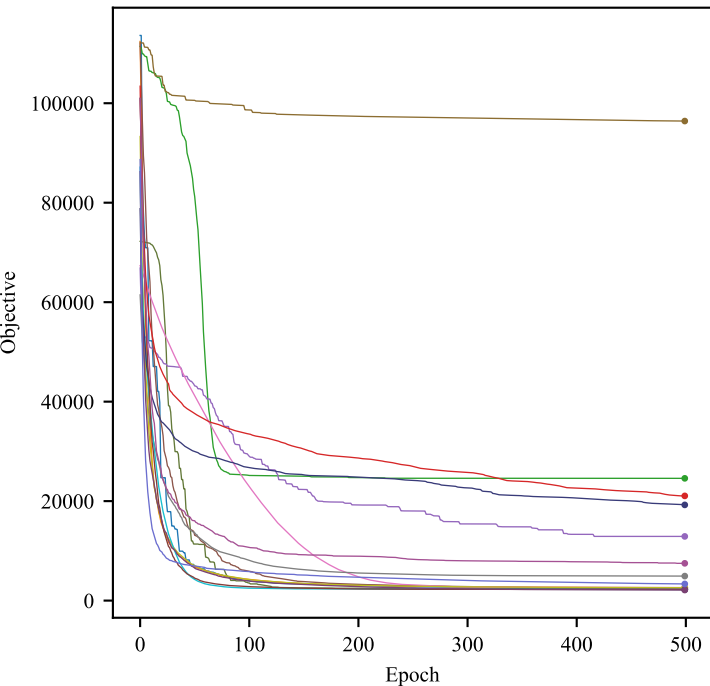

Supplement: S2 File — This compressed file contains all the results of the conducted experiments. Each main directory within the archive includes three subdirectories: Analysis, best_fit, and convergence. The best_fit and convergence folders contain the raw experimental data obtained from 30 independent runs. The Analysis folder includes tables and figures derived from these raw results. These files can be used to reproduce and further analyze the results presented in the manuscript. (ZIP) [file pone.0331965.s002.zip › cec2017/Analysis/PLOTS/C17F20.pdf]

C17F27

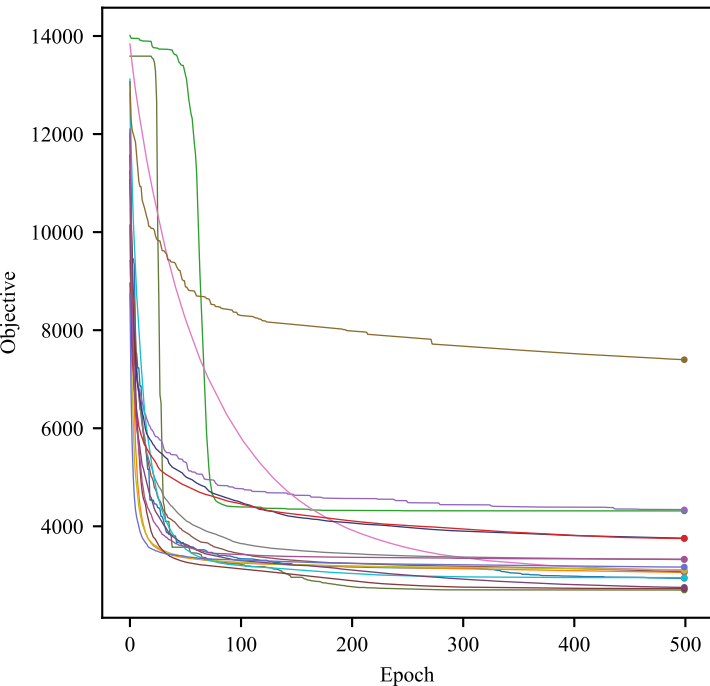

Supplement: S2 File — This compressed file contains all the results of the conducted experiments. Each main directory within the archive includes three subdirectories: Analysis, best_fit, and convergence. The best_fit and convergence folders contain the raw experimental data obtained from 30 independent runs. The Analysis folder includes tables and figures derived from these raw results. These files can be used to reproduce and further analyze the results presented in the manuscript. (ZIP) [file pone.0331965.s002.zip › cec2017/Analysis/PLOTS/C17F27.pdf]

C17F13

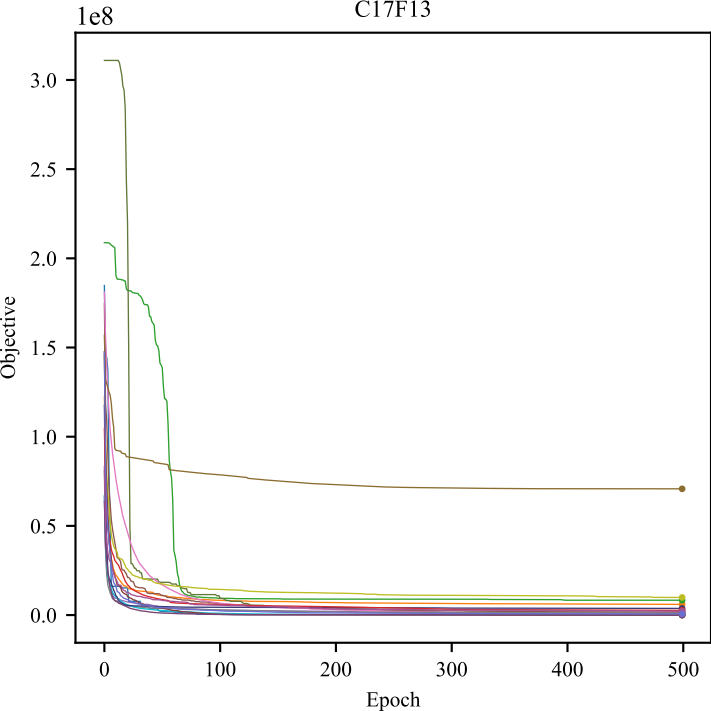

Supplement: S2 File — This compressed file contains all the results of the conducted experiments. Each main directory within the archive includes three subdirectories: Analysis, best_fit, and convergence. The best_fit and convergence folders contain the raw experimental data obtained from 30 independent runs. The Analysis folder includes tables and figures derived from these raw results. These files can be used to reproduce and further analyze the results presented in the manuscript. (ZIP) [file pone.0331965.s002.zip › cec2017/Analysis/PLOTS/C17F13.pdf]

C17F7

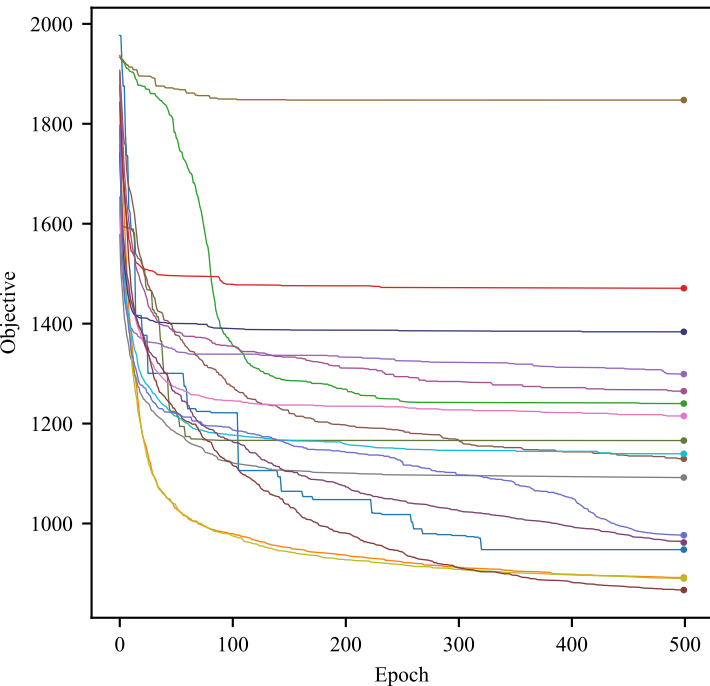

Supplement: S2 File — This compressed file contains all the results of the conducted experiments. Each main directory within the archive includes three subdirectories: Analysis, best_fit, and convergence. The best_fit and convergence folders contain the raw experimental data obtained from 30 independent runs. The Analysis folder includes tables and figures derived from these raw results. These files can be used to reproduce and further analyze the results presented in the manuscript. (ZIP) [file pone.0331965.s002.zip › cec2017/Analysis/PLOTS/C17F7.pdf]

C17F8

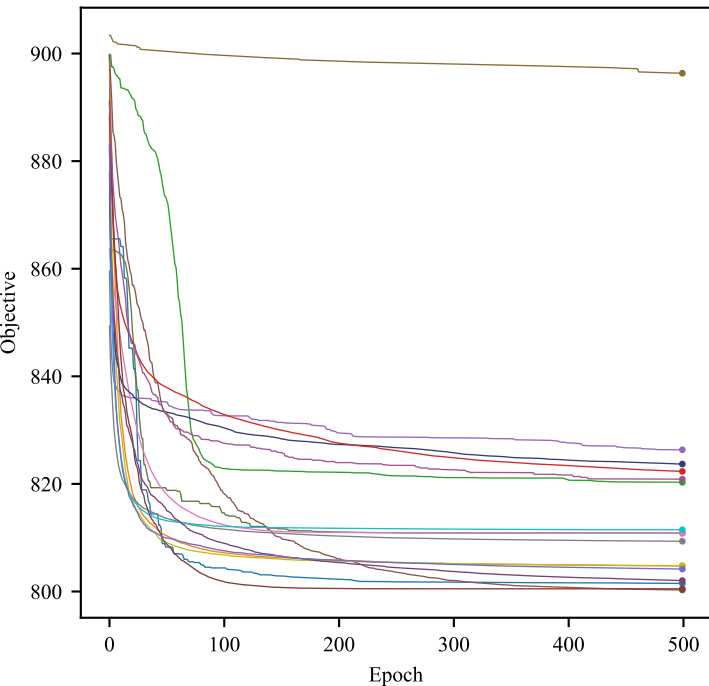

Supplement: S2 File — This compressed file contains all the results of the conducted experiments. Each main directory within the archive includes three subdirectories: Analysis, best_fit, and convergence. The best_fit and convergence folders contain the raw experimental data obtained from 30 independent runs. The Analysis folder includes tables and figures derived from these raw results. These files can be used to reproduce and further analyze the results presented in the manuscript. (ZIP) [file pone.0331965.s002.zip › cec2017/Analysis/PLOTS/C17F8.pdf]

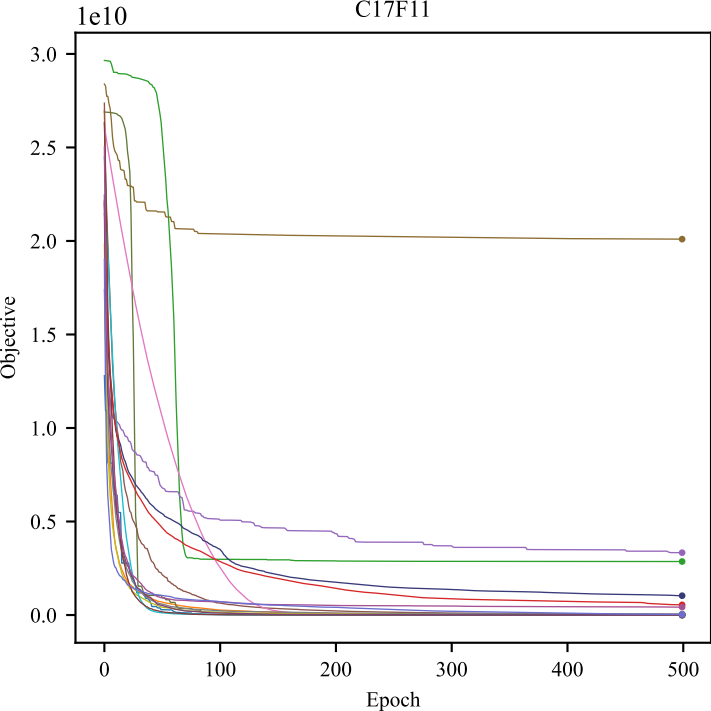

Supplement: S2 File — This compressed file contains all the results of the conducted experiments. Each main directory within the archive includes three subdirectories: Analysis, best_fit, and convergence. The best_fit and convergence folders contain the raw experimental data obtained from 30 independent runs. The Analysis folder includes tables and figures derived from these raw results. These files can be used to reproduce and further analyze the results presented in the manuscript. (ZIP) [file pone.0331965.s002.zip › cec2017/Analysis/PLOTS/C17F11.pdf]

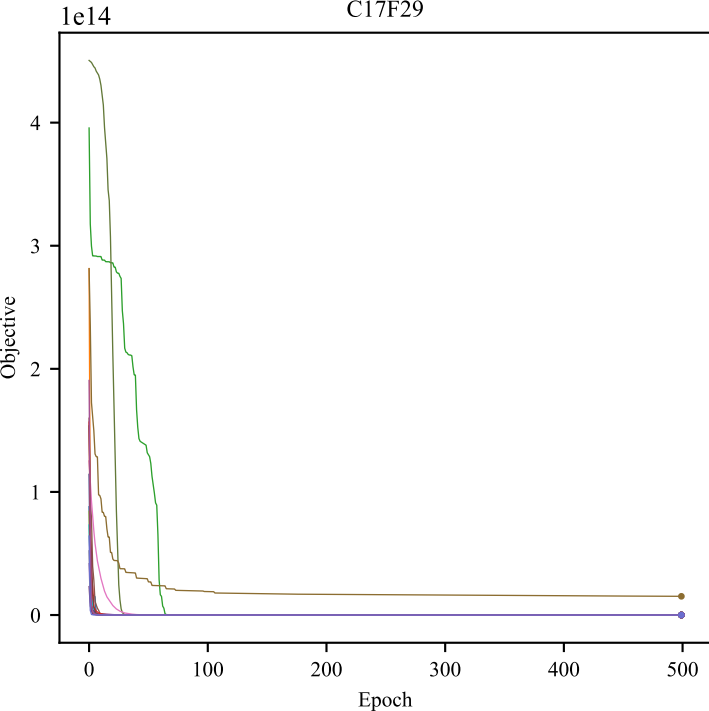

Supplement: S2 File — This compressed file contains all the results of the conducted experiments. Each main directory within the archive includes three subdirectories: Analysis, best_fit, and convergence. The best_fit and convergence folders contain the raw experimental data obtained from 30 independent runs. The Analysis folder includes tables and figures derived from these raw results. These files can be used to reproduce and further analyze the results presented in the manuscript. (ZIP) [file pone.0331965.s002.zip › cec2017/Analysis/PLOTS/C17F29.pdf]

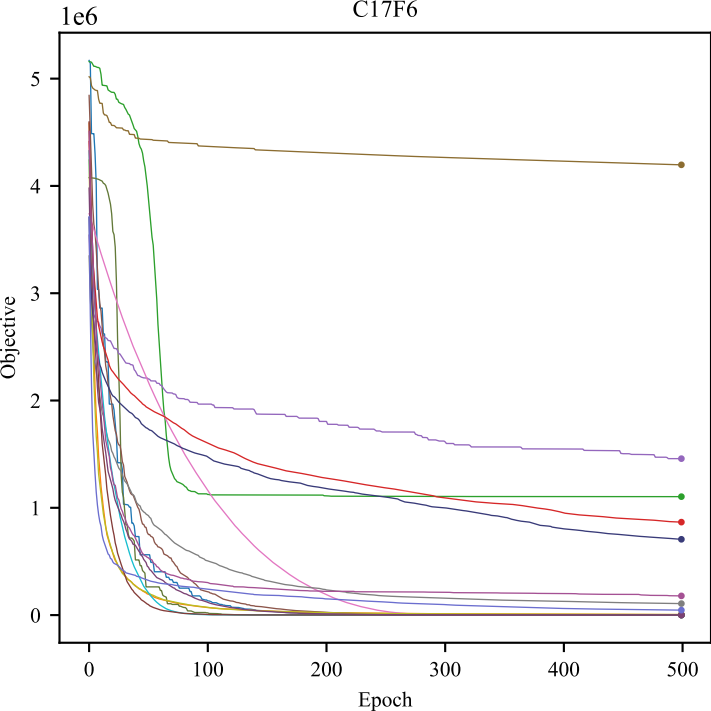

Supplement: S2 File — This compressed file contains all the results of the conducted experiments. Each main directory within the archive includes three subdirectories: Analysis, best_fit, and convergence. The best_fit and convergence folders contain the raw experimental data obtained from 30 independent runs. The Analysis folder includes tables and figures derived from these raw results. These files can be used to reproduce and further analyze the results presented in the manuscript. (ZIP) [file pone.0331965.s002.zip › cec2017/Analysis/PLOTS/C17F6.pdf]

C17F5

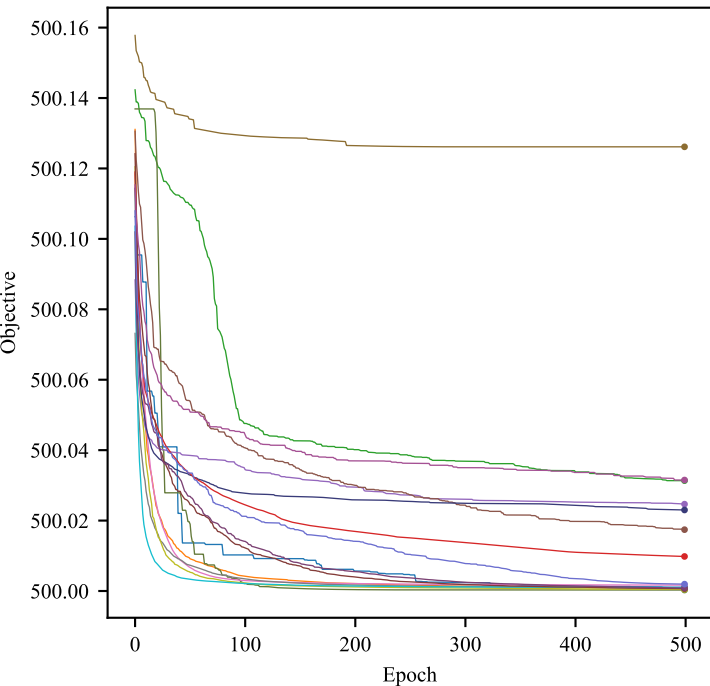

Supplement: S2 File — This compressed file contains all the results of the conducted experiments. Each main directory within the archive includes three subdirectories: Analysis, best_fit, and convergence. The best_fit and convergence folders contain the raw experimental data obtained from 30 independent runs. The Analysis folder includes tables and figures derived from these raw results. These files can be used to reproduce and further analyze the results presented in the manuscript. (ZIP) [file pone.0331965.s002.zip › cec2017/Analysis/PLOTS/C17F5.pdf]

C17F3

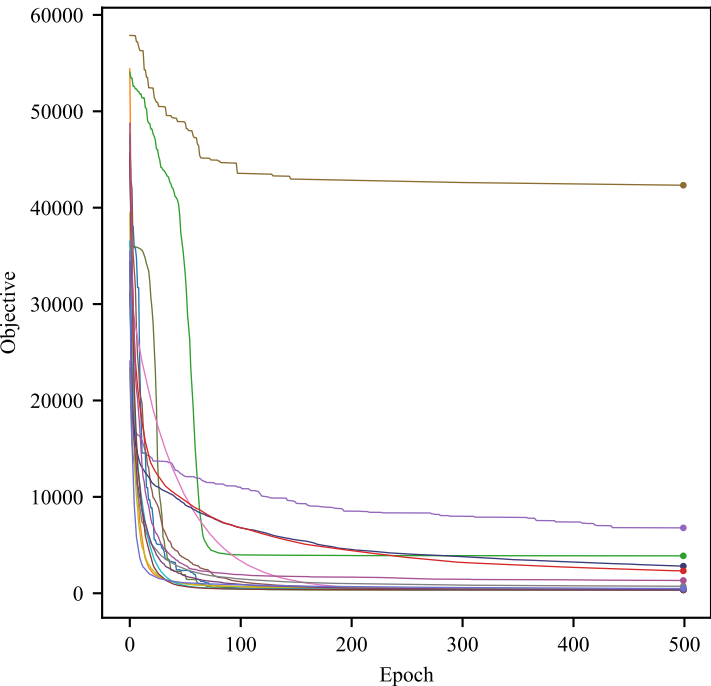

Supplement: S2 File — This compressed file contains all the results of the conducted experiments. Each main directory within the archive includes three subdirectories: Analysis, best_fit, and convergence. The best_fit and convergence folders contain the raw experimental data obtained from 30 independent runs. The Analysis folder includes tables and figures derived from these raw results. These files can be used to reproduce and further analyze the results presented in the manuscript. (ZIP) [file pone.0331965.s002.zip › cec2017/Analysis/PLOTS/C17F3.pdf]

C17F24

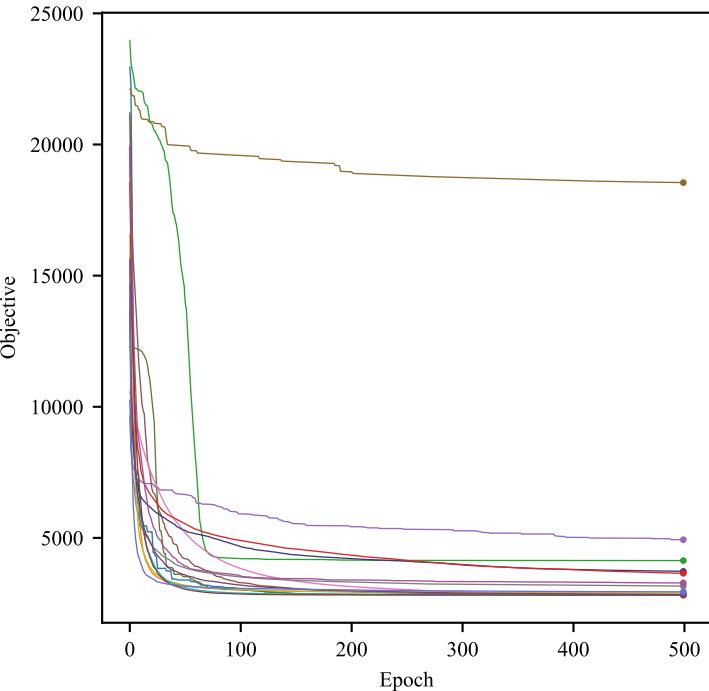

Supplement: S2 File — This compressed file contains all the results of the conducted experiments. Each main directory within the archive includes three subdirectories: Analysis, best_fit, and convergence. The best_fit and convergence folders contain the raw experimental data obtained from 30 independent runs. The Analysis folder includes tables and figures derived from these raw results. These files can be used to reproduce and further analyze the results presented in the manuscript. (ZIP) [file pone.0331965.s002.zip › cec2017/Analysis/PLOTS/C17F24.pdf]

C17F14

1e10

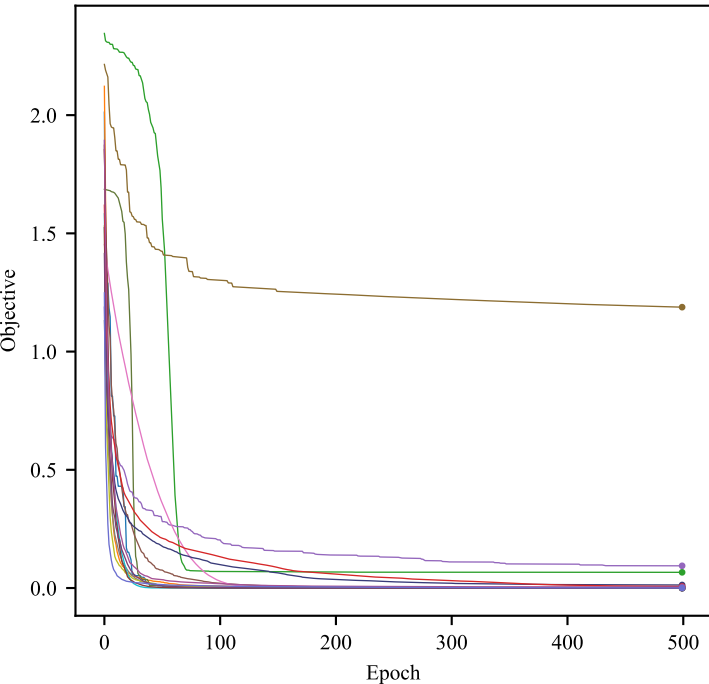

Supplement: S2 File — This compressed file contains all the results of the conducted experiments. Each main directory within the archive includes three subdirectories: Analysis, best_fit, and convergence. The best_fit and convergence folders contain the raw experimental data obtained from 30 independent runs. The Analysis folder includes tables and figures derived from these raw results. These files can be used to reproduce and further analyze the results presented in the manuscript. (ZIP) [file pone.0331965.s002.zip › cec2017/Analysis/PLOTS/C17F14.pdf]

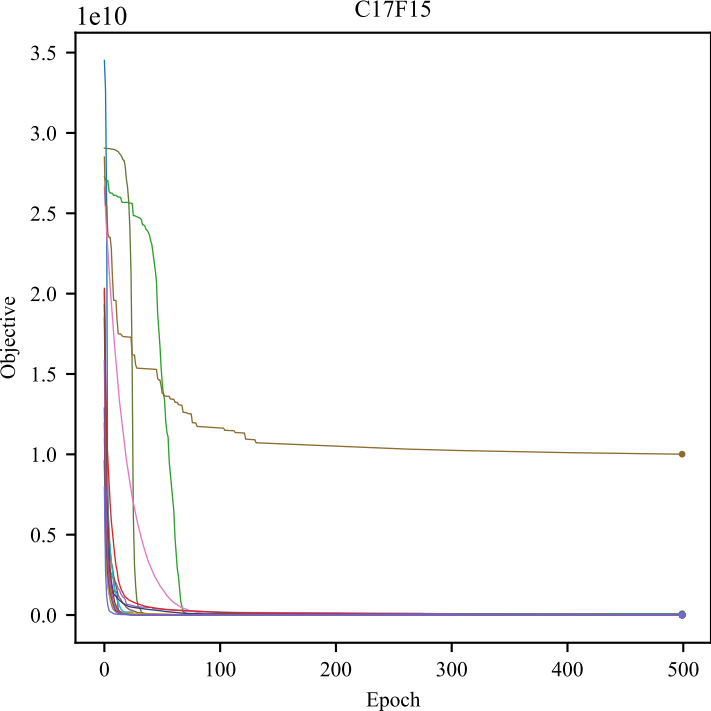

Supplement: S2 File — This compressed file contains all the results of the conducted experiments. Each main directory within the archive includes three subdirectories: Analysis, best_fit, and convergence. The best_fit and convergence folders contain the raw experimental data obtained from 30 independent runs. The Analysis folder includes tables and figures derived from these raw results. These files can be used to reproduce and further analyze the results presented in the manuscript. (ZIP) [file pone.0331965.s002.zip › cec2017/Analysis/PLOTS/C17F15.pdf]

C17F9

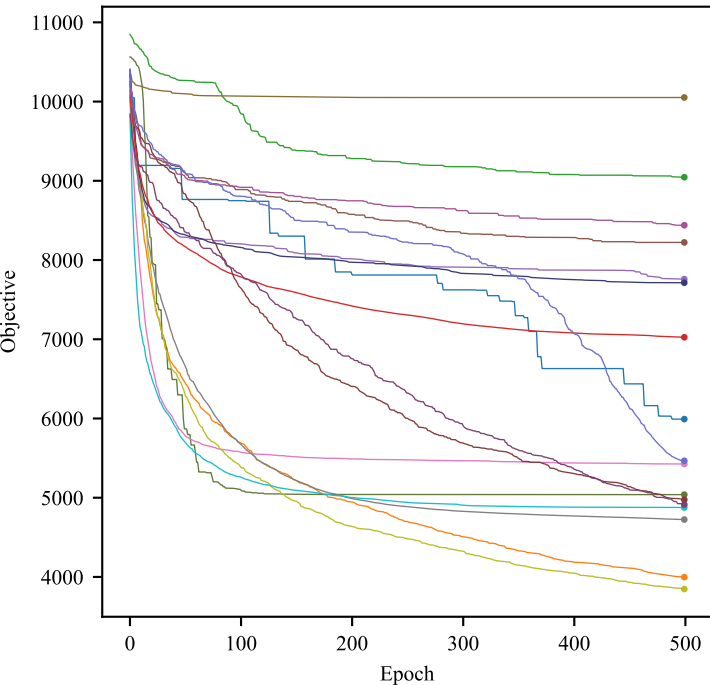

Supplement: S2 File — This compressed file contains all the results of the conducted experiments. Each main directory within the archive includes three subdirectories: Analysis, best_fit, and convergence. The best_fit and convergence folders contain the raw experimental data obtained from 30 independent runs. The Analysis folder includes tables and figures derived from these raw results. These files can be used to reproduce and further analyze the results presented in the manuscript. (ZIP) [file pone.0331965.s002.zip › cec2017/Analysis/PLOTS/C17F9.pdf]

C17F23

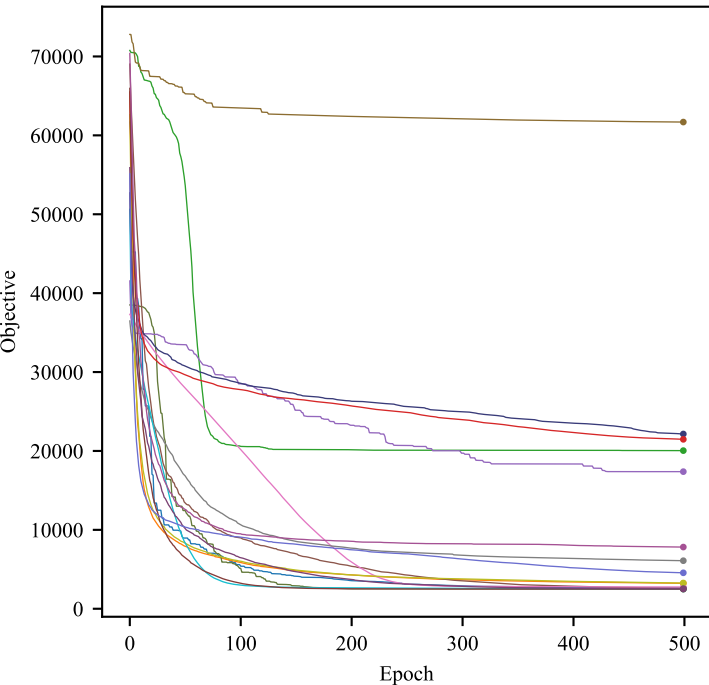

Supplement: S2 File — This compressed file contains all the results of the conducted experiments. Each main directory within the archive includes three subdirectories: Analysis, best_fit, and convergence. The best_fit and convergence folders contain the raw experimental data obtained from 30 independent runs. The Analysis folder includes tables and figures derived from these raw results. These files can be used to reproduce and further analyze the results presented in the manuscript. (ZIP) [file pone.0331965.s002.zip › cec2017/Analysis/PLOTS/C17F23.pdf]

C17F19

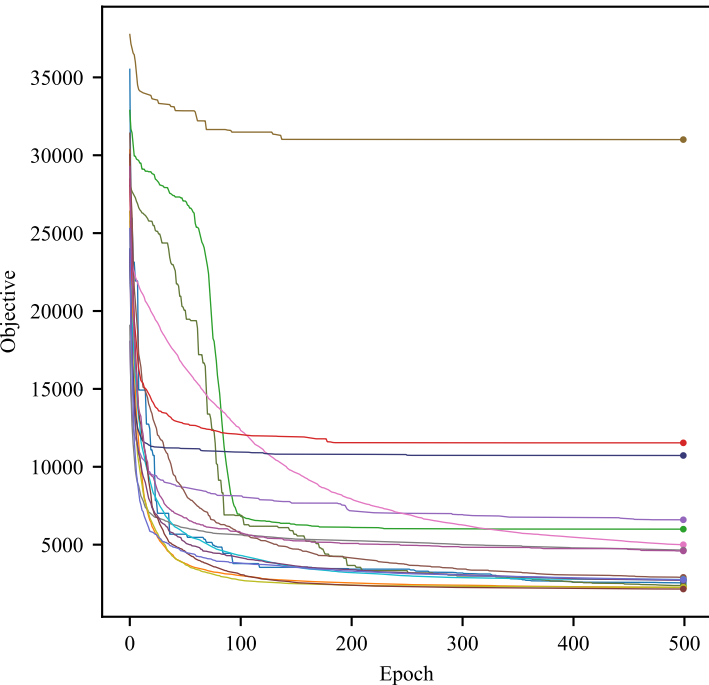

Supplement: S2 File — This compressed file contains all the results of the conducted experiments. Each main directory within the archive includes three subdirectories: Analysis, best_fit, and convergence. The best_fit and convergence folders contain the raw experimental data obtained from 30 independent runs. The Analysis folder includes tables and figures derived from these raw results. These files can be used to reproduce and further analyze the results presented in the manuscript. (ZIP) [file pone.0331965.s002.zip › cec2017/Analysis/PLOTS/C17F19.pdf]

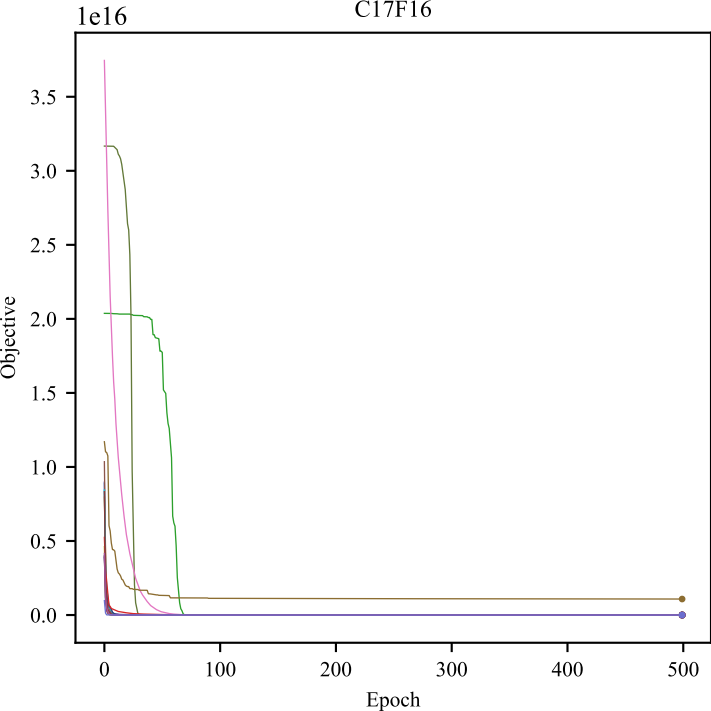

Supplement: S2 File — This compressed file contains all the results of the conducted experiments. Each main directory within the archive includes three subdirectories: Analysis, best_fit, and convergence. The best_fit and convergence folders contain the raw experimental data obtained from 30 independent runs. The Analysis folder includes tables and figures derived from these raw results. These files can be used to reproduce and further analyze the results presented in the manuscript. (ZIP) [file pone.0331965.s002.zip › cec2017/Analysis/PLOTS/C17F16.pdf]

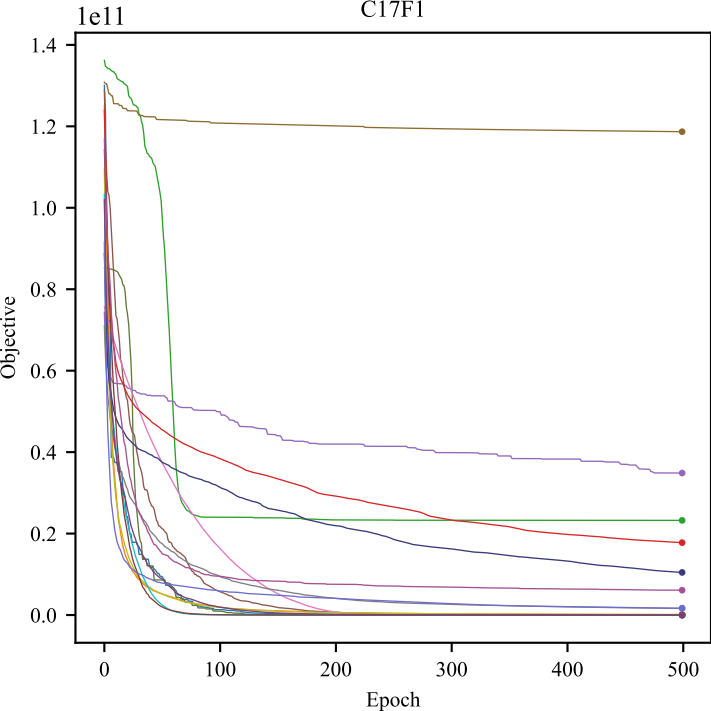

Supplement: S2 File — This compressed file contains all the results of the conducted experiments. Each main directory within the archive includes three subdirectories: Analysis, best_fit, and convergence. The best_fit and convergence folders contain the raw experimental data obtained from 30 independent runs. The Analysis folder includes tables and figures derived from these raw results. These files can be used to reproduce and further analyze the results presented in the manuscript. (ZIP) [file pone.0331965.s002.zip › cec2017/Analysis/PLOTS/C17F1.pdf]

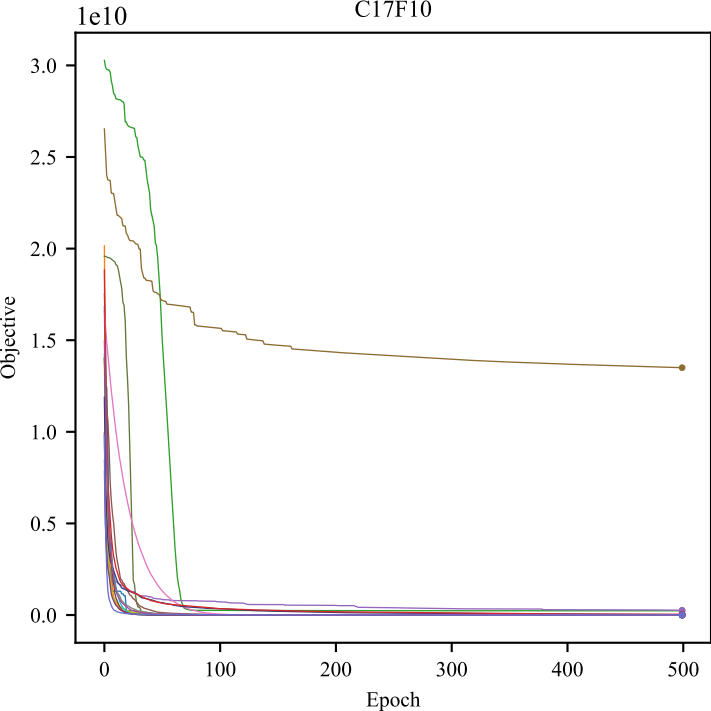

Supplement: S2 File — This compressed file contains all the results of the conducted experiments. Each main directory within the archive includes three subdirectories: Analysis, best_fit, and convergence. The best_fit and convergence folders contain the raw experimental data obtained from 30 independent runs. The Analysis folder includes tables and figures derived from these raw results. These files can be used to reproduce and further analyze the results presented in the manuscript. (ZIP) [file pone.0331965.s002.zip › cec2017/Analysis/PLOTS/C17F10.pdf]
