# Supplementary material for: An improved FOX optimization algorithm using adaptive exploration and exploitation for global optimization
Source: PLoS One. 2025 Sep 18;20(9):e0331965. doi: 10.1371/journal.pone.0331965 (PMC12445531; doi:10.1371/journal.pone.0331965)
Supplement: S2 File — This compressed file contains all the results of the conducted experiments. Each main directory within the archive includes three subdirectories: Analysis, best_fit, and convergence. The best_fit and convergence folders contain the raw experimental data obtained from 30 independent runs. The Analysis folder includes tables and figures derived from these raw results. These files can be used to reproduce and further analyze the results presented in the manuscript. (ZIP) [file pone.0331965.s002.zip › cec2019/Analysis/PLOTS/non_parametric/wilcoxon_pvalues_heatmap_annotated.pdf]

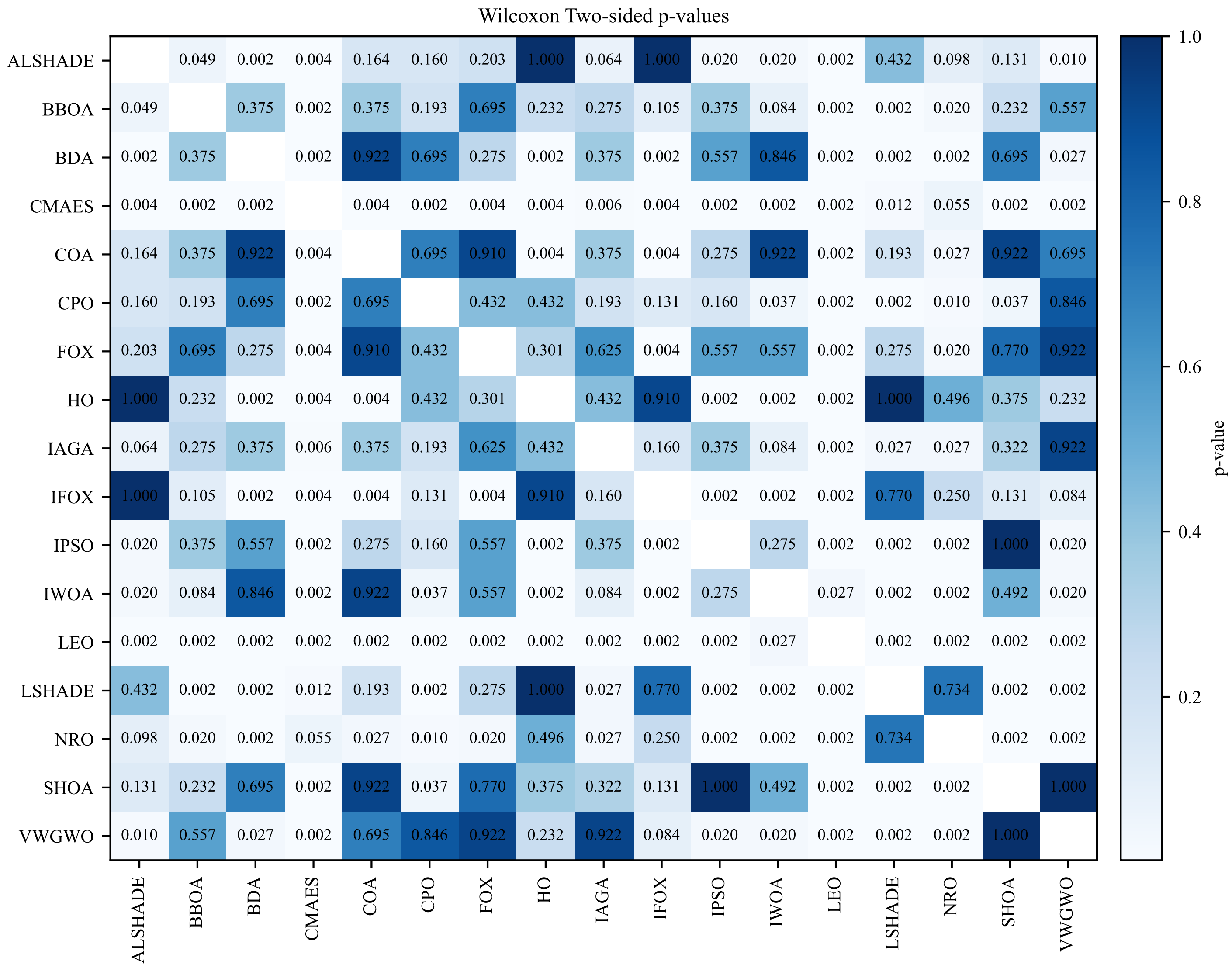
Wilcoxon Two-sided p-values
ALSHADE 0.049 0.002 0.004 0.164 0.160 0.203 1.000 0.064 1.000 0.020 0.020 0.002 0.432 0.098 0.131 0.010
BBOA 0.049 0.375 0.002 0.375 0.193 0.695 0.232 0.275 0.105 0.375 0.084 0.002 0.002 0.020 0.232 0.557
BDA 0.002 0.375 0.002 0.922 0.695 0.275 0.002 0.375 0.002 0.557 0.846 0.002 0.002 0.002 0.695 0.027
CMAES 0.004 0.002 0.002 0.004 0.002 0.004 0.004 0.006 0.004 0.002 0.002 0.002 0.012 0.055 0.002 0.002
COA 0.164 0.375 0.922 0.004 0.695 0.910 0.004 0.375 0.004 0.275 0.922 0.002 0.193 0.027 0.922 0.695
CPO 0.160 0.193 0.695 0.002 0.695 0.432 0.432 0.193 0.131 0.160 0.037 0.002 0.002 0.010 0.037 0.846
FOX 0.203 0.695 0.275 0.004 0.910 0.432 0.301 0.625 0.004 0.557 0.557 0.002 0.275 0.020 0.770 0.922
HO 1.000 0.232 0.002 0.004 0.004 0.432 0.301 0.432 0.910 0.002 0.002 0.002 1.000 0.496 0.375 0.232
IAGA 0.064 0.275 0.375 0.006 0.375 0.193 0.625 0.432 0.160 0.375 0.084 0.002 0.027 0.027 0.322 0.922
IFOX 1.000 0.105 0.002 0.004 0.004 0.131 0.004 0.910 0.160 0.002 0.002 0.002 0.770 0.250 0.131 0.084
IPSO 0.020 0.375 0.557 0.002 0.275 0.160 0.557 0.002 0.375 0.002 0.275 0.002 0.002 0.002 1.000 0.020
IWOA 0.020 0.084 0.846 0.002 0.922 0.037 0.557 0.002 0.084 0.002 0.275 0.027 0.002 0.002 0.492 0.020
LEO 0.002 0.002 0.002 0.002 0.002 0.002 0.002 0.002 0.002 0.002 0.002 0.027 0.002 0.002 0.002 0.002
LSHADE 0.432 0.002 0.002 0.012 0.193 0.002 0.275 1.000 0.027 0.770 0.002 0.002 0.002 0.734 0.002 0.002
NRO 0.098 0.020 0.002 0.055 0.027 0.010 0.020 0.496 0.027 0.250 0.002 0.002 0.002 0.734 0.002 0.002
SHOA 0.131 0.232 0.695 0.002 0.922 0.037 0.770 0.375 0.322 0.131 1.000 0.492 0.002 0.002 0.002 1.000
VWGWO 0.010 0.557 0.027 0.002 0.695 0.846 0.922 0.232 0.922 0.084 0.020 0.020 0.002 0.002 0.002 1.000
ALSHADE BBOA BDA CMAES COA CPO FOX HO IAGA IFOX IPSO IWOA LEO LSHADE NRO SHOA VWGWO
1.0
0.8
0.6
0.4
0.2
p-value
