# Supplementary material for: An improved FOX optimization algorithm using adaptive exploration and exploitation for global optimization
Source: PLoS One. 2025 Sep 18;20(9):e0331965. doi: 10.1371/journal.pone.0331965 (PMC12445531; doi:10.1371/journal.pone.0331965)
Supplement: S2 File — This compressed file contains all the results of the conducted experiments. Each main directory within the archive includes three subdirectories: Analysis, best_fit, and convergence. The best_fit and convergence folders contain the raw experimental data obtained from 30 independent runs. The Analysis folder includes tables and figures derived from these raw results. These files can be used to reproduce and further analyze the results presented in the manuscript. (ZIP) [file pone.0331965.s002.zip › cec2022/Analysis/PLOTS/non_parametric/wilcoxon_pvalues_heatmap_annotated.pdf]

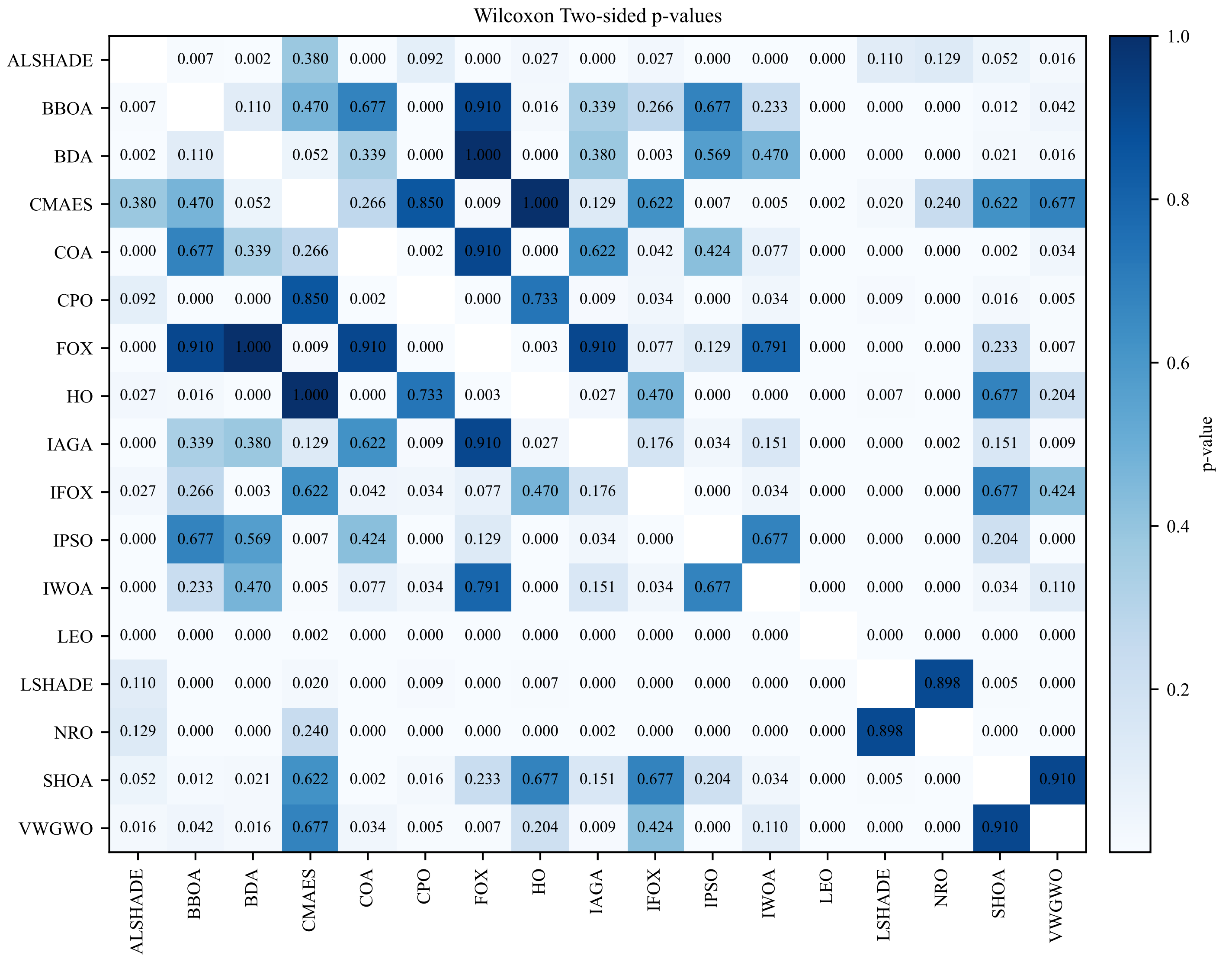
Wilcoxon Two-sided p-values
ALSHADE 0.007 0.002 0.380 0.000 0.092 0.000 0.027 0.000 0.027 0.000 0.000 0.000 0.110 0.129 0.052 0.016
BBOA 0.007 0.110 0.470 0.677 0.000 0.910 0.016 0.339 0.266 0.677 0.233 0.000 0.000 0.000 0.012 0.042
BDA 0.002 0.110 0.052 0.339 0.000 1.000 0.000 0.380 0.003 0.569 0.470 0.000 0.000 0.000 0.021 0.016
CMAES 0.380 0.470 0.052 0.266 0.850 0.009 1.000 0.129 0.622 0.007 0.005 0.002 0.020 0.240 0.622 0.677
COA 0.000 0.677 0.339 0.266 0.002 0.910 0.000 0.622 0.042 0.424 0.077 0.000 0.000 0.000 0.002 0.034
CPO 0.092 0.000 0.000 0.850 0.002 0.000 0.733 0.009 0.034 0.000 0.034 0.000 0.009 0.000 0.016 0.005
FOX 0.000 0.910 1.000 0.009 0.910 0.000 0.003 0.910 0.077 0.129 0.791 0.000 0.000 0.000 0.233 0.007
HO 0.027 0.016 0.000 1.000 0.000 0.733 0.003 0.027 0.470 0.000 0.000 0.000 0.007 0.000 0.677 0.204
IAGA 0.000 0.339 0.380 0.129 0.622 0.009 0.910 0.027 0.176 0.034 0.151 0.000 0.000 0.002 0.151 0.009
IFOX 0.027 0.266 0.003 0.622 0.042 0.034 0.077 0.470 0.176 0.000 0.034 0.000 0.000 0.000 0.677 0.424
IPSO 0.000 0.677 0.569 0.007 0.424 0.000 0.129 0.000 0.034 0.000 0.677 0.000 0.000 0.000 0.204 0.000
IWOA 0.000 0.233 0.470 0.005 0.077 0.034 0.791 0.000 0.151 0.034 0.677 0.000 0.000 0.000 0.034 0.110
LEO 0.000 0.000 0.000 0.002 0.000 0.000 0.000 0.000 0.000 0.000 0.000 0.000 0.000 0.000 0.000 0.000
LSHADE 0.110 0.000 0.000 0.020 0.000 0.009 0.000 0.007 0.000 0.000 0.000 0.000 0.000 0.898 0.005 0.000
NRO 0.129 0.000 0.000 0.240 0.000 0.000 0.000 0.000 0.002 0.000 0.000 0.000 0.000 0.898 0.000 0.000
SHOA 0.052 0.012 0.021 0.622 0.002 0.016 0.233 0.677 0.151 0.677 0.204 0.034 0.000 0.005 0.000 0.910
VWGWO 0.016 0.042 0.016 0.677 0.034 0.005 0.007 0.204 0.009 0.424 0.000 0.110 0.000 0.000 0.000 0.910
ALSHADE BBOA BDA CMAES COA CPO FOX HO IAGA IFOX IPSO IWOA LEO LSHADE NRO SHOA VWGWO
1.0
0.8
0.6
0.4
0.2
p-value
