# Supplementary material for: An improved FOX optimization algorithm using adaptive exploration and exploitation for global optimization
Source: PLoS One. 2025 Sep 18;20(9):e0331965. doi: 10.1371/journal.pone.0331965 (PMC12445531; doi:10.1371/journal.pone.0331965)
Supplement: S2 File — This compressed file contains all the results of the conducted experiments. Each main directory within the archive includes three subdirectories: Analysis, best_fit, and convergence. The best_fit and convergence folders contain the raw experimental data obtained from 30 independent runs. The Analysis folder includes tables and figures derived from these raw results. These files can be used to reproduce and further analyze the results presented in the manuscript. (ZIP) [file pone.0331965.s002.zip › classical/Analysis/PLOTS/non_parametric/friedman_ranking_heatmap_annotated.pdf]

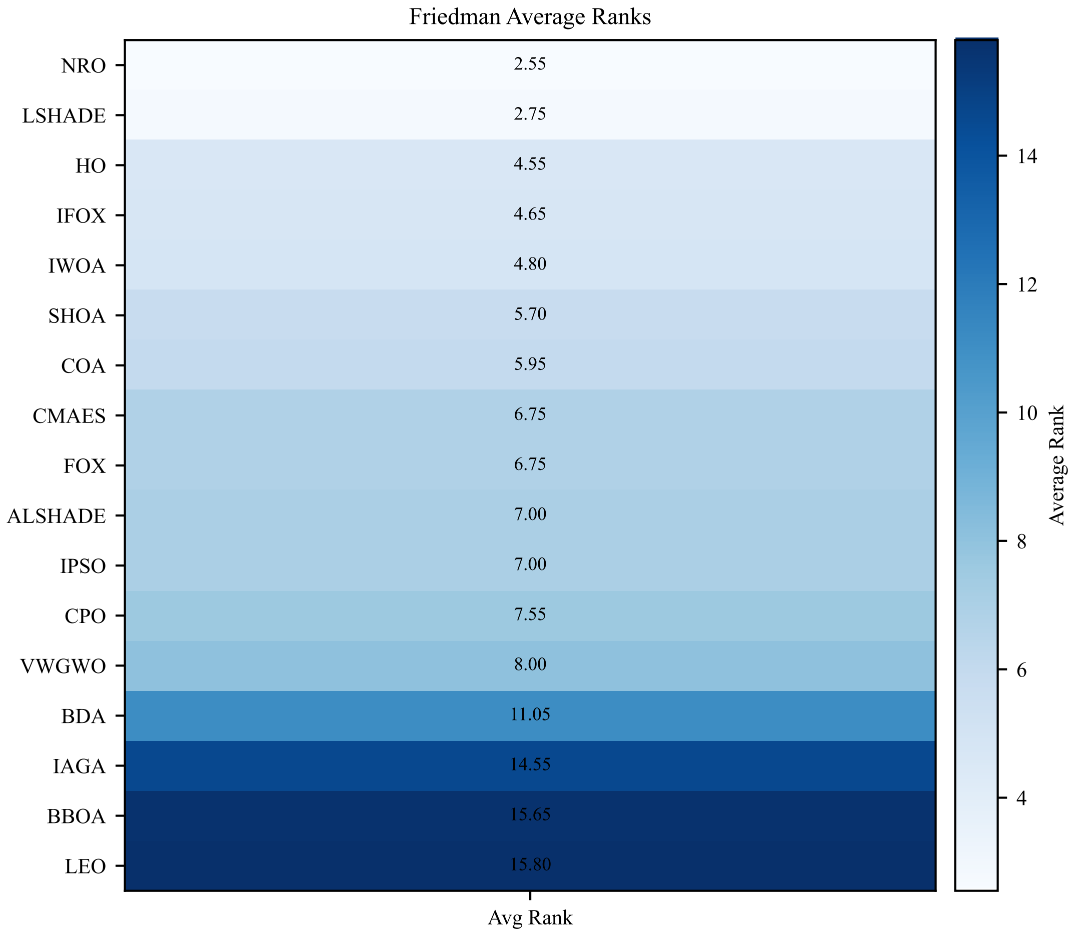

Friedman Average Ranks
NRO
LSHADE
HO
IFOX
IWOA
SHOA
COA
CMAES
FOX
ALSHADE
IPSO
CPO
VWGWO
BDA
IAGA
BBOA
LEO
2.55
2.75
4.55
4.65
4.80
5.70
5.95
6.75
6.75
7.00
7.00
7.55
8.00
11.05
14.55
15.65
15.80
Avg Rank
Average Rank
4
6
8
10
12
14
