# Supplementary material for: An improved FOX optimization algorithm using adaptive exploration and exploitation for global optimization
Source: PLoS One. 2025 Sep 18;20(9):e0331965. doi: 10.1371/journal.pone.0331965 (PMC12445531; doi:10.1371/journal.pone.0331965)
Supplement: S2 File — This compressed file contains all the results of the conducted experiments. Each main directory within the archive includes three subdirectories: Analysis, best_fit, and convergence. The best_fit and convergence folders contain the raw experimental data obtained from 30 independent runs. The Analysis folder includes tables and figures derived from these raw results. These files can be used to reproduce and further analyze the results presented in the manuscript. (ZIP) [file pone.0331965.s002.zip › classical/Analysis/PLOTS/non_parametric/wilcoxon_pvalues_heatmap_annotated.pdf]

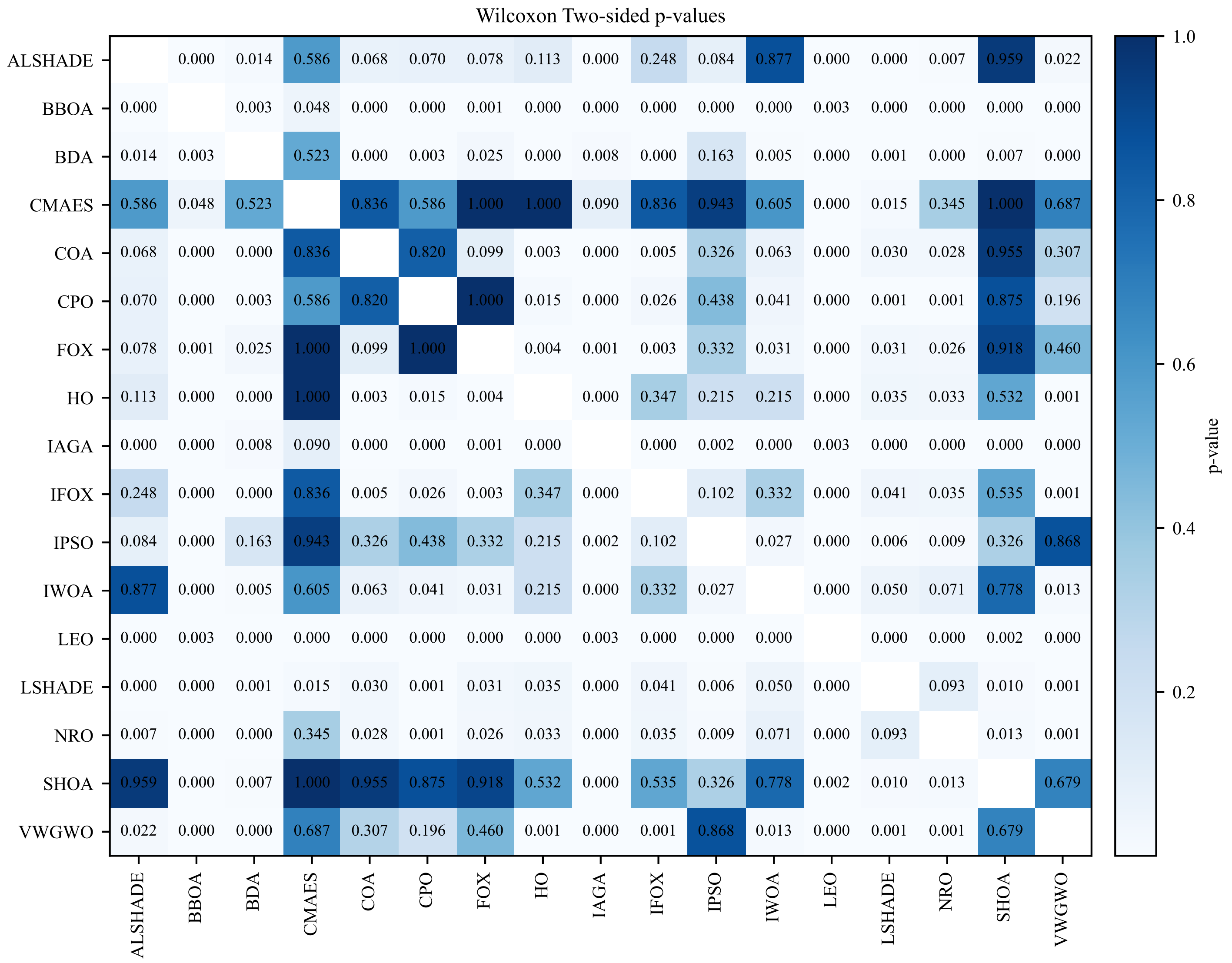
Wilcoxon Two-sided p-values
ALSHADE BBOA BDA CMAES COA CPO FOX HO IAGA IFOX IPSO IWOA LEO LSHADE NRO SHOA VWGWO
ALSHADE 0.000 0.014 0.586 0.068 0.070 0.078 0.113 0.000 0.248 0.084 0.877 0.000 0.000 0.007 0.959 0.022
BBOA 0.000 0.003 0.048 0.000 0.000 0.001 0.000 0.000 0.000 0.000 0.000 0.003 0.000 0.000 0.000 0.000
BDA 0.014 0.003 0.523 0.000 0.003 0.025 0.000 0.008 0.000 0.163 0.005 0.000 0.001 0.000 0.007 0.000
CMAES 0.586 0.048 0.523 0.836 0.586 1.000 1.000 0.090 0.836 0.943 0.605 0.000 0.015 0.345 1.000 0.687
COA 0.068 0.000 0.000 0.836 0.820 0.099 0.003 0.000 0.005 0.326 0.063 0.000 0.030 0.028 0.955 0.307
CPO 0.070 0.000 0.003 0.586 0.820 1.000 0.015 0.000 0.026 0.438 0.041 0.000 0.001 0.001 0.875 0.196
FOX 0.078 0.001 0.025 1.000 0.099 1.000 0.004 0.001 0.003 0.332 0.031 0.000 0.031 0.026 0.918 0.460
HO 0.113 0.000 0.000 1.000 0.003 0.015 0.004 0.000 0.347 0.215 0.215 0.000 0.035 0.033 0.532 0.001
IAGA 0.000 0.000 0.008 0.090 0.000 0.000 0.001 0.000 0.000 0.002 0.000 0.003 0.000 0.000 0.000 0.000
IFOX 0.248 0.000 0.000 0.836 0.005 0.026 0.003 0.347 0.000 0.102 0.332 0.000 0.041 0.035 0.535 0.001
IPSO 0.084 0.000 0.163 0.943 0.326 0.438 0.332 0.215 0.002 0.102 0.027 0.000 0.006 0.009 0.326 0.868
IWOA 0.877 0.000 0.005 0.605 0.063 0.041 0.031 0.215 0.000 0.332 0.027 0.000 0.050 0.071 0.778 0.013
LEO 0.000 0.003 0.000 0.000 0.000 0.000 0.000 0.000 0.003 0.000 0.000 0.000 0.000 0.000 0.002 0.000
LSHADE 0.000 0.000 0.001 0.015 0.030 0.001 0.031 0.035 0.000 0.041 0.006 0.050 0.000 0.093 0.010 0.001
NRO 0.007 0.000 0.000 0.345 0.028 0.001 0.026 0.033 0.000 0.035 0.009 0.071 0.000 0.093 0.013 0.001
SHOA 0.959 0.000 0.007 1.000 0.955 0.875 0.918 0.532 0.000 0.535 0.326 0.778 0.002 0.010 0.013 0.679
VWGWO 0.022 0.000 0.000 0.687 0.307 0.196 0.460 0.001 0.000 0.001 0.868 0.013 0.000 0.001 0.001 0.679
1.0
0.8
0.6
0.4
0.2
p-value
