# Supplementary material for: An improved FOX optimization algorithm using adaptive exploration and exploitation for global optimization
Source: PLoS One. 2025 Sep 18;20(9):e0331965. doi: 10.1371/journal.pone.0331965 (PMC12445531; doi:10.1371/journal.pone.0331965)
Supplement: S2 File — This compressed file contains all the results of the conducted experiments. Each main directory within the archive includes three subdirectories: Analysis, best_fit, and convergence. The best_fit and convergence folders contain the raw experimental data obtained from 30 independent runs. The Analysis folder includes tables and figures derived from these raw results. These files can be used to reproduce and further analyze the results presented in the manuscript. (ZIP) [file pone.0331965.s002.zip › real_world_problems/Analysis/PLOTS/non_parametric/wilcoxon_pvalues_heatmap_annotated.pdf]

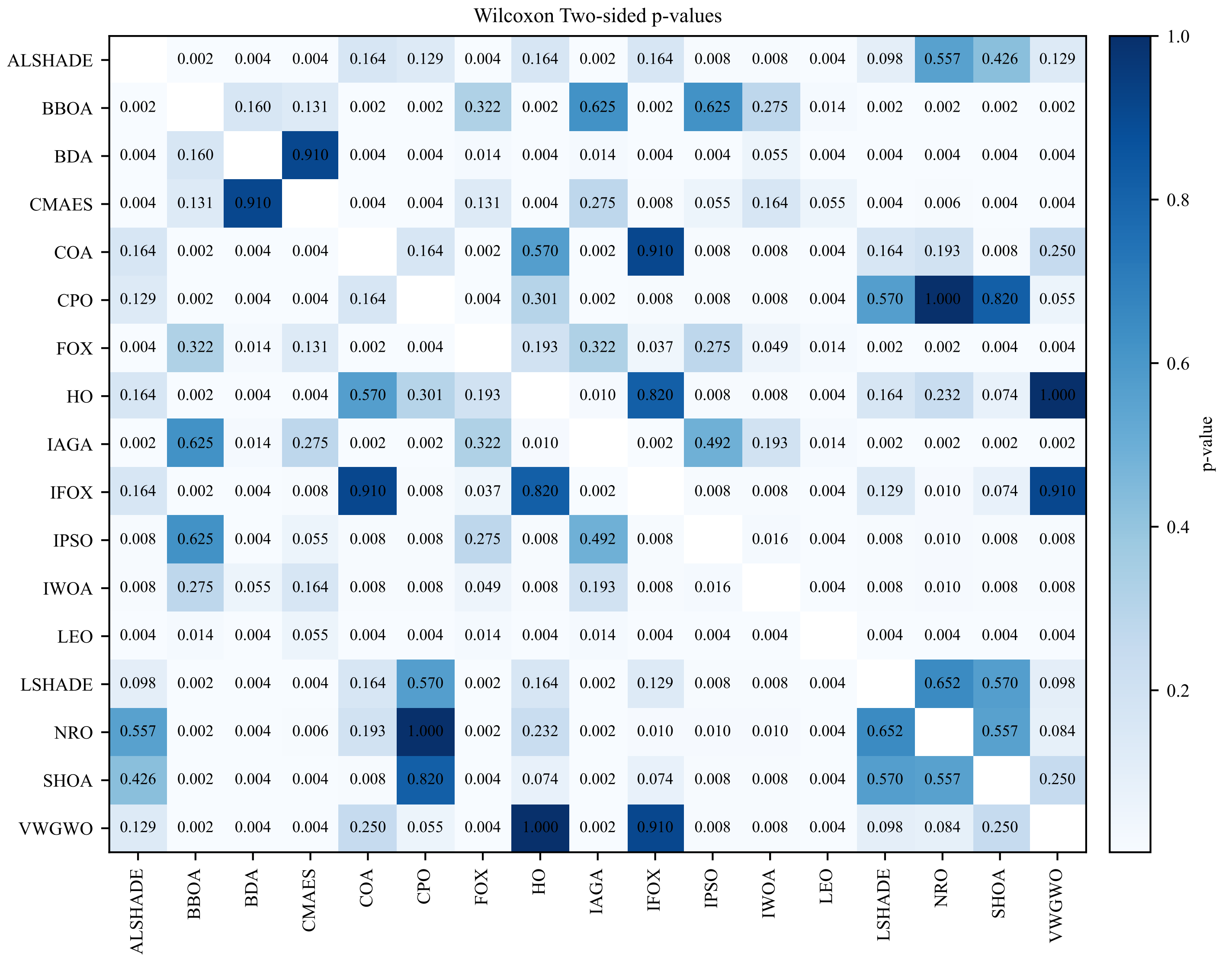
Wilcoxon Two-sided p-values
ALSHADE 0.002 0.004 0.004 0.164 0.129 0.004 0.164 0.002 0.164 0.008 0.008 0.004 0.098 0.557 0.426 0.129
BBOA 0.002 0.160 0.131 0.002 0.002 0.322 0.002 0.625 0.002 0.625 0.275 0.014 0.002 0.002 0.002 0.002
BDA 0.004 0.160 0.910 0.004 0.004 0.014 0.004 0.014 0.004 0.004 0.055 0.004 0.004 0.004 0.004 0.004
CMAES 0.004 0.131 0.910 0.004 0.004 0.131 0.004 0.275 0.008 0.055 0.164 0.055 0.004 0.006 0.004 0.004
COA 0.164 0.002 0.004 0.004 0.164 0.002 0.570 0.002 0.910 0.008 0.008 0.004 0.164 0.193 0.008 0.250
CPO 0.129 0.002 0.004 0.004 0.164 0.004 0.301 0.002 0.008 0.008 0.008 0.004 0.570 1.000 0.820 0.055
FOX 0.004 0.322 0.014 0.131 0.002 0.004 0.193 0.322 0.037 0.275 0.049 0.014 0.002 0.002 0.004 0.004
HO 0.164 0.002 0.004 0.004 0.570 0.301 0.193 0.010 0.820 0.008 0.008 0.004 0.164 0.232 0.074 1.000
IAGA 0.002 0.625 0.014 0.275 0.002 0.002 0.322 0.010 0.002 0.492 0.193 0.014 0.002 0.002 0.002 0.002
IFOX 0.164 0.002 0.004 0.008 0.910 0.008 0.037 0.820 0.002 0.008 0.008 0.004 0.129 0.010 0.074 0.910
IPSO 0.008 0.625 0.004 0.055 0.008 0.008 0.275 0.008 0.492 0.008 0.016 0.004 0.008 0.010 0.008 0.008
IWOA 0.008 0.275 0.055 0.164 0.008 0.008 0.049 0.008 0.193 0.008 0.016 0.004 0.008 0.010 0.008 0.008
LEO 0.004 0.014 0.004 0.055 0.004 0.004 0.014 0.004 0.014 0.004 0.004 0.004 0.004 0.004 0.004 0.004
LSHADE 0.098 0.002 0.004 0.004 0.164 0.570 0.002 0.164 0.002 0.129 0.008 0.008 0.004 0.652 0.570 0.098
NRO 0.557 0.002 0.004 0.006 0.193 1.000 0.002 0.232 0.002 0.010 0.010 0.010 0.004 0.652 0.557 0.084
SHOA 0.426 0.002 0.004 0.004 0.008 0.820 0.004 0.074 0.002 0.074 0.008 0.008 0.004 0.570 0.557 0.250
VWGWO 0.129 0.002 0.004 0.004 0.250 0.055 0.004 1.000 0.002 0.910 0.008 0.008 0.004 0.098 0.084 0.250
ALSHADE BBOA BDA CMAES COA CPO FOX HO IAGA IFOX IPSO IWOA LEO LSHADE NRO SHOA VWGWO
p-value
1.0 0.8 0.6 0.4 0.2
